# Toward Nano‐Nutritional Medicine: A Remotely Activated *Trans*‐Vaccenic Acid‐Based Lipid Nanoparticles for Enhancing Immune Checkpoint Blockade Therapy

**DOI:** 10.1002/advs.75992

**Published:** 2026-06-09

**Authors:** Kang Liu, Yunlong Li, Hanmeng Liu, Peng Zhang, Mingjing Wang, Quanwei Sun, Wenshuo Yang, Zhengwei Song, Song Tan, Ye Yang, Peng She, Dengke Yin, Wei Shen

**Affiliations:** ^1^ School of Pharmacy Anhui University of Chinese Medicine Hefei China; ^2^ Key Laboratory of Xin'an Medicine (Anhui University of Chinese Medicine) Hefei P. R. China; ^3^ The Seventh Affiliated Hospital of Sun Yat‐Sen University, Department of Orthopedics Guangming District Shenzhen China; ^4^ Anhui Province Key Laboratory of Pharmaceutical Preparation Technology and Application Hefei China; ^5^ Engineering Technology Research Center of Modernized Pharmaceutics Anhui Education Department (AUCM) Hefei China; ^6^ Anhui Provincial Key Laboratory of Chinese Medicinal Formula Hefei China; ^7^ National Center for Translational Medicine (Shanghai) SHU Branch Shanghai University Shanghai China; ^8^ State Key Laboratory of Polymer science and Technology (Changchun Institute of Applied Chemistry Chinese Academy of Sciences) Changchun China

**Keywords:** cancer immunotherapy, lipid nanoparticles, photothermal therapy, T‐cell exhaustion, *trans‐vaccenic* acid

## Abstract

Using nutrients to modulate anti‐tumor immunity offers clear advantages over developing new immunotherapy drugs, yet its clinical translation is hampered by low oral delivery efficiency. Herein, we propose concept of nano‐nutritional medicine (NNM), encapsulating IR780 in lipid nanoparticles (IR780@TVA LNPs) containing a eutectic mixture of *trans*‐vaccenic acid (TVA) and stearic acid. Eutectic mixture (melting point: 40.5°C) functions as gated phase‐change material, triggered by near‐infrared light to release TVA. Compared to oral free TVA, IR780@TVA LNPs increase intratumoral TVA concentration by 21.86‐fold. IR780@TVA LNPs demonstrate excellent photothermal ablation and induce adaptive immunity. Subsequently, the released TVA not only reduces CD8^+^ T cell exhaustion by activating cAMP‐PKA‐CREB axis, but downregulates PD‐1 levels in T cells and upregulates PD‐L1 levels in tumor cells, thereby potentiating PD‐L1 blockade therapy in both “hot” and “cold” tumor models, achieving complete responses in some instances. These cured mice establish long‐term immune memory, enabling them to resist tumor rechallenge. Furthermore, IR780@TVA LNPs suppress advanced large tumors and prevent lung metastasis, demonstrating superior efficacy compared to first‐line therapy (radiotherapy + PD‐L1 blockade). Overall, this work establishes first platform for targeted delivery and on‐demand release of dietary immunonutrients, paving the way for NNM as a new direction in cancer immunotherapy.

## Graphical abstract

Eutectic mixtures from *trans*‐vaccenic acid (TVA) and stearic acid, which served as phase‐change material to encapsulate IR780, forming NIR‐responsive nanoparticles. IR780@TVA LNPs induce photothermal therapy and initiate adaptive anti‐tumor immunity. As temperature exceeds melting point of eutectic mixture, TVA was released for reprograming CD8^+^ T cells through cAMP‐PKA‐CREB axis, downregulating PD‐1 on CD8^+^ T cells, and upregulating PD‐L1 on tumor cells, thereby potentiate PDL1 blockade.

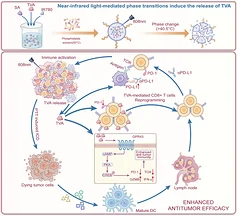

## 1 Introduction

Cancer immunotherapy has achieved significant success in treating certain types of cancer, but the limited response rate remains a critical challenge [1, 2, 3]. Research indicates that treatments such as chemotherapy, radiotherapy, and photothermal therapy (PTT) can induce tumor immunogenic cell death (ICD), resulting in the release of tumor antigens and damage‐associated molecular patterns (DAMPs) [4, 5, 6]. This process activates dendritic cells (DCs) and facilitates the recruitment and proliferation of CD8^+^ T cells, thereby enhancing the efficacy of immune checkpoint blockade therapy [7, 8, 9]. Nevertheless, the presence of an immunosuppressive tumor microenvironment (TME) and elevated expression of immune checkpoint molecules often contribute to the infiltrating T cell exhaustion, ultimately leading to cancer immune evasion [10, 11, 12]. Therefore, there is an urgent need to develop effective strategies to reprogram CD8^+^ T cells within the TME to enhance the effectiveness of immune checkpoint blockade therapy [13, 14, 15, 16].

Regulating the metabolism and function of immune cells using nutrients has emerged as a promising strategy in cancer immunotherapy in recent years [17, 18]. Recently, Chen et al. reported in *“Nature”* that *trans*‐vaccenic acid (TVA) has potential activity in promoting CD8^+^ T cell proliferation and enhancing CD8^+^ T cell function [19]. Critically, this represents the sole report to date establishing TVA as an immunonutrient capable of directly enhancing CD8^+^ T cell function. However, there is a lack of research regarding the impact of TVA on the expression levels of immune checkpoint molecules, such as PD‐1 and PD‐L1. Moreover, there are still many problems that limit its clinical application. For example, TVA, as an essential fatty acid that cannot be synthesized by humans, relies on dietary intake from ruminant sources. This poses significant challenges for achieving effective, localized, and controllable concentrations of TVA within the tumor for therapeutic purposes. In addition, oral administration of TVA requires a large dosage, which may pose potential risks of immune‐related adverse reactions. Moreover, this method of administration makes it challenging to ensure an adequate supply of TVA in real‐time during the critical period of T cell activation. Another fatal flaw of obtaining TVA through the consumption of large amounts of red meat products is the increased risk of breast cancer and colorectal cancer [20, 21]. Therefore, targeted delivery of TVA may be more effective in enhancing anti‐tumor immunity compared to dietary modifications. Although stimuli‐responsive nanoplatforms have shown remarkable success in the precise delivery of classical immunomodulatory agents (e.g., small molecule inhibitors, cytokines) [21, 22], their application for the controlled delivery and release of natural dietary nutrients to directly reprogram T cell function has, until now, remained entirely unexplored.

**SCHEME 1:**
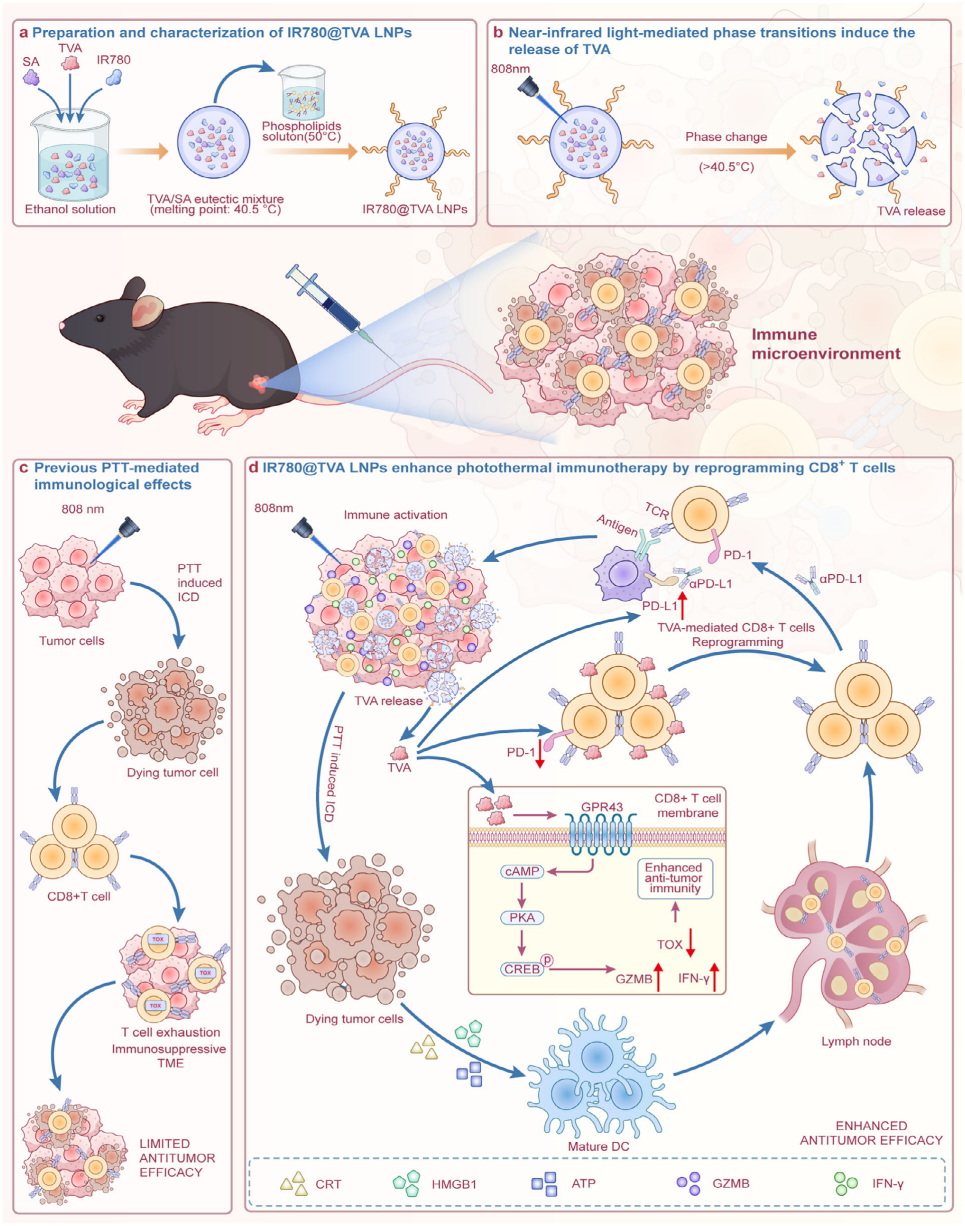
Schematic diagram of TVA‐based lipid nanoparticles (LNPs)‐mediated reprogramming of tumor cells and CD8^+^ T cells by photoinduced remote activation to enhance tumor photothermal immunotherapy. (A) Schematic diagram of the structure of IR780@TVA LNPs and their preparation process. (B) Illustration showing the near‐infrared light (NIR)‐triggered release of TVA from IR780@TVA LNPs. (C) The immune activation effects generated by traditional photothermal therapy (PTT) are limited by the exhaustion of CD8^+^ cells. (D) Herein, we encapsulated IR780 within lipid nanoparticles containing a eutectic mixture of natural TVA and SA as the core (IR780@TVA LNPs). The eutectic mixture can undergo a phase transition when subjected to NIR irradiation (melting point: 40.5°C), resulting in the precise release of TVA. Local irradiation with NIR light will induce PTT, triggering tumor ICD, and promoting T‐cell infiltration into the tumor. The released TVA can activate CD8^+^ T cells functionality and inhibit its exhaustion, thereby enhancing the efficacy of PDL1 blockade therapy.

This study introduces the concept of “nano‐nutritional medicine, NNM” by constructing a near‐infrared light (NIR)‐responsive controlled release system for the targeted delivery of TVA, aimed at enhancing the efficacy of tumor immunotherapy. As a proof of concept, we designed lipid nanoparticles (IR780@TVA LNPs) that encapsulate the NIR‐agent IR780, using a eutectic mixture of TVA and stearic acid as the core (Scheme 1A). The eutectic mixture demonstrates a specific melting point of 40.5°C, ingeniously leveraging TVA itself as a gated phase‐change material. Upon NIR irradiation, the photothermal effect‐mediated phase transition triggers precise TVA release, which subsequently reprograms cancer cells and CD8^+^ T cells. After intravenous injection, IR780@TVA LNPs accumulate at the tumor site. Subsequently, local irradiation with 808 nm NIR light will induce PTT, triggering tumor ICD and promoting T‐cell infiltration into the tumor [23, 24, 25]. Additionally, the released TVA can reprogram the tumor‐infiltrating CD8^+^ T cells by activating cAMP‐PKA‐CREB axis, reversing the exhausted phenotype of CD8^+^ T cells. Furthermore, we reveal that TVA can upregulate PD‐L1 expression in tumor cells and downregulate PD‐1 expression in CD8^+^ T cells, thereby effectively increasing sensitivity to PD‐L1 blockade therapy. The results demonstrate that IR780@TVA LNPs, when combined with anti‐PD‐L1 therapy, effectively inhibit tumor growth in both “hot” (B16F10 melanoma) and “cold” (RM‐1 prostate tumor) models. Moreover, we confirm that the IR780@TVA LNPs + anti‐PD‐L1 establishes long‐lasting immune memory, enabling cured mice to resist tumor rechallenge. Notably, IR780@TVA LNPs also effectively control the progression of advanced large tumors, showing superior efficacy compared to first‐line therapy, such as radiotherapy + PD‐L1 blockade. Overall, the IR780@TVA LNP platform obviates the need for additional pharmaceutical excipients by utilizing TVA dually as both a therapeutic immunonutrient and a gating material, thereby maximizing formulation simplicity. This remotely controlled nutrient‐releasing nano‐platform not only provides an effective nutritional intervention strategy to overcome T cell exhaustion but also pioneers a new paradigm of NNM. This nanotherapeutic platform, composed of natural food components, demonstrates considerable potential for clinical translation.

## 2 Results

### 2.1 Synthesis and Characterization of IR780@TVA LNPs

**FIGURE 1:**
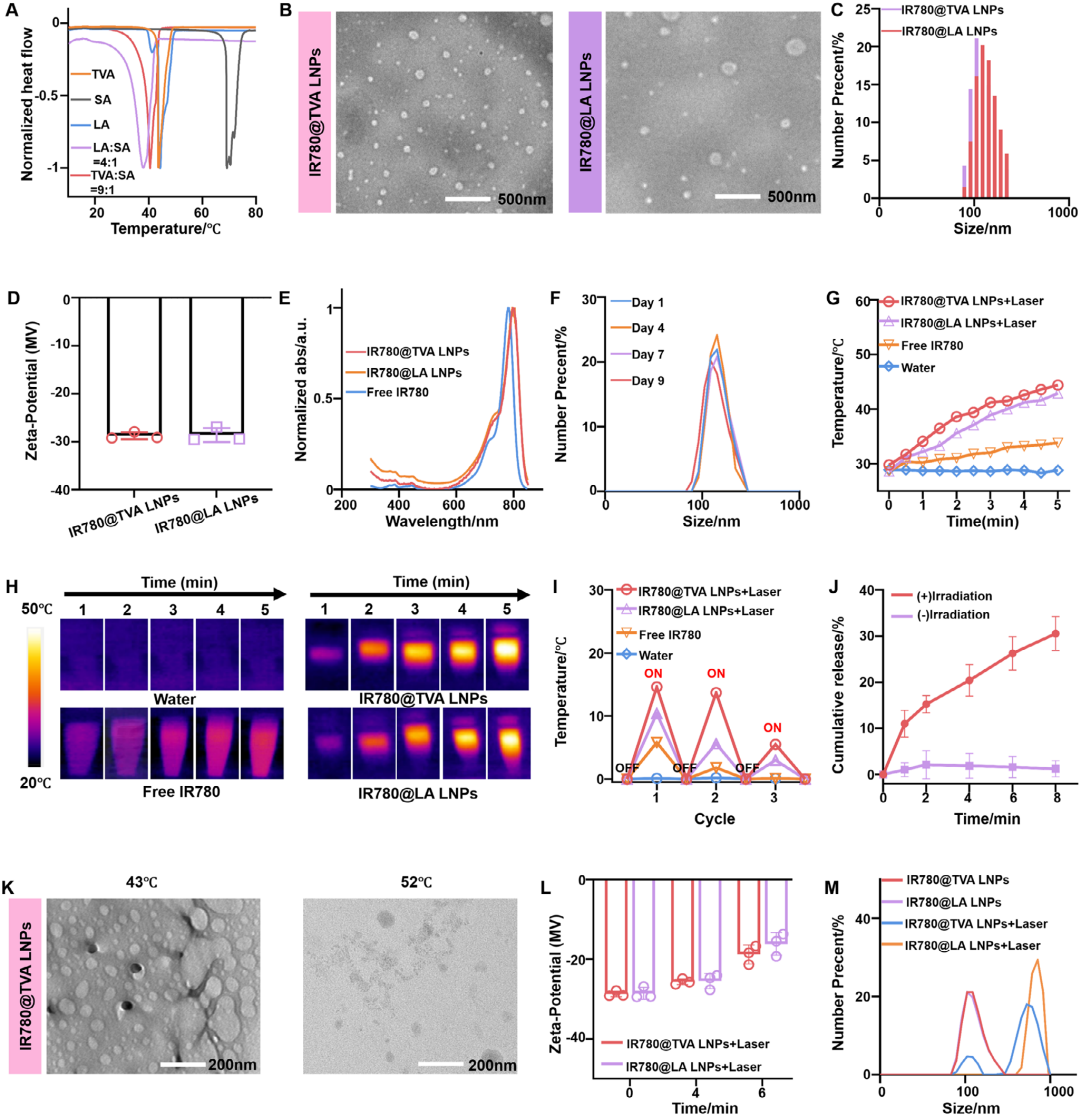
Synthesis and Characterization of IR780@TVA LNPs. (A) DSC curves of lauric acid (LA)‐stearic acid (SA) and trans‐vaccenic acid (TVA)‐stearic acid (SA) eutectic mixture at different weight ratios. (B) Representative TEM images of IR780@TVA LNPs and IR780@LA LNPs. Scale bar: 500 nm. (C) Particle size distribution of IR780@TVA LNPs and IR780@LA LNPs measured by DLS. (D) Zeta potential of IR780@TVA LNPs and IR780@LA LNPs (Data represent: mean ± SD, *n* = 3). (E) UV–vis absorption spectra of free IR780 (blue), IR780@LA LNPs (orange), and IR780@TVA LNPs (red). (F) Size stability of IR780@TVA LNPs and IR780@LA LNPs. (G) Temperature changes and (H) thermal images of water, free IR780 solution, IR780@LA LNPs, and IR780@TVA LNPs suspensions under 808 nm NIR laser irradiation at a power density of 0.5 W/cm^2^. (I) Comparison of photothermal effects of water, free IR780, IR780@LA LNPs, and IR780@TVA LNPs after repeated laser irradiation cycles (ON indicates laser irradiation/PTT on; OFF indicates laser irradiation/PTT off). (J) In vitro release profile of TVA from IR780@TVA LNPs under laser irradiation at different irradiation times (0.5 W/cm^2^) (Data represent: mean ± SD, *n* = 3). (K) TEM images of IR780@TVA LNPs at different temperatures. Scale bar: 200 nm. (L) Zeta potential of IR780@TVA LNPs and IR780@LA LNPs at different time points after 808 nm laser irradiation (Data represent: mean ± SD, *n* = 3). (M) Particle size distribution measured by DLS for IR780@TVA LNPs and IR780@LA LNPs before and after laser irradiation.

To develop appropriate gating materials for the selective release of TVA under NIR light irradiation, we first investigated the melting points of eutectic mixture with different ratios of TVA (melting point 43°C) and SA (melting point 70°C). Differential scanning calorimetry (DSC) analysis revealed that when the mass ratio of TVA to SA is 9:1, the eutectic mixture exhibits a phase transition temperature of 40.5°C (Figure 1A and Figure S1). This temperature is lower than the melting points of the individual components, which is characteristic of a typical eutectic system and falls within the temperature range suitable for PTT [26, 27]. Additionally, a mixture of lauric acid (LA) and SA (with a mass ratio of 4:1) was used as a control, which demonstrated a phase transition temperature of 39°C. Subsequently, using these two eutectic mixtures as the core, we prepared the corresponding lipid nanoparticles, IR780@TVA LNPs and IR780@LA LNPs, through a modified nanoprecipitation method (Scheme 1). Transmission electron microscopy (TEM) results indicated that the IR780@TVA LNPs exhibited a monodisperse spherical structure (Figure 1B), while dynamic light scattering (DLS) confirmed its hydrodynamic diameter to be 131.0 ± 5.5 nm, with a polydispersity index of 0.142 (Figure 1C). The Zeta potential of IR780@TVA LNPs was approximately ‐28.7 ± 0.7 mV, whereas that of IR780@LA LNPs was approximately ‐28.6 ± 1.5 mV (Figure 1D). Additionally, results from UV–vis spectroscopy confirmed the successful loading of IR780 in IR780@TVA LNPs (Figure 1E), and the drug loading capacity (DLC) of IR780 was determined to be 10.3 ± 1.9%. Meanwhile, the DLC of TVA was determined to be 4.2% ± 0.6% by using the standard curve method through gas chromatography (GC). Furthermore, the nanoparticles exhibited excellent stability in the aqueous phase, maintaining a particle size within the range of 120–150 nm after 9 days (Figure 1F). This characteristic establishes a foundation for their in vivo application in tumor‐targeted delivery.

Subsequently, we investigated the photothermal properties of the lipid nanoparticles. The results demonstrated that after irradiation with an 808 nm laser (0.5 W/cm^2^, 5 min), IR780@TVA LNPs increased the system temperature from 25.4°C to 42.3°C (Figure 1G), significantly outperforming the free IR780 group. This result was largely consistent with the temperature distribution observed in the infrared thermal imaging (Figure 1H). Moreover, the photothermal activity of free IR780 rapidly declined after just one cycle of laser exposure. In contrast, the photothermal performance of IR780@TVA LNPs in the third cycle still remained comparable to the first cycle of free IR780 group (Figure 1I). These results indicate that loading IR780 into the fatty acid eutectic mixture inhibits photobleaching and improves photothermal performance. We subsequently evaluated the drug release and responsiveness of the lipid nanoparticles. As shown in Figure 1J, the cumulative release of TVA exhibited a positive correlation with irradiation time, reaching 30.5% ± 3.7% within 8 min, while the unirradiated group released only 1.0% ± 1.7%. Additionally, we observed that when the temperature was increased to 43°C, the structure of IR780@TVA LNPs underwent swelling, with the average particle size significantly increasing from 123.0 ± 4.4 to 468.3 ± 33.0 nm, and the Zeta potential rising from ‐28.7 ± 0.7 to ‐18.8 ± 2.4 mV. As the temperature further increased to 52°C, IR780@TVA LNPs disintegrated into particles smaller than 50 nm (Figure 1K–M), likely due to structural reorganization induced by phase transition. These results confirm that the photothermal effect of IR780 can effectively trigger the melting of the eutectic mixture, thereby enabling the on‐demand release of TVA.

### 2.2 The In Vitro Cytotoxicity and ICD‐Inducing Ability of IR780@TVA LNPs

**FIGURE 2:**
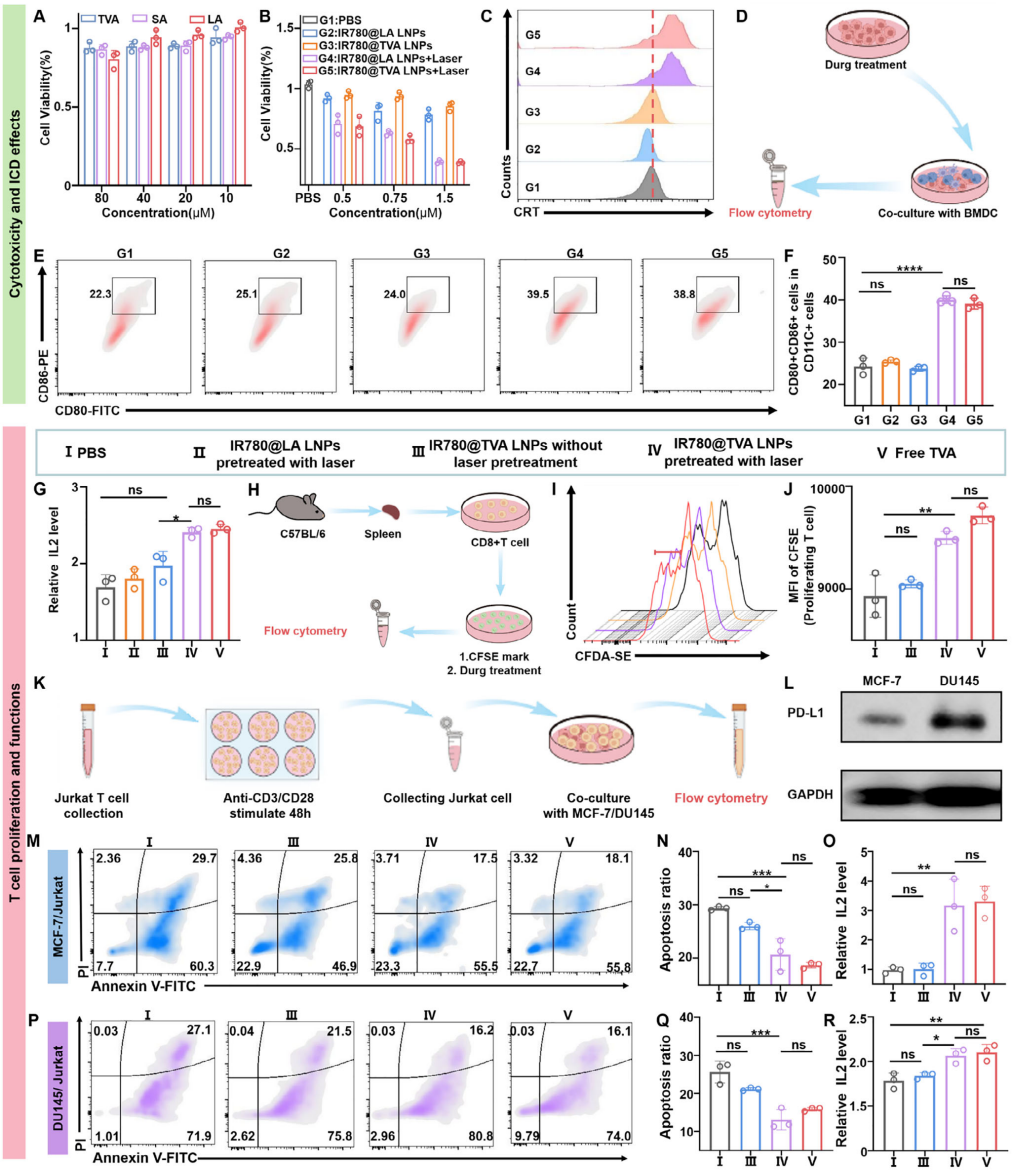
IR780@TVA LNPs induce tumor ICD and promote T cell proliferation and functions under NIR radiation in vitro. (A) Cell viability of B16F10 cells after 24 h treatment with different concentrations of TVA, LA, and SA (Data represent: mean ± SD, *n* = 3). (B) Cell viability of B16F10 cells 24 h after different treatments (Laser irradiation parameters: 808 nm, 1.0 W/cm^2^, 5 min) (Data represent: mean ± SD, *n* = 3). (C) Flow cytometry analysis of CRT exposure levels 24 h after different treatments. [PBS (G1), IR780@LA LNPs (G2), IR780@TVA LNPs (G3), IR780@LA LNPs + Laser (G4), IR780@TVA LNPs + Laser (G5).]. (D) Schematic diagram of the study on the promotion of BMDCs maturation in different treatment groups. (E, F) Flow cytometry results and relative quantitative results of BMDCs maturation after different treatments (Data represent: mean ± SD, *n* = 3). (G) IL‐2 secretion levels by Jurkat T cells after different treatments. [PBS (I), IR780@LA LNPs pretreated with laser (II), IR780@TVA LNPs without laser pretreatment (III), IR780@TVA LNPs pretreated with laser (IV), Free TVA (V) (Data represent: mean ± SD, *n* = 3).]. (H) Schematic diagram of the study on promoting the proliferation of CD8^+^ T cells after different treatments. (I, J) CD8^+^ T cell proliferation determined by flow cytometry analysis of CFSE dilution methods and the relative quantification data after different treatments for 72 h (Data represent: mean ± SD, *n* = 3). (K) Schematic diagram of the co‐culture model of DU145/MCF‐7 cells with Jurkat cells. (L) Western blotting results of PD‐L1 expression in DU145/MCF‐7 cells. (M,N) Flow cytometry results of Jurkat T cell apoptosis in the co‐culture model of Jurkat/MCF‐7 cells and the corresponding quantitative results (Data represent: mean ± SD, *n* = 3). (O) Quantitative results of IL‐2 levels in the supernatant of the Jurkat/MCF‐7 co‐culture model (Data represent: mean ± SD, *n* = 3). (P,Q) Flow cytometry results of Jurkat T cell apoptosis in the co‐culture model of Jurkat/DU145 cells and the corresponding quantitative results (Data represent: mean ± SD, *n* = 3). (R) Quantitative results of IL‐2 levels in the supernatant of the Jurkat/DU145 co‐culture model. [PBS (I), IR780@TVA LNPs without laser pretreatment (III), IR780@TVA LNPs pretreated with laser (IV), Free TVA (V) (Data represent: mean ± SD, *n* = 3).] ns: no significant difference, *p < 0.05, **p < 0.01, ***p < 0.001, ****p < 0.0001 as determined by one‐way ANOVA followed by Tukey's multiple comparison (F, G, J, N, O, Q, R).

The CCK‐8 assay confirmed that LA, SA, and TVA exhibited no cytotoxicity toward B16F10 cells (viability >80%, Figure 2A), validating the biosafety of these natural fatty acids. Additionally, IR780@LA LNPs and IR780@TVA LNPs demonstrated negligible cytotoxicity in the absence of NIR light stimulation, but exhibited significantly increased tumor cell killing efficacy upon 808 nm irradiation (Figure 2B). Flow cytometry analysis revealed that the exposure level of calreticulin (CRT) in the IR780@TVA LNPs + Laser group was 3.85 times higher than that in the control group (Figure 2C). Subsequently, the treated B16F10 cells were co‐incubated with bone marrow‐derived immature dendritic cells (BMDCs) for 24 h (Figure 2D). Flow cytometry results indicated that the promotion of BMDCs maturation (CD80^+^/CD86^+^) in the IR780@TVA LNPs + Laser group (38.8%) and IR780@LA LNPs + Laser group (39.5%) were 1.73‐fold and 1.77‐fold higher than those in the control group (22.3%) (Figure 2E,F). These results suggest that both IR780@TVA LNPs and IR780@LA LNPs are capable of killing tumor cells in vitro through PTT, and their abilities to induce ICD and activate DCs are also comparable.

### 2.3 The Impacts of IR780@TVA LNPs on T Cell Proliferation and Function In Vitro

Next, we evaluated the capacity of IR780@TVA LNPs to enhance T cell activity in vitro. Although primary T cells offer greater physiological relevance, multiple studies have demonstrated that Jurkat T cells can effectively simulate T‐cell activation, cytokine secretion, and cytotoxic functions in vitro, particularly in research related to the PD‐1/PD‐L1 immune checkpoint [28, 29, 30]. As illustrated in Figure 2G, ELISA assays indicated that the IL‐2 secretion level in the supernatant of the group III (IR780@TVA LNPs without laser pretreatment) and group II (IR780@LA LNPs pretreated with laser) was comparable to that of the control. In contrast, the IL‐2 level in the group IV (IR780@TVA LNPs pretreated with laser) exhibited a significant increase, reaching 1.6 times that of the control group, and approaching the level observed in the group V (free TVA). Furthermore, spleens were isolated from healthy C57BL/6 mice, and CD8^+^ T cells were obtained through magnetic bead separation methods. These cells were labeled with carboxyfluorescein diacetate succinimidyl ester (CFSE) and subsequently subjected to various treatments. Flow cytometry results demonstrated a significant increase in the proportion of T cells exhibiting low CFSE signal intensity in group IV and group V. This indicates that TVA released from IR780@TVA LNPs in response to NIR irradiation effectively promotes the proliferation of CD8^+^ T cells (Figure 2H–J).

Next, we established a co‐culture model of Jurkat T cells with PD‐L1 highly expressing tumor cells (DU145 human prostate cancer cell line or MCF‐7 human breast cancer cell line), to investigate whether IR780@TVA LNPs can reverse PD‐L1/PD‐1 immune checkpoint axis‐mediated T cell exhaustion (Figure 2K). Western blot results confirmed the expression of PD‐L1 on both DU145 and MCF‐7 cells (Figure 2L). The Annexin V‐FITC/PI double staining results demonstrated that in the Jurkat T/MCF‐7 cells co‐culture model, the apoptosis rate of Jurkat cells in the group IV and group V decreased from 29.7% in the control group to 17.5% and 18.1%, respectively. In contrast, the group III exhibited an apoptosis rate of 25.8%, which did not show a significant difference compared to the control group (Figure 2M,N). Concurrently, the IL‐2 concentration in the co‐culture system of the group IV increased to 3.26 times that of the control group (Figure 2O). Similar results were also validated in the Jurkat T/DU145 cells co‐culture model (Figure 2P–R).

Considering that Jurkat T cannot fully replicate the true conditions of CD8^+^ T cells, we next established a co‐culture model using primary CD8^+^ T cells isolated from mouse spleens and RM‐1 tumor cells to investigate whether TVA could restore T cell function. First, western blot analysis confirmed PD‐L1 expression on RM‐1 cells (Figure S2A, S2B). In the CD8^+^ T/RM‐1 co‐culture system, the T cell apoptosis rates in group II and group III were 83.6% and 79.2%, respectively, with no statistical difference compared with the control group. Notably, the results of group IV were consistent with above results, with CD8^+^ T cell apoptosis reduced to 71.2% (Figure S2C, S2D). These experiments indicate that TVA released from IR780@TVA LNPs in response to NIR irradiation can promote the CD8^+^ T cell proliferation, reverse T cell exhaustion, and restore T cell functions.

### 2.4 Cellular Uptake of IR780@TVA LNPs In Vitro and In Vivo

To elucidate the internalization mechanisms of our nanoparticles within key cellular components of the tumor microenvironment, we employed specific pharmacological inhibitors targeting distinct endocytic pathways [31]. In RM‐1 tumor cells, the macropinocytosis inhibitor EIPA significantly reduced nanoparticle uptake from 71.9% to 50.0%. In contrast, both the clathrin‐mediated endocytosis inhibitor (chlorpromazine, CPZ) and actin polymerization inhibitor (Cytochalasin D) showed negligible inhibitory effects. This pattern clearly establishes macropinocytosis as the predominant entry route for IR780@TVA LNPs in tumor cells (Figure S3A, S3C). Strikingly, we observed a completely different uptake profile in RAW264.7 macrophages. Here, nanoparticle internalization was predominantly suppressed by cytochalasin D, indicating phagocytosis as the dominant mechanism (Figure S3B, S3D).

To track the fate of our nanoparticles within the tumor microenvironment, we prepared Ce6‐labeled IR780@TVA LNPs, with free Ce6 serving as a control. Flow cytometric analysis was employed to quantify nanoparticle uptake in tumor cells, dendritic cells, macrophages, and CD8^+^ T cells, with the proportion of Ce6‑positive cells determined for each subset (Figure S4A–H). According to the overall distribution of nanoparticle uptake (Figure S4I), macrophages and dendritic cells exhibited the highest accumulation of LNPs, followed by tumor cells, whereas CD8^+^ T cells showed the lowest level of direct internalization among all analyzed populations. These observations confirm that CD8^+^ T cells are not the primary direct targets of the nanoplatform. Instead, myeloid cells and tumor cells constitute the major cellular reservoirs for the nanoparticles. On the basis of this uptake pattern and the mechanism of action of TVA, a “bystander effect” cascade is proposed and schematically depicted in Figure S4J. Briefly, nanoparticles are first taken up by tumor cells and myeloid cells, after which NIR irradiation induces a phase transition to liberate free TVA. The released TVA, owing to its lipophilic nature, can then diffuse across local cellular membranes into the tumor interstitial space, where it binds to membrane receptors on infiltrating CD8^+^ T cells, thereby reprogramming T‐cell function.

### 2.5 IR780@TVA LNPs Enhance CD8^+^ T Cell‐Mediated Killing of Tumor Cells by Simultaneously Activating the cAMP‐PKA‐CREB Axis and Regulating the Expression Levels of PD‐1 & PD‐L1

**FIGURE 3:**
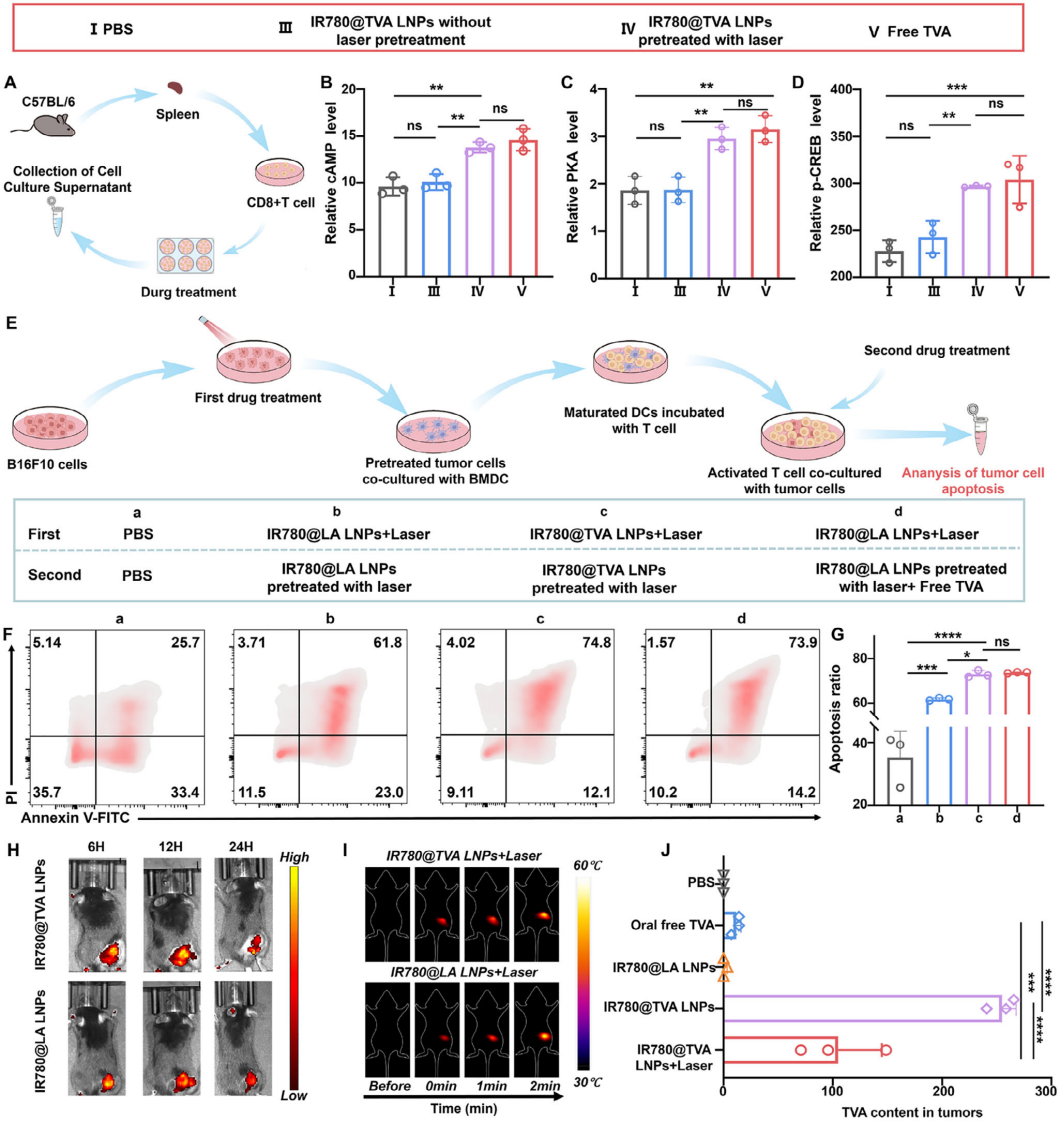
IR780@TVA LNPs can enhance CD8^+^ T cell‐mediated killing of tumor cells by activating the cAMP‐PKA‐CREB axis and effectively target tumor tissues. (A) Schematic diagram of isolation and purification of CD8^+^ T cells from the spleen of healthy mice. (B–D) The levels of cAMP, PKA, and p‐CREB in CD8^+^ T cells after different treatments (Data represent: mean ± SD, *n* = 3). [PBS (I), IR780@TVA LNPs without laser pretreatment (III), IR780@TVA LNPs pretreated with laser (IV), and Free TVA (V).] (E) Schematic diagram of the in vitro immune circulation experiment protocol, divided into 4 groups, with two steps drug treatments. [PBS/PBS (a), IR780@LA LNPs + Laser/ IR780@LA LNPs pretreated with laser (b), IR780@TVA LNPs + Laser/IR780@TVA LNPs pretreated with laser (c), IR780@LA LNPs + Laser/IR780@LA LNPs pretreated with laser + Free TVA (d).] (F) The apoptosis rate of tumor cells detected by flow cytometry and the (G) relative quantitative results after 24 h of co‐incubation with the above‐activated CD8^+^ T cells in different groups (Data represent: mean ± SD, *n* = 3). (H) In vivo biodistribution analyzed by fluorescence imaging of tumor‐bearing mice at 6, 12, and 24 h after intravenous injection of IR780@TVA LNPs and IR780@LA LNPs. (I) Thermal imaging of mice after laser irradiation (808 nm, 1.0 W/cm^2^) at 12 h post‐injection of IR780@TVA LNPs and IR780@LA LNPs. (J) Quantitative analysis of TVA levels in tumor tissues measured by gas chromatography (GC) after different treatments [*Note*: the tumor tissues were collected 2 h after irradiation (Data represent: mean ± SD, *n* = 3)]. ns: no significant difference, *p < 0.05, **p < 0.01, ***p < 0.001, ****p < 0.0001 as determined by one‐way ANOVA followed by Tukey's multiple comparison (B, C, D, G, J).

We further analyzed whether the reprogramming effect of IR780@TVA LNPs on CD8^+^ T cells is related to the activation of the cAMP‐PKA‐CREB pathway [19]. CD8^+^ T cells were isolated and purified from the splenocytes of healthy C57BL/6 mice using the magnetic bead method. Subsequently, we evaluated the activation of the cAMP‐PKA‐CREB pathway in CD8^+^ T cells following different treatments (Figure 3A). The results indicated that both the group V (free TVA) and group IV (IR780@TVA LNPs pretreated with laser) significantly increased the level of cAMP in CD8^+^ T cells (Figure 3B). As expected, this surge in cAMP levels was accompanied by an enhancement in the phosphorylation levels of protein kinase A (PKA) and its downstream effector CREB (p‐CREB), which are known to play a positive regulatory role in T cell activation (Figure 3C,D) [19]. To further validate this hypothesis, we additionally incubated T cells with the PKA inhibitor (H‐89) and the CREB inhibitor (666‐15). The results indicated that the introduction of both inhibitors significantly weakened the TVA‐mediated reprogramming effect of CD8^+^ T cells. These findings collectively establish that TVA enhances CD8^+^ T cell activity via activation of the cAMP‐PKA‐CREB signaling pathway (Figure S5A, S5B).

Furthermore, we analyzed whether TVA affects the expression levels of immune checkpoint molecules, such as PD‐1 and PD‐L1 in vitro, as these also determine the tumor‐killing activity of T cells. We first revealed that, compared to the control group (PBS or LA), TVA treatment of RM‐1 tumor cells can concentration‐dependently promote the expression of PD‐L1 on tumor cells (Figure S6A, S6B). In addition, TVA treatment significantly reduces the levels of PD‐1 on the surface of CD8^+^ T cells (Figure S6C, S6D). To further elucidate the mechanism underlying PD‑L1 upregulation in tumor cells following IR780@TVA LNPs + laser, we designed eight treatment groups: PBS (G1), IR780@LA LNPs + Laser (G2), recombinant IFN‑γ (G3), free TVA (G4), free TVA + PKA inhibitor (G5), IR780@TVA LNPs + Laser (G6), IFN‑γ + free TVA (G7), and IR780@TVA LNPs + Laser + IFN‑γ (G8). Using the G1 group as control (PD‑L1 positivity 3.3%), treatment with recombinant IFN‑γ alone (20 ng/mL, G3) increased PD‑L1 positivity to 21.5%, confirming the classical upregulatory effect of IFN‑γ. The use of free TVA (G4) alone increased the PD‐L1 positivity rate to 5.67%. In contrast, the combination of the PKA inhibitor H‐89 with free TVA (G5) resulted in a decrease in the PD‐L1 positivity rate to 1.79%. This indicates that the upregulation of PD‐L1 induced by TVA is dependent on the cAMP‐PKA signaling pathway. In addition, the group that solely mimicked PTT‐induced stress (IR780@LA LNPs + Laser, G2) dramatically increased PD‑L1 positivity to 55.5%, suggesting that photothermal stress itself is also a driver of PD‑L1 upregulation. The PD‐L1 positivity of IR780@TVA LNPs + laser (G6) is 57.2%, which is significantly higher than that of the G2 group. This indicates that photothermal‐induced stress and TVA both contribute to the upregulation of PD‐L1. When free TVA was combined with IFN‑γ (G7), PD‑L1 positivity reached 60.7%, significantly higher than either treatment alone. Finally, IR780@TVA LNPs + Laser supplemented with exogenous IFN‑γ (G8) produced the highest PD‑L1 positivity (65.9%), which indicates that the three factors—photothermal‐induced stress, TVA, and IFN‐γ—contribute to the upregulation of PD‐L1 (Figure S7A, S7B).

Based on these findings, we further evaluated the regulatory effects of IR780@TVA LNPs treatment on the PD‐1/PD‐L1 axis within tumors in vivo. Flow cytometric analysis of mouse tumors revealed that IR780@TVA LNPs treatment significantly reshaped the immune checkpoint expression profile compared to the PBS control. Specifically, the expression of PD‐L1 on tumor cells was significantly upregulated, increasing from 2.68% in the control group to 34.1% (Figure S8A, S8B). Meanwhile, the expression of PD‐1 on tumor‐infiltrating CD8^+^ T cells significantly decreased from 73.7% to 54.9% (Figure S8C, S8D). These important findings highlight the tremendous potential of TVA‐based NNM in sensitizing tumor PD‐L1 blockade therapy.

Next, we utilized an CD8^+^ T cell and tumor cell co‐incubation assay to evaluate the IR780@TVA LNPs‐triggered cycle of anti‐cancer immunity in vitro. The dying B16F10 cells, which were pretreated with different drugs, were first co‐incubated with BMDCs for 24 h (Figure 3E). Subsequently, CD8^+^ T cells isolated from the spleens of healthy mice were co‐incubated with the activated DCs for 72 h. These stimulated CD8^+^ T cells were then added to culture media containing the corresponding different drugs and co‐cultured with live B16F10 tumor cells for an additional 24 h. Finally, the apoptosis of the B16F10 cells was analyzed using flow cytometry. The results showed that the cytotoxic efficiency of CD8^+^ T cells against B16F10 tumor cells in group c (IR780@TVA LNPs + laser/IR780@TVA LNPs pretreated with laser) and group d (IR780@LA LNPs + laser/IR780@LA LNPs pretreated with laser + Free TVA) reached 74.8% and 73.9%, respectively, which is significantly higher than that of the control group (PBS/PBS, 25.7%) and group b (IR780@LA LNPs + laser/IR780@LA LNPs pretreated with laser, 61.8%) (Figure 3F,G).

Given that the previous CCK‐8 experiment demonstrated that TVA itself has no effect on the proliferation of B16F10 cells (Figure 2A), these results indicate that IR780@TVA LNPs can enhance the function of CD8^+^ T cells by releasing TVA under NIR radiation, thereby improving the tumor cell‐killing efficacy of photothermal immunotherapy.

### 2.6 IR780@TVA LNPs Show Enhanced Tumor Accumulation and Facilitate the Precise Controlled Release of TVA

Prior to evaluating the in vivo therapeutic efficacy, we established a B16F10 tumor‐bearing mouse model to investigate the tumor‐targeting capability of IR780@TVA LNPs. In vivo imaging revealed that IR780@TVA LNPs exhibited enhanced tumor accumulation and retention, which was mainly attributed to the enhanced permeability and retention (EPR) effect. Specific fluorescence accumulation was observed in the tumor region 6 h post‐intravenous injection, with a stronger fluorescence signal intensity at 12 h, Additionally, fluorescence imaging of major organs and tumor tissues at 24 h post‐injection further validated the IR780@TVA LNPs and IR780@LA LNPs can accumulate within the tumor (Figure 3H and Figure S9). To quantitatively assess the tumor‐targeting ability of IR780@TVA LNPs in an additional tumor model, we performed a parallel biodistribution study in RM‐1 prostate tumor‐bearing mice. Semi‐quantitative analysis of ex vivo organ imaging at 24 h post‐injection demonstrated that the average fluorescence intensity in the RM‐1 tumor was 1.26‐fold higher than that in the liver, 2.42‐fold higher than that in the lung, and 2.05‐fold higher than that in the kidney, further supporting the enhanced tumor accumulation of IR780@TVA LNPs (Figure S10). Under 808 nm laser irradiation, the local tumor temperature increased from 36.6 ± 0.4°C to 54.6 ± 0.6°C within 5 min in the IR780@TVA LNPs group (Figure 3I), reaching the threshold for tumor ablation [32, 33]. To further determine whether the applied laser parameters were sufficient to trigger phase transitions not only at the tumor surface but also throughout larger tumor masses, we constructed calcium alginate hydrogel phantoms containing IR780@TVA LNPs to simulate tumors of approximately 60 mm^3^ and 350 mm^3^. Under the same 808 nm laser irradiation conditions used in vivo, infrared thermal imaging showed that both the peripheral and internal temperatures of the simulated tumors rapidly increased above 42°C, exceeding the phase‐transition temperature of the TVA/SA eutectic mixture (40.5°C). Specifically, in the 60 mm^3^ phantom, the peripheral and internal temperatures reached 44.9 ± 4.2°C and 44.3 ± 1.0°C, respectively, while in the 350 mm^3^ phantom, the corresponding temperatures reached 44.8 ± 1.9°C and 42.7 ± 1.4°C (Figure S11). These results indicate that the current irradiation conditions are sufficient to achieve effective thermal activation of the phase‐change core even in relatively large tumor‐like models, thereby supporting the feasibility of intratumoral TVA release under clinically relevant tumor sizes. Additionally, we collected tumor tissues and determined the intratumoral concentration of TVA using gas chromatography. The results showed that, compared to the oral free TVA group, the intratumoral TVA concentration in the IR780@TVA LNPs group and the IR780@TVA LNPs + laser group increased by 21.86‐fold and 8.89‐fold, respectively (we speculate that NIR‐induced TVA release accelerates its tissue clearance) (Figure 3J). These data indicate that IR780@TVA LNPs can effectively initiate cancer PTT while enabling enhanced tumor accumulation and precise controlled release of TVA to reprogram intratumorally infiltrating CD8^+^ T cells. These results indicated the critical advantage of our delivery system in overcoming the inherent limitations of oral TVA. Furthermore, this underscores the necessity of proposing the concept of NNM, which is expected to open new directions for the application of nutrients in cancer immunotherapy.

### 2.7 IR780@TVA LNPs Enhance the Efficacy of Photothermal Immunotherapy for Melanoma by Augmenting CD8^+^ T Cells‐Mediated Immune Responses

**FIGURE 4:**
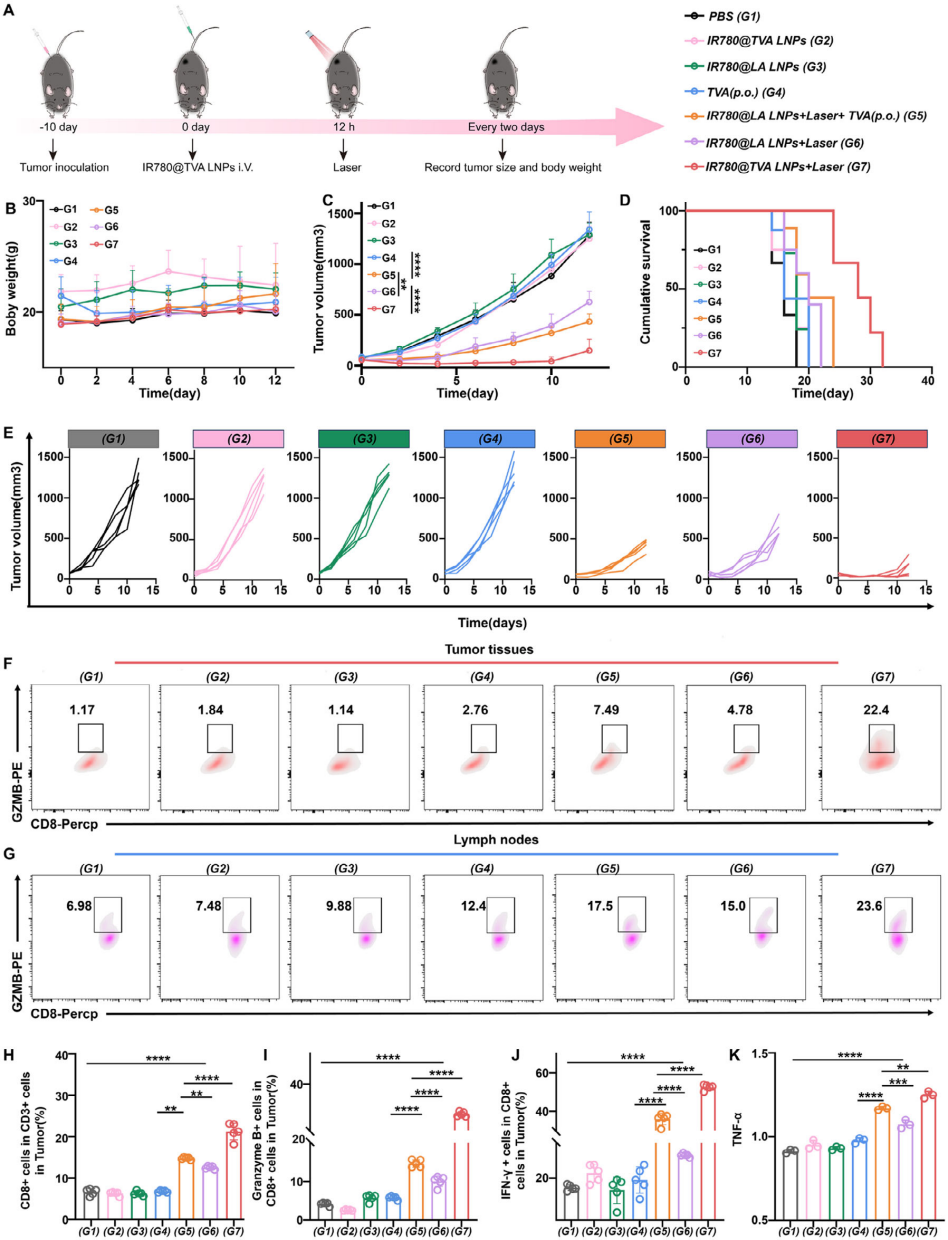
The therapeutic outcomes and immune cell analysis of tumors and tumor‐draining lymph nodes (TDLNs) in the B16F10 melanoma model. (A) Schematic diagram of the treatment regimen for the B16F10 melanoma model in mice. [PBS (G1), IR780@TVA LNPs (G2), IR780@LA LNPs (G3), TVA (p.o.) (G4), IR780@LA LNPs (i.v.) + Laser + TVA (p.o.) (G5), IR780@LA LNPs (i.v.) + Laser (G6), IR780@TVA LNPs (i.v.) + Laser (G7).] (B) Changes in body weight of mice following treatment with different groups (Data represent: mean ± SD, *n* = 5). (C) Average tumor growth curves after different treatments (Data represent: mean ± SD, *n* = 5). (D) Survival curves of mice following different treatments. (E) Individual tumor growth curves following different treatments (*n* = 5). (F) Percentage of granzyme B‐positive CD8^+^ (GZMB^+^/CD8^+^) T cells in tumors of mice on day 10 (*n* = 5). (G) Flow cytometry results of the percentage of GZMB^+^/CD8^+^ T cells in TDLNs of mice on day 10 (*n* = 5). (H–J) Relative quantification of flow cytometry analysis for the CD8^+^ T cells, GZMB^+^/CD8^+^ T cells, and IFN‐γ^+^/CD8^+^ T cells in tumors of mice (Data represent: mean ± SD, *n* = 5). (K) Levels of TNF‐α in tumor tissues after 10 days of different treatments (Data represent: mean ± SD, *n* = 3). ns: no significant difference, *p < 0.05, **p < 0.01, ***p < 0.001, ****p < 0.0001 as determined by one‑way ANOVA followed by Tukey's multiple comparison (C) or one‐way ANOVA followed by Tukey's multiple comparison (H, I, J, K).

Based on the capabilities to induce tumor ICD and activate the CD8^+^ T cell functions exhibited by IR780@TVA LNPs in vitro, we subsequently evaluated their therapeutic efficacy in a B16F10 tumor‐bearing mouse model. When the tumor volume reached 60 mm^3^, the mice were randomly divided into seven groups: (G1) PBS, (G2) IR780@TVA LNPs, (G3) IR780@LA LNPs, (G4) TVA (p.o.), (G5) IR780@LA LNPs (i.v.) + Laser + TVA (p.o.), (G6) IR780@LA LNPs (i.v.) + Laser, and (G7) IR780@TVA LNPs (i.v.) + Laser (Figure 4A). During the experimental treatment period, the body weight of the mice in each group remained stable (Figure 4B), indicating the safety of the experimental protocol. As shown in Figure 4C,E, the tumor growth curves of groups G2, G3, and G4 showed no significant difference compared to control group G1. Group G6 demonstrated moderate anti‐tumor efficacy, with a tumor inhibition rate reaching 55.40%, indicating the basic effectiveness of PTT. Additionally, we found that the tumor inhibition rate in group G5 (66.2%) improved compared to group G6, while group G7 exhibited the best anti‐tumor effect, with an inhibition rate of 88.5%. This further confirms that the NIR‐triggered TVA targeted delivery strategy is significantly superior to oral administration, thereby leading to a better cancer photothermal‐immunotherapy effect. As anticipated, G7 group significantly extended the survival of tumor‐bearing mice (Figure 4D).

Next, we evaluated the immune activation effects of IR780@TVA LNPs. Mouse tumor tissues were collected for flow cytometry analysis 10 days post NIR radiation. As illustrated in Figure S12A, the results indicate that groups G5, G6, and G7 all induced an increase in the levels of mature DCs within the tumors under NIR radiation, suggesting that PTT can trigger an anti‐tumor immune response. Furthermore, compared to group G6 (12.0%), the level of CD8^+^ T cells increased in group G5 (15.1%), while group G7 exhibited the highest level of CD8^+^ T cells (23.1%), indicating that NIR‐triggered TVA‐targeted delivery promotes CD8^+^ T cell proliferation more effectively than oral TVA. Conversely, groups G2, G3, and G4, due to the absence of PTT stimulation, showed no significant difference in CD8^+^ T cell levels compared to the control group (Figure 4H). Since the presence of an immunosuppressive TME can lead to the exhaustion of infiltrating CD8^+^ T cells, we further examined the content of functionally normal CD8^+^ T cells within tumors. As expected, the G7 group exhibited the highest levels of granzyme B‐positive CD8^+^ T cells (GZMB^+^/CD8^+^) (22.4%) and IFN‐γ‐positive CD8^+^ T cells (IFN‐γ^+^/CD8^+^) (53.8%) (Figure 4F,I,J). Meanwhile, the tumor tissues of the G7 group mice exhibited the highest levels of TNF‐α and the fewest exhausted (TOX‐positive) CD8^+^ T cells (Figure 4K and Figure S12B).

Furthermore, we conducted flow cytometry analysis on the TdLNs after different treatment. Consistent with the previously mentioned results, the IR780@TVA LNPs combined with laser treatment exhibited the highest levels of CD8^+^ T cells and GZMB^+^/CD8^+^ T cells (Figure 4G and Figure S12C, S12D). These findings confirm that under NIR irradiation, IR780@TVA LNPs can initiate an anti‐tumor immunity through the photothermal ablation while achieving precise release of TVA to enhance CD8^+^ T cell‐mediated anti‐tumor immune responses.

### 2.8 IR780@TVA LNPs Enhance the Efficacy of PD‐L1 Blockade Therapy for Melanoma and Inhibit Tumor Recurrence

**FIGURE 5:**
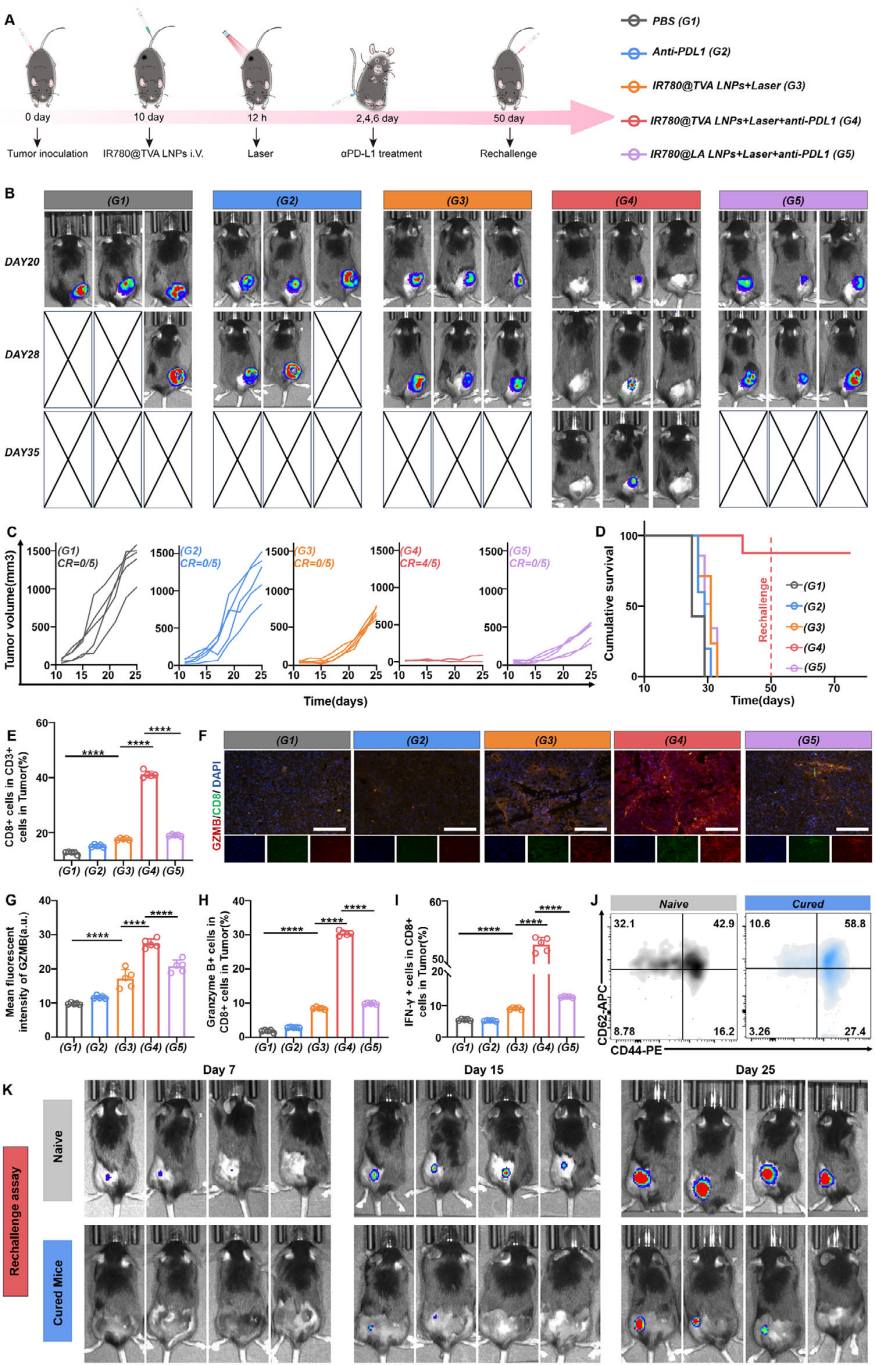
IR780@TVA LNPs enhance the efficacy of PD‐L1 blockade therapy for melanoma and inhibit tumor recurrence. (A) Schematic diagram of the treatment regimen for the B16F10 melanoma model, divided into 5 groups: PBS (G1), Anti‐PD‐L1 (G2), IR780@TVA LNPs+Laser (G3), IR780@TVA LNPs+Laser+anti‐PD‐L1 (G4), and IR780@LA LNPs+Laser+anti‐PD‐L1 (G5), (*n* = 5). (B) In vivo bioluminescence images of three representative mice after different treatments. (C) Individual tumor growth curves after different treatments (*n* = 5). (D) Survival curves of mice after different treatments (*n* = 5). (E) Relative quantification of the percentage of CD8^+^ T cells in tumors on day 10 by flow cytometry (Data represent: mean ± SD, *n* = 5). (F) Immunofluorescence images and (G) the relative fluorescence quantification of CD8^+^ T cells and GZMB expression in tumor tissues after different treatments (Data represent: mean ± SD, *n* = 5, scale bar: 50 µm). Relative quantification of the percentage of (H) GZMB^+^/CD8^+^ T and (I) IFN‐γ^+^/CD8^+^ T cells in tumors on day 10 by flow cytometry (Data represent: mean ± SD, *n* = 5). (J) Representative flow cytometry results of *T*_cm_ and *T*_em_ in mouse spleen tissues after different treatments (*n* = 4). (K) In vivo fluorescence imaging on days 7, 15, and 25 following the rechallenge of B16F10‐LUC cells in untreated or cured mice (*n* = 4). ns: no significant difference, *p < 0.05, **p < 0.01, ***p < 0.001, ****p < 0.0001 as determined by one‐way ANOVA followed by Tukey's multiple comparison (E, G, H, I).

While treatment with IR780@TVA LNPs + Laser significantly extended the survival of tumor‐bearing mice, complete tumor eradication was not achieved due to recurrence. Since the results showed that IR780@TVA LNPs + Laser upregulates the expression of programmed death‐ligand 1 (PD‐L1) in tumors (Figure S13). These findings suggested that rationally combining IR780@TVA LNPs + Laser with PD‐L1 blockade therapy holds promise for eliciting superior antitumor responses. To evaluate this, we further established luciferase‐labeled B16F10 (B16F10‐LUC) tumor‐bearing mice to evaluate the therapeutic efficacy of the combination of IR780@TVA LNPs with anti‐PD‐L1 antibody (Figure 5A). We randomly divided the mice into five groups once the tumor volume reached 60 mm^3^: (G1) PBS, (G2) anti‐PD‐L1, (G3) IR780@TVA LNPs + Laser, (G4) IR780@TVA LNPs + Laser + anti‐PD‐L1, and (G5) IR780@LA LNPs + Laser + anti‐PD‐L1. In comparison to the PBS group, the anticancer efficacy of the G2 group was minimal. While the G3 and G5 groups initially managed to control tumor growth, recurrence was observed shortly thereafter. Notably, the G4 group (IR780@TVA LNPs + laser + anti‐PD‐L1) exhibited the most optimal tumor suppression effect (Figure 5B, C and Figure S14), with 80% (4/5) of the mice remaining tumor‐free during the 50‐day monitoring period post‐treatment, significantly prolonging the survival time of mice (Figure 5D). Meanwhile, H&E images and Ki67 images showed that the tumor tissues in G4 group manifested high tumor cell necrosis with a low Ki67 proliferation signal (Figure S15A, S15B). During the experimental treatment period, no obvious change was found in the mice's body weights after different treatments (Figure S16). Besides, no significant injury was observed in the H&E staining images of the major organs of mice in the IR780@TVA LNPs group on day 10 (Figure S17). Moreover, the biochemical tests on the blood samples of mice indicated that the alanine aminotransferase (ALT), aspartate aminotransferase (AST), albumin (ALB), and creatinine (CR) in the IR780@TVA LNPs group remained at normal levels (Figure S18), suggesting the safety of the experimental protocol.

We subsequently analyzed the immune cell phenotypes within the tumors 10 days after treatment. Flow cytometry results indicated that the G4 group exhibited the highest levels of mature DCs and CD8^+^ T cells (Figure S19 and Figure 5E). We next examined the functional activity of tumor‐infiltrating CD8^+^ T cells. The immunofluorescence staining results indicate that the G4 group exhibited the highest expression of GZMB within tumor tissue, with a relative fluorescence intensity increase of 2.78‐fold compared to the control group (Figure 5F,G). As anticipated, G4 group also had the highest content of GZMB^+^/CD8^+^ T cells (31.4%) and IFN‐γ^+^/CD8^+^ T cells (54.0%) within tumors (Figure 5H,I). Furthermore, we analyzed the proportion of immune cells (DCs, CD8^+^ T cells, GZMB^+^/CD8^+^ T cells, and IFN‐γ^+^/CD8^+^ T cells) in TdLNs and found that the results were consistent with the trends observed in the aforementioned tumor tissues (Figure S20A–S20D). These findings confirm that the NIR‐triggered TVA‐targeted delivery strategy can activate the immune response associated with PTT while reprograming CD8^+^ T cells, thereby enhancing PD‐L1 checkpoint blockade therapy.

Considering that 80% of the mice in IR780@TVA LNPs combined with anti‐PD‐L1 group were completely cured, we further investigated whether effective immune memory had been established in these cured mice. On day 50, we rechallenged these mice by subcutaneously inoculating B16F10‐LUC cells, using untreated healthy mice as the control (Figure 5A). Compared to the control mice, which exhibited rapid tumor progression, tumor recurrence was significantly suppressed in the cured mice, with 1 out of 4 mice showing no tumor recurrence 25 days after rechallenge (Figure 5K and Figure S21). As expected, flow cytometry analysis of the spleen post‐rechallenge revealed a significant increase in the percentage of central memory CD8^+^ T cells (*T*_cm_, CD3^+^CD8^+^CD44^+^CD62L^+^) and effector memory CD8^+^ T cells (T_em_, CD3^+^CD8^+^CD44^+^CD62L^−^) in the cured mice group (Figure 5J and Figure S22). These results confirm that the combination therapy of IR780@TVA LNPs with anti‐PD‐L1 not only promotes the regression of primary tumors but also helps establish long‐lasting immune memory protection, thereby reducing tumor recurrence. However, it should be noted that while *n* = 4 reflects the actual number of cured mice, it does limit the statistical confidence to some extent.

### 2.9 IR780@TVA LNPs can also Enhance CD8^+^ T Cells‐Mediated Immune Responses in the Immunologically “Cold” Prostate Cancer Model

**FIGURE 6:**
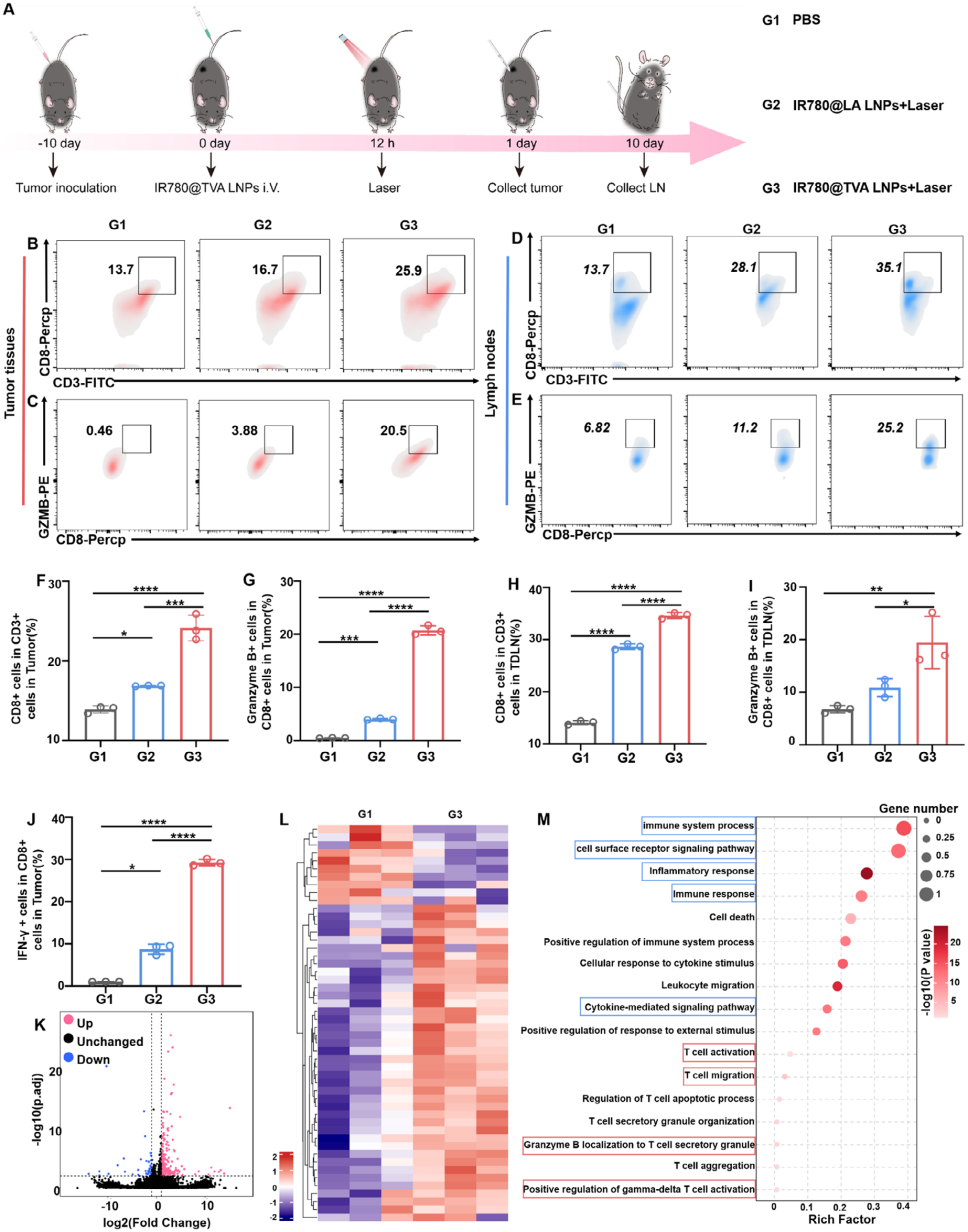
IR780@TVA LNPs reverse prostate cancer from “cold” to “hot” inflamed tumor, and the transcriptome analysis of tumor tissue. (A) Schematic of the treatment regimen for the mouse RM‐1 prostate cancer model, divided into 3 groups: PBS (G1), IR780@LA LNPs + Laser (G2), IR780@TVA LNPs + Laser (G3). (B,C) Flow cytometry results showing the percentage of CD8^+^ T cells (CD3^+^/CD8^+^) and activated T cells (GZMB^+^/CD8^+^) in tumors on day 2 (*n* = 3). (D,E) Flow cytometry results of the percentage of CD8^+^ T cells and GZMB^+^/CD8^+^ T cells in the TDLNs of mice on day 10 (*n* = 3). (F,G) Relative quantification of the flow cytometry results of Figure 6B,C (Data represent: mean ± SD, *n* = 3). (H,I) Relative quantification of the flow cytometry results of Figure 6D,E (Data represent: mean ± SD, *n* = 3). (J) Relative quantification by flow cytometry of the percentage of IFN‐γ^+^/CD8^+^ T cells in the tumors on day 2 (Data represent: mean ± SD, *n* = 3). (K) Volcano plot of upregulated genes (red) and downregulated genes (blue) after treatment with IR780@TVA LNPs + Laser. (L) Heatmap of differentially expressed genes after treatment with IR780@TVA LNPs + Laser. (M) GO enrichment analysis showing significantly enriched immune‐related pathways (blue) and T cell‐related pathways (red) (*n* = 3). ns: no significant difference, *p < 0.05, **p < 0.01, ***p < 0.001, ****p < 0.0001 as determined by one‐way ANOVA followed by Tukey's multiple comparison (F, G, H, I, J).

Given the robust immune response elicited by IR780@TVA LNPs in the immunologically “hot” B16F10 melanoma model, we were interested in determining whether it could be equally effective in immunologically “cold” tumors. Prostate cancer, recognized as a cold tumor, is typically characterized by a deficiency of intratumoral CD8^+^ T cells, with the infiltrating CD8^+^ T cells often exhibiting signs of exhaustion [34, 35]. As shown in Figure 6A, we established a mouse RM‐1 prostate cancer model and randomly divided the mice into three groups: G1 (PBS), G2 (IR780@LA LNPs + laser), and G3 (IR780@TVA LNPs + laser). We isolated the mouse tumors 24 h post‐treatment for flow cytometry analysis. As illustrated in Figure 6B,F, the CD8^+^ T cells in group G2 were 1.21 times higher than those in group G1, indicating that PTT can promote the intratumoral infiltration of CD8^+^ T cells. Furthermore, the CD8^+^ T cells in group G3 increased by 1.55 times compared to group G2, suggesting that the introduction of TVA further stimulated the proliferation of CD8^+^ T cells. More critically, the levels of GZMB^+^/CD8^+^ T cells (20.5%) and IFN‐γ^+^/CD8^+^ T cells (29.1%) in the G3 group were increased by 5.18‐fold and 3.35‐fold compared to the G2 group, respectively, indicating that the TVA released from IR780@TVA LNPs is crucial for maintaining the normal function of CD8^+^ T cells within prostate tumor tissues (Figure 6C,G,J). Subsequently, we performed flow cytometry analysis on the TDLNs after 10 days of treatment. Consistent with the above‐mentioned flow cytometry results of tumor tissues, the G3 group exhibited the highest levels of CD8^+^ T cells, granzyme B‐positive CD8^+^ T cells, and IFN‐γ‐positive CD8^+^ T cells (Figure 6D,E,H,I and Figure S23). These results indicate that IR780@TVA LNPs can also reprogram CD8^+^ T cells in immunologically cold tumors, thereby eliciting a robust immune response.

Additionally, we collected tumor tissues 1 day post‐treatment for transcriptome sequencing (RNA‐seq) analysis. The volcano plot revealed a total of 139 differentially expressed genes between the G3 and G1 groups, with 123 genes upregulated and 16 genes downregulated (Figure 6K). As shown in Figure 6L, genes such as Ccl9, Ccl3, GZMB, ICOS, and IFN‐G were significantly upregulated. It has been reported that Ccl9 and Ccl3 enhance inflammatory responses by promoting immune cell infiltration, proliferation, and activation [36, 37]. Meanwhile, GZMB and IFN‐G are highly associated with T cell activation status, and ICOS is also crucial for enhancing T cell activation and memory formation [38, 39]. Furthermore, Gene Ontology (GO) analysis revealed that these differentially expressed genes are primarily involved in immune system process, inflammatory/immune response, cell surface receptor signaling pathway, and cytokine‐mediated signaling pathways (Figure 6M). In addition, we observed significant enrichment of T cell‐related pathways after treatment with IR780@TVA LNPs + laser, including T cell activation, T cell migration, and the Granzyme B localization to T cell secretory granule, as well as the positive regulation of gamma‐delta T cell activation. These results further validate our earlier conclusion, indicating that treatment with IR780@TVA LNPs not only activates the immune response by triggering PTT but also effectively reprograms CD8^+^ T cells through the release of TVA, thereby bolstering CD8^+^ T cell‐mediated anti‐tumor immunity.

### 2.10 IR780@TVA LNPs Enhance the Efficacy of PD‐L1 Blockade Therapy for Immunologically “Cold” Prostate Cancer

**FIGURE 7:**
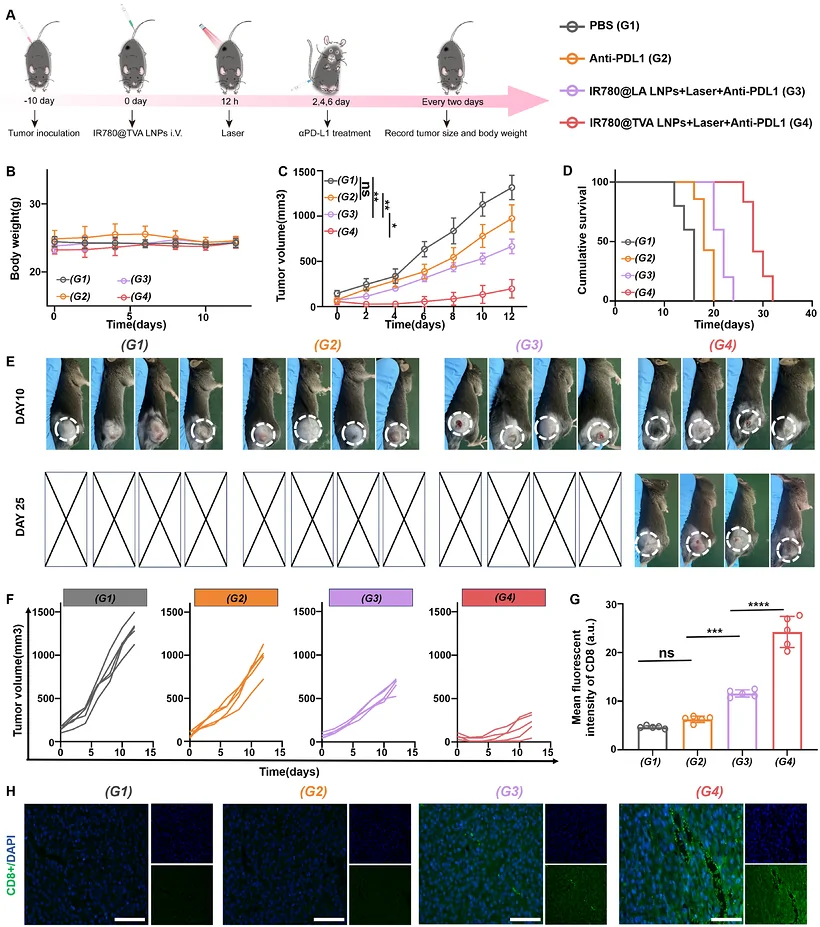
IR780@TVA LNPs combined with anti‐PD‐L1 demonstrate enhanced anti‐tumor effects in an immunologically “cold” prostate cancer model. (A) Schematic diagram of the treatment regimen for the RM‐1 prostate cancer model in mice, divided into four groups: PBS (G1), Anti‐PD‐L1 (G2), IR780@LA LNPs + Laser + anti‐PD‐L1 (G3), and IR780@TVA LNPs + Laser + anti‐PD‐L1 (G4) (*n* = 5). (B) Changes in body weight of mice after different treatment (Data represent: mean ± SD, *n* = 5). (C) The average tumor growth curves after different treatment (Data represent: mean ± SD, *n* = 5). (D) The survival curves of mice after different treatment (*n* = 5). (E) Representative photos of four mice per group on day 10 and day 25 post‐treatment (*n* = 5). (F) Individual tumor growth curves after different treatment (*n* = 5). (G,H) Immunofluorescence images of CD8^+^ T cells and relative quantification of the fluorescence intensity within tumors on day 12 after different treatments (Data represent: mean ± SD, *n* = 5, scale bar: 50 µm). ns: no significant difference, *p < 0.05, **p < 0.01, ***p < 0.001, ****p < 0.0001 as determined by one‑way ANOVA followed by Tukey's multiple comparison (C) or one‐way ANOVA followed by Tukey's multiple comparison (G).

Since IR780@TVA LNPs can effectively induce the transformation of prostate cancer from an immune‐cold tumor to a hot tumor characterized by a substantial presence of CD8^+^ T cells, we hypothesize that IR780@TVA LNPs can improve the therapeutic efficacy of PD‐L1 blockade therapy in prostate cancer. We established an RM‐1 mouse prostate cancer model and randomly divided the tumor‐bearing mice into four groups: (G1) PBS, (G2) anti‐PD‐L1, (G3) IR780@LA LNPs + Laser + anti‐PD‐L1, and (G4) IR780@TVA LNPs + Laser + anti‐PD‐L1 (Figure 7A). Throughout the experimental treatment period, the body weight of mice in each group remained stable (Figure 7B), indicating that the treatment regimen has a favorable safety profile.

As shown in Figure 7C,F, the anti‐PD‐L1 group (G2) did not exhibit significant therapeutic effects compared to the PBS group (G1). In contrast, the IR780@LA LNPs + Laser + anti‐PD‐L1 group (G3) demonstrated a certain degree of tumor growth inhibition following treatment. As anticipated, the IR780@TVA LNPs + Laser + anti‐PD‐L1 group (G4) exhibited the most effective tumor suppression and prolonged the survival of the mice (Figure 7D,E). Meanwhile, H&E images showed that the G4 group manifested high tumor cell necrosis (Figure S24). We isolated tumor tissues from the mice and analyzed the levels of CD8^+^ T cells within the tumors using immunofluorescence staining. The results indicated that the CD8^+^ T cell content in the G4 group increased by 5.27‐fold and 2.11‐fold compared to the control group and the G3 group, respectively (Figure 7G,H). This finding aligns closely with our previous experimental results, suggesting that IR780@TVA LNPs can effectively promote the proliferation of CD8^+^ T cells in prostate cancer tissues, thereby enhancing the response rate to PDL1 blockade therapy [40].

### 2.11 IR780@TVA LNPs Potently Suppress Tumor Growth in a Challenging Large‐Tumor Model

**FIGURE 8:**
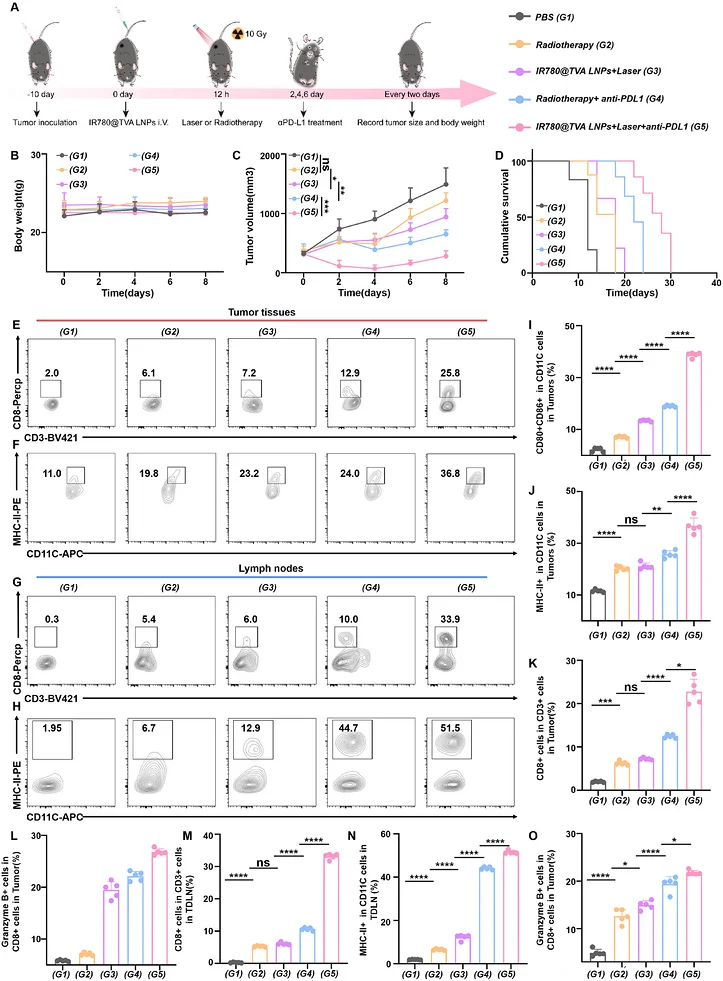
IR780@TVA LNPs potently suppress tumor growth in RM‐1 large‐tumor model. (A) Schematic diagram of the treatment regimen for the RM‐1 large‐tumor model in mice, divided into Five groups: PBS (G1), Radiotherapy (G2), IR780@TVA LNPs + Laser (G3), Radiotherapy + anti‐PD‐L1 (G4), and IR780@TVA LNPs + Laser + anti‐PD‐L1 (G5) (*n* = 5). (B) Changes in body weight of mice after different treatment (Data represent: mean ± SD, *n* = 5). (C) The average tumor growth curves after different treatment (Data represent: mean ± SD, *n* = 5). (D) The survival curves of mice after different treatment (*n* = 5). (E,F) Flow cytometry results showing the percentage of CD8^+^ T cells (CD3^+^/CD8^+^) and MHC‐II (MHC^+^/CD11C^+^) in tumors on day 10 (*n* = 5). (G,H) Flow cytometry results showing the percentage of CD8^+^ T cells (CD3^+^/CD8^+^) and MHC‐II (MHC^+^/CD11C^+^) in TDLNs on day 10 (*n* = 5). (I) Relative quantification of flow cytometry analysis for the CD80^+^CD86^+^ cells in CD11C^+^ cells in tumors of mice on day 10 (Data represent: mean ± SD, *n* = 5). (J) Flow cytometry results of the percentage of MHC‐II^+^/CD11C^+^ cells in tumors of mice on day 10 (Data represent: mean ± SD, *n* = 5). (K) Relative quantification of flow cytometry analysis for the CD8^+^ T cells in tumors of mice on day 10 (Data represent: mean ± SD, *n* = 5). (L) Flow cytometry results of the percentage of GZMB^+^/CD8^+^ T cells in tumors of mice on day 10 (Data represent: mean ± SD, *n* = 5). (M) Relative quantification of flow cytometry analysis for the CD8^+^ T cells in TDLNs of mice on day 10 (Data represent: mean ± SD, *n* = 5). (N) Flow cytometry results of the percentage of MHC‐II^+^/CD11C^+^ cells in TDLNs of mice on day 10 (Data represent: mean ± SD, *n* = 5). (O) Flow cytometry results of the percentage of GZMB^+^/CD8^+^ T cells in TDLNs of mice on day 10 (Data represent: mean ± SD, *n* = 5). ns: no significant difference, *p < 0.05, **p < 0.01, ***p < 0.001, ****p < 0.0001 as determined by one‑way ANOVA followed by Tukey's multiple comparison (C) or one‐way ANOVA followed by Tukey's multiple comparison (I, J, K, L, M, N, O).

Encouraged by the aforementioned experimental results, we further established a large‐volume (≈300 mm^3^) RM‐1 tumor model, aiming to rigorously evaluate our therapeutic strategy under clinically relevant and challenging conditions. This model is characterized by rapid growth and a highly immunosuppressive TME, thereby serving as a stringent testbed for PD‐L1 blockade‐combination regimens. As a control, we selected the standard first‐line treatment regimen for clinically advanced tumors, which consists of radiotherapy combined with PD‐L1 blockade. The tumor‐bearing mice were randomly allocated into five treatment groups: (G1) PBS, (G2) Radiotherapy, (G3) IR780@TVA LNPs + Laser, (G4) Radiotherapy + anti‐PD‐L1, and (G5) IR780@TVA LNPs + Laser + anti‐PD‐L1 (Figure 8A). Notably, all treated mice maintained stable body weights throughout the therapeutic course (Figure 8B), indicating a favorable safety profile. As shown in Figure 8C, compared to the PBS group (G1), radiotherapy alone (G2) elicited a modest tumor growth inhibition. Treatment with IR780@TVA LNPs + Laser (G3) resulted in a more pronounced suppression compared to G2. A stronger anti‐tumor effect was observed in the RT + anti‐PD‐L1 group (G4). Notably, the most potent tumor suppression was achieved in the combination group (G5) receiving IR780@TVA LNPs + Laser + anti‐PD‐L1, which also led to a significant extension of mouse survival (Figure 8D). We subsequently profiled the immune cell phenotypes within tumors 10 days post‐treatment. Flow cytometry analysis revealed that both radiotherapy alone (G2) and IR780@TVA LNPs with laser (G3) elevated the infiltration of CD8^+^ T cells compared to the PBS control (G1) (Figure 8E). Furthermore, the G3 treatment demonstrated a superior capacity to enhance dendritic cell (DC) maturation (18.7%) and MHC‐II expression (23.2%), underscoring the critical role of antigen presentation in initiating adaptive immunity. As anticipated, our combination regimen (G5, IR780@TVA LNPs + Laser + anti‐PD‐L1) induced the most robust immune activation, achieving the highest levels of DC maturation (39.4%) and MHC‐II expression (36.8%) (Figure 8F,I,J).

Notably, the G5 group achieved the most potent tumor suppression. This was accompanied by a significant increase in both the proportion of tumor‐infiltrating CD8^+^ T cells and their functional potency (Figure 8K,L), as evidenced by the highest levels of GZMB^+^/CD8^+^ T cells (4.82‐fold than that of the control group). Consistent with these intratumoral findings, flow cytometric analysis of tumor‐draining lymph nodes (TDLNs) revealed a parallel trend. The G5 group exhibited the most pronounced DC maturation and MHC‐II expression, which were 6.96‐fold and 29.3‐fold higher than that in the control group, respectively (Figure S25 and Figure 8H,N). Correspondingly, the levels of CD8^+^ T cells and cytotoxic GZMB^+^/CD8^+^ T cells in TDLNs were also the highest among all groups (Figure 8G,M,O). Collectively, these findings confirm that IR780@TVA LNPs combined with anti‐PD‐L1 exhibit superior anti‐tumor efficacy and immune activation capability compared to first‐line clinical therapies, even in advanced, highly immunosuppressive RM‐1 large tumor models.

### 2.12 IR780@TVA LNPs + Anti‐PD‐L1 Abrogates Lung Metastasis by Stimulating Systemic Anti‐Tumor Immunity

**FIGURE 9:**
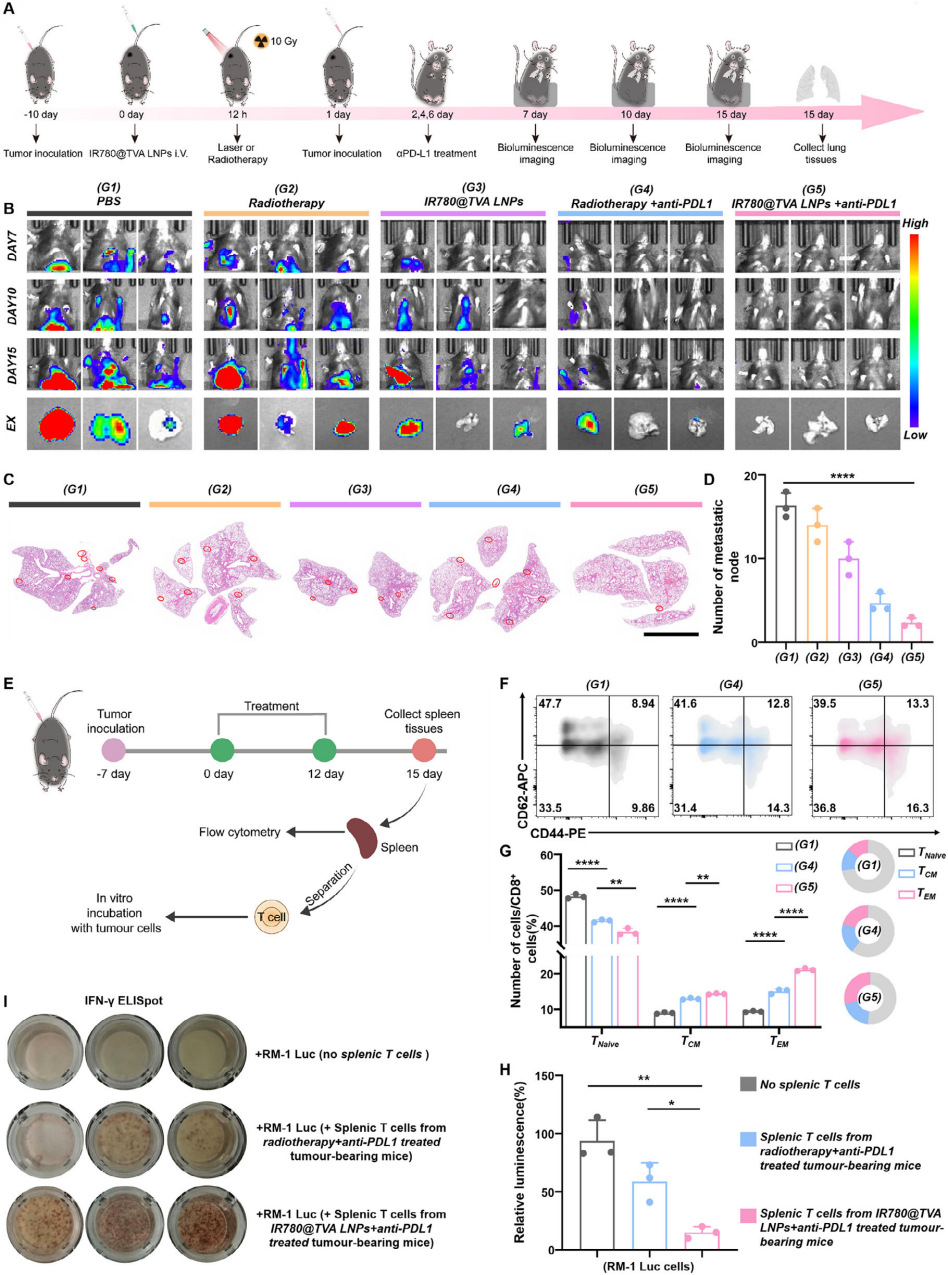
IR780@TVA LNPs + laser + anti‐PD‐L1 abrogates lung metastasis by stimulating systemic anti‐tumor immunity. (A) Schematic of the treatment schedule in a mouse RM‐1 lung metastasis model. Mice were divided into five groups (*n* = 5): PBS (G1), radiotherapy (G2), IR780@TVA LNPs + Laser (G3), radiotherapy + anti‐PD‐L1 (G4), and IR780@TVA LNPs + Laser + anti‐PD‐L1 (G5). (B) In vivo and ex vivo bioluminescence images of lung tissues (representative three mice in each group). (C) H&E staining image of lung tissue. The metastatic lesions were indicated with red circles (scale bar: 1 mm). (D) Quantification of lung metastatic nodules of mice in different groups (Data represent: mean ± SD, *n* = 3). (E) Schematic of the experimental design for evaluating the systemic tumor‐specific immunomemory response. (F) T cells isolated from the spleens of mice receiving different treatments were co‐incubated with RM1‐Luc cells for 24 h, and RM1‐Luc cell viability was determined by measuring fluorescence after the addition of sodium fluorescein (*n* = 3). (G) Representative flow cytometry results of *T*_Naive_ (CD3^+^CD8^+^/CD44^−^/CD62L^+^), *T*_CM_ (CD3^+^CD8^+^/CD44^+^/CD62L^+^), and *T*_EM_ (CD3^+^CD8^+^/CD44^+^/CD62L^−^) in mouse spleen tissues after different treatments (Data represent: mean ± SD, *n* = 3). (H) Viability of RM‐1‐Luc cells after 24 h co‐incubation with T cells isolated from mouse spleens (Data represent: mean ± SD, *n* = 3). (I) Analysis of IFN‐γ‐secreting activated T cells by ELISpot assay (*n* = 3). ns: no significant difference, *p < 0.05, **p < 0.01, ***p < 0.001, ****p < 0.0001 as determined by one‐way ANOVA followed by Tukey's multiple comparison (D, G, H).

The induction of a durable, systemic immune response capable of preventing tumor recurrence is a pivotal goal in cancer immunotherapy. Having previously demonstrated that the combination of IR780@TVA LNPs and anti‐PD‐L1 can induce immunogenic memory in a melanoma model, we further evaluated its potential to control systemic metastasis in the RM‐1 advanced tumor model. Tumor‐bearing mice (tumor volume > 300 mm^3^) were randomized into five groups: (G1) PBS, (G2) Radiotherapy, (G3) IR780@TVA LNPs + Laser, (G4) Radiotherapy + anti‐PD‐L1, and (G5) IR780@TVA LNPs + Laser + anti‐PD‐L1. One day post‐irradiation, the mice were received an intravenous injection of 5 × 10^5^ Luc‐RM1 cells to simulate hematogenous metastasis, and metastatic progression was monitored via bioluminescent imaging (Figure 9A). The results indicate that on the seventh day, the use of RT (G2) alone was insufficient to control distant metastasis; however, the G3 regimen alleviated lung metastasis. Nevertheless, over time, the fluorescent signal in the lungs significantly intensified. While G4 (RT + anti‐PD‐L1) exhibited better efficacy, some mice still experienced lung metastasis. Notably, the G5 group (IR780@TVA LNPs + Laser + anti‐PD‐L1) demonstrated the most potent abrogation of pulmonary metastasis, with no detectable bioluminescent signal throughout the monitoring period. Ex vivo analysis of lung tissues collected at the endpoint correlated with these findings, showing a significant reduction in visible metastatic nodules in the G5 group, which was further confirmed by histopathological examination (Figure 9B,C,D).

To decipher the mechanism underlying this potent systemic protection, we analyzed the induction of immunological memory (Figure 9E). Flow cytometric analysis of splenocytes revealed that the G5 group significantly expanded both effector memory T (*T*_EM_, CD3^+^CD8^+^CD44^+^CD62L^−^) and central memory T (*T*_CM_, CD3^+^CD8^+^CD44^+^CD62L^+^) cell populations compared to the control and G4 group, and concomitant with a reduction in naïve T cells (*T*_Naive_, CD3^+^CD8^+^CD44^−^CD62L^+^) (Figure 9F,G). In addition, splenic T cells isolated from G5‐treated mice exhibited superior ex vivo cytotoxic activity against RM‐1‐Luc target cells and generated a significantly higher number of IFN‐γ spots upon antigen‐specific recall in an ELISpot assay, compared to those from other groups (Figure 9H,I and Figure S26). In summary, these data provide compelling evidence that IR780@TVA LNPs + anti‐PD‐L1 not only eradicates primary tumors but also elicits a robust and systemic anti‐tumor immune response, thereby inhibiting metastatic dissemination.

## 3 Conclusion

Given the pivotal role of CD8^+^ T cells in adaptive anti‐tumor immune responses, we propose a NNM strategy for the remotely controlled release of TVA via NIR to activate tumor‐infiltrating CD8^+^ T cells. As proof of concept, we prepared a fatty acid eutectic mixture using TVA and SA, ingeniously leveraging TVA itself as a gated phase‐change material, which served as the organic phase‐change material to encapsulate IR780, forming NIR‐responsive lipid nanoparticles IR780@TVA LNPs. IR780@TVA LNPs can target tumor tissues and induce PTT, thereby initiating adaptive anti‐tumor immunity. Simultaneously, as the temperature exceeds the melting point of the eutectic mixture, TVA is released within tumors and reprograms CD8^+^ T cells through cAMP‐PKA‐CREB axis. Notably, IR780@TVA LNPs can increase the concentration of TVA in tumor tissues by 21.86 times (before radiation) or 8.89 times (2 h after radiation) compared to direct oral administration of free TVA. IR780@TVA LNPs can upregulate PD‐L1 on the tumor and downregulate PD‐1 on the CD8^+^ T cells, enhance the levels of CD8^+^ T cells in both “hot” and “cold” tumors, reverse T cell exhaustion and improve T cell function, thereby enhancing the therapeutic efficacy of PD‐L1 blockade therapy. Furthermore, we have validated that IR780@TVA LNPs establish effective immune memory in cured mice, enabling them to reject tumor rechallenge and reduce recurrence. Although the sample size in the rechallenge experiment was limited (*n* = 4) due to the complete remission rate, the immune protection was further supported by ex vivo cytotoxicity and IFN‐γ ELISpot assays in the RM‐1 prostate cancer model, demonstrating tumor antigen‐specific recall responses. Collectively, these data confirm that our NNM strategy can induce systemic, antigen‐specific memory T cell immunity. Notably, our strategy was also effective in advanced large tumor models and demonstrated superiority over first‐line clinical therapies.

This work pioneers the novel concept of “Nano‐Nutritional Medicine, NNM” to reprogram tumor immunosuppressive microenvironment, establishing the first platform for the targeted delivery and on‐demand release of dietary immunonutrients via nanotechnology. This strategy not only avoids the potential risk of immune‐related adverse reactions associated with exposing the entire body to high doses of TVA, but also ensures a sufficient real‐time supply of TVA during the critical period of T cell activation following PTT. We acknowledge several limitations of the current study. It should be noted that while this study demonstrates the effectiveness of IR780@TVA LNPs in combating cold tumors represented by prostate cancer, their efficacy in other more stringent and complex immunosuppressive tumor models (such as pancreatic ductal adenocarcinoma rich in tumor stroma) still requires further investigation in the future. Moreover, the penetration depth of 808 nm NIR light restricts the direct application of this IR780‐based platform to deep‐seated or orthotopic tumors. Interventional fiber‐optic delivery or alternative triggering modalities (e.g., magnetic hyperthermia nanoparticles) could be considered to address this issue, but experimental validation in more clinically relevant deep tumor models is still warranted. In addition, although the bystander effect model is strongly supported by our data, direct visualization of TVA release from nanoparticle‐laden cells and its diffusion to T cells has not been performed. To solve this problem, single‐cell imaging or spatial metabolomics would be valuable in future studies. Finally, the long‐term safety of high‐dose TVA delivered via nanoparticles has not been evaluated. Although TVA is a natural dietary component, GMP‐grade production and rigorous toxicological studies are required before clinical translation.

This nano‐therapeutic platform, composed of natural food ingredients and FDA‐approved biocompatible polymers, demonstrates significant potential for clinical translation. However, prior to this, we acknowledge that TVA requires GMP‐grade production and clinical safety validation. While dedicated processes are under development, microbial biotransformation presents a viable scalable route. Although systematic human trials for high‐dose TVA are lacking, its long history as a dietary component and distinction from harmful industrial trans‐fats provide a favorable safety foundation. Its future clinical application may align with a “drug repurposing” paradigm for bioactive nutrients, supported by precedents like prescription omega‐3 formulations. Importantly, our nano‐delivery platform, which enables tumor‐localized release, further mitigates systemic exposure risks. These considerations underscore the feasible and de‐risked translational pathway of our TVA‐based NNM strategy. Last but not least, the introduction of this NNM concept also paves the way for the application of other nutrients in cancer immunotherapy. Moreover, the concept of using nanotechnology to deliver and remotely activate immunonutrients can be extended to other bioactive food components, potentially opening a new paradigm in cancer immunotherapy. Future directions include optimizing the nanoparticle composition for deeper tissue penetration (e.g., using second‐window NIR‐II dyes or nanoparticles with magnetocaloric effect), evaluating the platform in patient‐derived xenograft (PDX) and orthotopic models, and exploring combination with other immunomodulatory nutrients.

## 4 Experimental Section

### 4.1 Materials

*Trans*‐vaccenic acid (TVA, 99%), stearic acid (98.0%), lauric acid (99.5%), 1, 2‐distearoyl‐sn‐glycero‐3‐phosphoethanolamine‐*N*‐[methoxy(polyethylene glycol)‐5000] (DSPE‐PEG5000, *M*_w_ = 5000), soybean lecithin (98.0%), IR780 iodide (IR780, 98%), carboxyfluorescein succinimidyl ester (CFSE), and methyl thiazolyl tetrazolium (MTT) were purchased from Sigma‐Aldrich. Anti‐PD‐1/PD‐L1 antibodies were obtained from BioXcell. Penicillin‐streptomycin, red blood cell lysis buffer, ECL ultrasensitive kit (ED 0015‐B), Cell Counting Kit‐8 (CCK‐8), bicinchoninic acid (BCA) protein assay kit, prestained protein molecular weight marker (EC 0019), and 10% sodium dodecyl sulfate‐polyacrylamide gel electrophoresis (SDS‐PAGE) preparation kit (EC 0023) were supplied by Sparkjade Biotechnology Co., Ltd. (China). Fetal bovine serum (FBS) and RPMI‐1640 medium were purchased from BioChannel Biotechnology Co., Ltd. (China). Recombinant murine GM‐CSF (P6006) and 0.25% trypsin‐EDTA were obtained from Beyotime Biotechnology Co., Ltd. (China). The mouse TNF‐α ELISA kit was provided by Jiangsu Jianglai Biotechnology Co., Ltd. (China). Type IV collagenase was acquired from Solarbio Science & Technology Co., Ltd. (China). Purified anti‐mouse CD3 (Cat No. 100202), purified anti‐mouse CD28 (Cat No. 102102), FITC‐conjugated anti‐mouse CD3 (Cat No. 100203), PerCP‐conjugated anti‐mouse CD8 (Cat No. 100731), PE‐conjugated anti‐mouse granzyme B (Cat No. 372215), APC‐conjugated anti‐mouse IFN‐γ (Cat No. 505810), APC‐conjugated anti‐mouse CD11c, FITC‐conjugated anti‐mouse CD80, PE‐conjugated anti‐mouse CD86, PE‐conjugated anti‐mouse TOX (Cat No. 12‐6502‐80), PE‐conjugated anti‐mouse CD44 (Cat No. 103007), and APC‐conjugated anti‐mouse CD62L (Cat No. 104412) were purchased from BioLegend (USA).

### 4.2 Synthesis of IR780@TVA LNPs

1. The thermal properties of the eutectic mixture of TVA, lauric acid (LA), and stearic acid at different weight ratios were evaluated using differential scanning calorimetry (DSC). The samples (3 mg) were heated at a rate of 10°C per minute in a nitrogen atmosphere, with a purge flow rate of 50 mL/min.
2. Lipid nanoparticles were fabricated using the nanoprecipitation method. In brief, TVA and stearic acid (in a weight ratio of 9:1), as well as LA and stearic acid (in a weight ratio of 4:1, marked as control group), were initially dissolved in methanol at a concentration of 4 mg/mL. Lecithin and DSPE‐PEG5000 (in a weight ratio of 3.5:1) were dissolved in 2 mL of a 4% ethanol aqueous solution. The phospholipid solution (2 mL) was then heated to 50°C. Subsequently, 0.6 mL of the solubilized fatty acid solution containing IR780 (1 mg/mL) was added dropwise to the preheated phospholipid solution and stirred for 10 min, followed by vigorous vortexing for 2 min. The mixture was then transferred to ice water and cooled for 2 min, after which the turbid solution was returned to ambient temperature and vortexed for another 2 min. Then the solution was filtered through a 0.2 µm surfactant‐free cellulose acetate membrane (Thermo Fisher Scientific). To remove unencapsulated molecules and organic solvents, a Vivaspin 6 centrifugal concentrator (Sartorius, MWCO = 10 kDa) was used. The final lipid nanoparticles, IR780@TVA LNPs and IR780@LA LNPs (control group), were stored at 4°C for further experimental use.

### 4.3 Characterization of IR780@TVA LNPs

The particle size distribution and zeta potential were measured at 25°C using a Nano ZS (Malvern Instruments, UK). The morphology of the lipid nanoparticles was observed via transmission electron microscopy (TEM) at 80 kV after negative staining with 1.0% uranyl acetate. The drug loading of IR780 was quantified by measuring the UV/VIS spectra of lipid nanoparticles dissolved in methanol, followed by the application of the standard curve method (absorption wavelength: 780 nm).

The drug loading of TVA was quantified using gas chromatography methods after methyl esterification. Briefly, 500 µL of 2% sulfuric acid‐methanol solution was added to each sample and incubated at 50°C for 2 h. Subsequently, 100 µL of saturated saline solution (NaCl in HPLC‐grade water) and 500 µL of HPLC‐grade n‐hexane were added, followed by vortex mixing. The upper n‐hexane layer was collected and evaporated under nitrogen. Each sample was reconstituted in 100 µL fresh HPLC‐grade n‐hexane, vortexed, and transferred to GC vials. Quantification was performed by gas chromatography (GC; Agilent 7890B) equipped with an HP‐5MS UI capillary column (30 m × 0.25 mm ID, 0.25 µm film thickness). Fatty acid methyl esters (FAMEs) were separated using a temperature‐programmed method with the following conditions: injector temperature was set at 270°C, flame ionization detector was set at 280°C, the carrier gas was nitrogen at a flow of 1.5 mL/min, with a split ratio of 1:10. The oven temperature was initially held at 160°C for 3 min, ramped at 10°C/min to 190°C (held for 5 min), and further increased at 10°C/min to 240°C (held for 5 min). TVA‐FAME standards were used for peak identification.

The encapsulation efficiency (EE) was calculated as follows: EE = (weight of the drug in nanoparticles)/(weight of the added drug) × 100%.

The loading capacity (LC) was determined using the formula: LC = (weight of the drug in nanoparticles)/(weight of the nanoparticles) × 100%.

### 4.4 Photothermal Effect of IR780@TVA LNPs

The photothermal effect was evaluated by exposing IR780@TVA LNPs and IR780@LA LNPs (1 mL, [IR780] = 0.2 mg/mL in water), free IR780 (1 mL, [IR780] = 0.2 mg/mL in water), and water (1 mL) to an 808 nm near‐infrared (NIR) laser at a power density of 1.0 W/cm^2^ for 5 min. The temperature was recorded at 30 s intervals using a PTi120 thermal imaging camera. After cooling to ambient temperature, two additional irradiation cycles were performed, each lasting 5 min.

## Author Contributions

K.L. and Y.L.L. contributed equally to this work. W.S., H.M.L., and K.L. conceived this work. K.L., Y.L.L., P.Z., S.T., and W.S. designed, prepared, and characterized the materials. K.L., Y.L.L., M.J.W., W.S.Y., Q.W.S., and Z.W.S. carried out the in vitro, ex vivo, and in vivo experiments. K.L., S.T., Y.Y., P.S., D.K.Y., P.Z., and W.S. analyzed the data and discussed the results. W.S., K.L., H.M.L., P.Z., D.K.Y., and P.S. wrote and edited the manuscript. H.M.L., P.S., D.K.Y., and W.S. supervised the project. W.S., P.S., D.K.Y., and H.M.L. secured funding for the research. The manuscript was written through contributions of all authors. All authors have given approval for the final version of the manuscript.

## Conflicts of Interest

The authors declare no conflicts of interest.

## Supporting information



Drug release assay, cytotoxicity assay, detection of IL‐2 levels, identification of immunogenic cell death (ICD), evaluation of the dendritic cells maturation, T cell‐mediated tumor cell killing assay, in vivo distribution experiment, in vivo antitumor efficacy evaluation, Cellular uptake of IR780@TVA LNPs in vitro, Analysis of TVA‐Mediated indirect PD‐L1 upregulation via conditioned T cell supernatants, Analysis of antigen‐specific T cell function via IFN‐γ ELISpot Assay, and Figures S1–S24, please refer to the Supporting Information. **Supporting File**: advs75992‐sup‐0001‐SuppMat.docx.

## Data Availability

All data needed to evaluate the conclusions or reproduce these findings in the paper are present in the paper and/or the Supporting Information.
