# Supplementary material for: Toward Nano‐Nutritional Medicine: A Remotely Activated Trans‐Vaccenic Acid‐Based Lipid Nanoparticles for Enhancing Immune Checkpoint Blockade Therapy
Source: Adv Sci (Weinh). 2026 Jun 9:e75992. Online ahead of print. doi: 10.1002/advs.75992 (PMC13336454; doi:10.1002/advs.75992)
Supplement: Supplementary file 1 — Drug release assay, cytotoxicity assay, detection of IL‐2 levels, identification of immunogenic cell death (ICD), evaluation of the dendritic cells maturation, T cell‐mediated tumor cell killing assay, in vivo distribution experiment, in vivo antitumor efficacy evaluation, Cellular uptake of IR780@TVA LNPs in vitro, Analysis of TVA‐Mediated indirect PD‐L1 upregulation via conditioned T cell supernatants, Analysis of antigen‐specific T cell function via IFN‐γ ELISpot Assay, and Figures S1–S24, please refer to the Supporting Information. Supporting File: advs75992‐sup‐0001‐SuppMat.docx. [file ADVS-9999-e75992-s001.docx]

**Supplementary information**

**Toward Nano-Nutritional Medicine: A remotely activated *trans*-vaccenic acid-based lipid nanoparticles for enhancing immune checkpoint blockade therapy**

Kang Liu^1^**^#^**, Yunlong Li^1^**^#^**, Hanmeng Liu^1^*, Peng Zhang^8^, Mingjing Wang^1^, Quanwei Sun^1^, Wenshuo Yang^1^, Zhengwei Song^1^, Song Tan^1^, Ye Yang^1^, Peng She^3^*, Dengke Yin^1,4,5,6^*, Wei Shen^1,2,4,5,7,8^*

1. School of Pharmacy, Anhui University of Chinese Medicine, Hefei, 230012, China.

2. Key Laboratory of Xin'an Medicine (Anhui University of Chinese Medicine), Hefei, 230012, China.

3.The Seventh Affiliated Hospital of Sun Yat-sen University, Department of Orthopedics, 628 Zhenyuan Road, Guangming district, Shenzhen, 518000, China.

4. Anhui Province Key Laboratory of Pharmaceutical Preparation Technology and Application, Hefei, 230012, China.

5. Engineering Technology Research Center of Modernized Pharmaceutics, Anhui Education Department (AUCM), Hefei, 230012, China.

6. Anhui Provincial Key Laboratory of Chinese Medicinal Formula, Hefei, 230021, China.

7. National Center for Translational Medicine (Shanghai) SHU Branch, Shanghai University, Shanghai, 200444, China.

8. State Key Laboratory of Polymer science and Technology (Changchun Institute of Applied Chemistry Chinese Academy of Sciences), Changchun 130022, PR China.

* Corresponding author:

E-mail addresses: hmliu@ahtcm.edu.cn (H. Liu); [Shep@mail.sysu.edu.cn (P](mailto:Shep@mail.sysu.edu.cn%20(P). She); yindengke@ahtcm.edu.cn (D. Yin); wshen@ahtcm.edu.cn (W. Shen).

**Materials**

**NIR-Triggered drug release assay**

To determine the TVA release profile under NIR irradiation, IR780@TVA LNPs were exposed to an 808 nm NIR laser at a power density of 0.6 W/cm^2^ for 8 minutes. At predetermined time intervals, 50 μL of the irradiated supernatant was collected via low-speed centrifugation (190×g, 4°C, 5 min) for TVA quantification following methyl ester derivatization. And the quantification of TVA was performed by gas chromatography methods as mentioned above.

**Laser irradiation conditions**

For in vivo photothermal treatment, mice were anesthetized with isoflurane and maintained in a fixed prone position to minimize motion during laser exposure. An 808 nm near-infrared laser (model: WIRPD808-5F) was directed at the tumor area at a power density of 1.0 W/cm² for 10 min. The laser fiber was positioned approximately 20 cm above the tumor surface, producing an irradiation spot of about 2 cm in diameter that fully covered the tumor region. Tumor surface temperature was continuously monitored during irradiation using an infrared thermal imaging camera (model: PTi120).

**Phantom study for evaluating thermal penetration and phase-transition activation**

To evaluate whether the applied near-infrared (NIR) irradiation conditions were sufficient to induce thermal activation throughout tumors of varying sizes, calcium alginate hydrogel phantoms, composed of 22.5 mg/mL sodium alginate and 2.5 mg/mL calcium chloride, containing IR780@TVA LNPs were prepared to simulate tumor tissues of different volumes. Briefly, phantoms with sizes approximating 60 mm³ and 350 mm³ tumors were fabricated, and the nanoparticles were uniformly dispersed within the hydrogel matrix. The phantoms were then irradiated under the same laser conditions used in vivo (808 nm, 1.0 W/cm², 10 min, irradiation distance 20 cm). Temperatures at both the peripheral and central regions (the cross section) of the phantoms were monitored by infrared thermal imaging. The recorded temperatures were used to determine whether the internal temperature exceeded the phase-transition temperature of the TVA:SA eutectic mixture (40.5°C), thereby indicating the feasibility of triggering payload release throughout the simulated tumor tissue.

**Cell lines and animals**

All cell lines, including Luc-B16F10 (mouse melanoma cancer cell line expressing firefly luciferase), and RM-1 (mouse prostate cancer cell line) were obtained from the Shanghai Cell Bank of the Chinese Academy of Sciences and cultured in an RPMI-1640 cell culture medium containing 10% fetal bovine serum (FBS) and 1% penicillin-streptomycin. All cell lines were cultured in a humidified 37 °C incubator with 5% CO2. C57BL/6 female mice (eight weeks-old, 22-25 g body weight) C57BL/6 male mice (eight weeks-old, 22-25 g body weight) were purchased from Ziyuan Laboratory Animal Technology Co. Ltd (Hangzhou, China), and maintained in a room at ambient temperature (24 ± 2 °C) with a humidity of 51 ± 5%, and a 12 h light/dark cycle. The mice had ad libitum access to food and water, ensuring animal welfare. All animal experiments were approved by the Ethics Committee of the Anhui University of Chinese Medicine (AHUCM-mouse-2025034).

**Cytotoxicity assay**

1) The cytotoxicity was measured using CCK-8 method. B16F10 cells were seeded at a density of 5 × 10^3^ cells per well in a 96-well plate and cultured for 24 hours. After removing the supernatant, the cells were washed twice with PBS (pH 7.4). The cells were then treated with 80, 40, 20, and 10 μM of TVA, LA, and SA for 24 hours, respectively. Following treatment, the medium in each well was discarded, and the cells were washed with PBS (pH 7.4). Subsequently, 10 μL of CCK-8 working solution and 100 μL of fresh medium were added to each well, and the cells were incubated at 37°C for 1 hour. The cytotoxicity was quantified using a microplate reader (absorbance at 450 nm).

2) B16F10 cells were seeded into 96-well plates at a density of 5 × 10^3^ cells per well and cultured for 24 hours. The supernatant was removed, and the cells were washed twice with PBS (pH 7.4). Free IR780 and IR780-loaded lipid nanoparticles (IR780@TVA LNPs and IR780@LA LNPs) were added to the culture medium at IR780 concentrations of 1.5, 0.75, and 0.5 μM. After a 6-hour incubation, the medium was replaced with fresh culture medium containing 10% fetal bovine serum, and the cells were either irradiated or not irradiated. The irradiation group was exposed to 808 nm at 1.0 W/cm^2^ for 5 minutes, followed by an additional 6-hour incubation. After aspirating the medium from each well, the cells were washed with PBS (pH 7.4), and 10 μL of CCK-8 working solution and 100 μL of fresh medium were added. The cells were then incubated at 37°C for 1 hour, and cytotoxicity was quantified using a microplate reader.

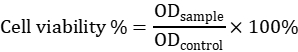


**Detection of IL-2 levels** **secreted by Jurkat T Cells**

Jurkat T cells were cultured in RPMI-1640 medium supplemented with 10% fetal bovine serum and 1% penicillin-streptomycin. The cells were seeded into 6-well plates at a density of 2 × 10^5^ cells per well. The Jurkat T cells were subjected to the following treatments: (Ⅰ) PBS, (Ⅱ) IR780@LA LNPs pretreated with laser irradiation, (Ⅲ) IR780@TVA LNPs, (Ⅳ) IR780@TVA LNPs pretreated with laser irradiation, and (Ⅴ)free TVA . The irradiation samples were exposed to 808 nm light at a power density of 1.0 W/cm^2^ for 5 minutes, followed by co-culture with Jurkat T cells for 48 hours. Subsequently, the cells were activated with 2.5 μg/ml anti-CD3 and 0.5 μg/ml anti-CD28 antibodies for 12 hours, after which the IL-2 levels in the culture supernatant were quantified using the ELISA Kit.

***In vitro* identification of immunogenic cell death (ICD).**

To observe the exposure of calreticulin (CRT), B16F10 cells were seeded at a density of 2 × 10^5^ cells per well in a 6-well plate and cultured for 24 hours. Subsequently, they were incubated for 6 hours with the following treatments: (G1) PBS, (G2) IR780@LA LNPs, (G3) IR780@TVA LNPs, (G4) IR780@LA LNPs + Laser, and (G5) IR780@TVA LNPs + Laser (the concentration of IR780 was set as 1 µg/ml). After incubation, the medium was replaced with fresh culture medium containing 10% fetal bovine serum, and the cells were either irradiated or not. The irradiation group was exposed to 808 nm light at a power density of 1.0 W/cm² for 5 minutes, followed by an additional incubation period of 6 hours. The cells were then washed with PBS and fixed with 4% paraformaldehyde at room temperature for 20 minutes. After wash with PBS, the cells were blocked with 1% BSA at room temperature for 2 hours. Finally, B16F10 cells were incubated with anti-CRT antibody (1:100 dilution in flow cytometry buffer) at 37°C for 2 hours, followed by incubation with AF647-conjugated secondary antibody (1:500 dilution in flow cytometry buffer) at 37°C in the dark for 1 hour. The cells were then analyzed using flow cytometry.

**Cellular uptake of** **IR780@TVA LNPs *in vitro***

To elucidate the primary endocytic pathways through which our nanoparticles enter different cell types within the tumor microenvironment, a systematic pharmacological inhibition assay was conducted. Ce6-labeled nanoparticles (Ce6@TVA LNPs) were first prepared by incorporating the fluorescent dye Ce6 into the lipid nanoparticle formulation. Two key cell types, RAW264.7 macrophages and RM-1 tumor cells, were seeded in 24-well plates at a density of 2 × 10⁵ cells per well and cultured for 24 hours. Cells were then pretreated for 1 hour with specific inhibitors targeting distinct internalization routes: chlorpromazine (CPZ, 30 μM) for clathrin-mediated endocytosis, methyl-β-cyclodextrin (M-βCD, 3 μM) for caveolae-mediated endocytosis, 5-(N-ethyl-N-isopropyl) amiloride (EIPA, 50 μM) for macropinocytosis, and cytochalasin D (20 nM) for phagocytosis/actin polymerization. Following inhibitor pretreatment, Ce6@TVA LNPs (equivalent to 1 μg/mL Ce6) were added to the cells and incubated for 6 hours. Cells were then washed, harvested, and analyzed by flow cytometry to quantify the fluorescence intensity of internalized Ce6, thereby determining the relative contribution of each pathway to nanoparticle uptake in macrophages versus tumor cells.

**Evaluation of the dendritic cells maturation *in vitro***

Bone marrow-derived dendritic cells (BMDCs) were obtained from C57BL/6 mice, followed by culture and differentiation. B16F10 cells were seeded in 6-well plates at a density of 1×10^5^ cells per well and incubated with (G1) PBS, (G2) IR780@LA LNPs, (G3) IR780@TVA LNPs, (G4) IR780@LA LNPs + Laser, and (G5) IR780@TVA LNPs + Laser (the concentration of IR780 was set as 1 μg/ml) for 6 hours. Subsequently, 1×10^5^ BMDCs suspended in fresh medium were added to each well for co-culture. After 24 hours, the BMDCs were collected, stained with anti-CD11c-APC, anti-CD80-FITC, and anti-CD86-PE anti-mouse antibodies, and then were analyzed using flow cytometry.

**T cell‐mediated tumor cell killing assay *in vitro***

1) Mouse primary CD8^+^ T cells were isolated from the spleen of C57BL/6 mice by magnetic bead purification using EasySep Mouse CD8^+^ T Cell Isolation Kit according to the manufacturer’s instructions (Stemcell Technologies). Isolated primary CD8^+^ T cells were activated in vitro for 24 h with plate-bound anti-CD3 (10 μg ml^−1^; Biolegend) and anti-CD28 (5 μg ml^−1^; Biolegend) antibodies in Click’s medium at 37 °C and 5% CO_2_ incubator.

2) B16F10 cells were seeded into a well plate at a density of 5×10^4^ cells per well and incubated overnight. The B16F10 cells were treated with (a) PBS, (b) IR780@LA LNPs + Laser, (c) IR780@TVA LNPs + Laser, and (d) IR780@LA LNPs + Laser + Free TVA for 6 hours, followed by co-culturing with 1×10^5^ BMDCs suspended in fresh medium per well. After 24 hours, the BMDCs were collected and co-cultured with murine CD8^+^ T cells at a BMDC/T cell ratio of 1:4 for three days. Next, the T cells were collected and co-cultured with B16F10 cells, and the fresh medium containing (a) PBS, (b) IR780@LA LNPs pretreated with laser irradiation, (c) IR780@TVA LNPs pretreated with laser irradiation, and (d) Free TVA were added, respectively. After 24 hours of co-culture, the apoptosis of tumor cells was detected using flow cytometry.

**Analysis of antigen-specific T cell function via IFN-γ ELISpot Assay**

To evaluate the functional capacity of T cells and their antigen-specific recall response following different treatments, an IFN-γ ELISpot assay was performed. Splenocytes were aseptically harvested from mice in the naïve, radiotherapy + αPD-L1, and IR780@TVA LNPs + αPD-L1 treatment groups. Single-cell suspensions were prepared by mechanical dissociation through a cell strainer, followed by red blood cell lysis. Total splenocytes were then stimulated in vitro with anti-CD3/CD28 antibodies and IL-2 for 24 hours to activate T cells. Meanwhile, RM-1 tumor cells were seeded in an ELISpot plate at a density of 5 × 10³ cells per well and allowed to adhere for 24 hours. Subsequently, the pre-activated splenocytes were added to the tumor cell-coated wells and co-cultured for an additional 24 hours. After the co-culture period, the plate was developed according to the manufacturer’s protocol to detect spots representing IFN-γ secretion. The number of spots per well was quantified to assess the frequency of tumor-reactive, IFN-γ-producing T cells in each treatment group.

***In vivo* distribution experiment**

1) Firstly, we utilized the NIR fluorescent dye IR780 to evaluate the *in vivo* retention of the lipid nanoparticles. A total of 1×10^6^ B16F10 cells were subcutaneously injected into the right dorsal flank of C57BL/6 mice (6-8 weeks old). When the average tumor size reached approximately 100 mm³, IR780@TVA LNPs and IR780@LA LNPs were administered via tail vein injection. Subsequently, the mice were imaged at predetermined time points (λex=745 nm, λem=800 nm) using the IVIS spectral imaging system. The major organs (heart, liver, spleen, lungs, and kidneys) and tumor tissues were collected within 5 minutes post-euthanasia for ex vivo imaging using an in vivo imaging system (IVIS, PerkinElmer) with excitation and emission wavelengths of 745 nm and 800 nm, respectively. Subsequently, the tumor tissues were stored at −80°C for subsequent extraction and analysis of intratumoral TVA.

2) Briefly, tumor tissues were fragmented into small pieces weighing 10-40 mg. Extraction was performed using chilled HPLC-grade methanol, water, and chloroform in a volume ratio of 600:400:300. Samples were vortexed for 15 min at 4°C, followed by centrifugation at 21,000 ×g for 10 min (4°C) to separate the top aqueous layer and bottom organic layer. Both layers were collected, dried under nitrogen gas, and stored at −80°C for subsequent GC analysis. And the quantification of TVA was performed by gas chromatography methods as mentioned above.^[1]^

**In vivo antitumor efficacy evaluation**

1. Initially, 1×10^6^ luciferase-labeled B16F10 (B16F10-LUC) cells were injected into the right flank of female C57BL/6 mice to establish a melanoma model. When the tumor volume reached approximately 60 mm³, the mice were randomly divided into seven groups. On day 0, the mice were treated with 100 μl of G1: PBS (i.v.), G2: IR780@TVA LNPs (i.v., IR780: 4 mg/kg), G3: IR780@LA LNPs (i.v., IR780: 4 mg/kg), G4: TVA (p.o., TVA: 12 mg/kg), G5: IR780@LA LNPs (i.v., IR780: 4 mg/kg) + Laser, G6: IR780@LA LNPs (i.v.) + Laser + TVA (p.o.) (IR780: 4 mg/kg, TVA: 12 mg/kg), or G7: IR780@TVA LNPs (i.v.) + Laser (IR780: 4 mg/kg, TVA: 12 mg/kg), followed by laser irradiation after 12 hours (i.v., intravenous injection; p.o., oral administration), irradiation was performed at 808 nm with a power density of 1.0 W cm^-2^ for 10 min. Tumor volume and mouse weight were measured every two days until the tumor volume reached 1500 mm³. The tumor volume was calculated using the following formula: Tumor Volume = Tumor Length × Tumor Width²/2.

Mice were euthanized 10 days post-treatment, then tumor and lymph node tissues were collected. These tissues were rinsed three times with PBS, and dissected into small fragments (1 mm³). These fragments were digested in a buffer containing 2 mg/mL collagenase IV at 37°C for 2 hours. The resulting cell suspension was filtered through a 70 μm cell strainer and incubated with red blood cell lysis buffer for 2 min. After centrifugation, the remaining cells were washed three times with staining buffer and labeled with the following fluorophore-conjugated antibodies according to the manufacturer’s instructions: CD3-FITC, CD8-PerCP, GZMB-PE, TOX-PE, IFNγ-APC, CD11c-APC, CD80-FITC, and CD86-PE. Immune cell activation in tumors was assessed via flow cytometry.

1. To investigate the *in vivo* therapeutic effects of IR780@TVA LNPs combined with anti-PD-L1 antibodies, a melanoma model was established using a consistent methodology. Once the tumor volume reached approximately 60 mm³, the mice were randomly divided into five groups. The mice were treated with PBS (G1), anti-PD-L1 (G2, Anti-PDL1: 5 mg/kg×3 times), IR780@TVA LNPs + Laser (G3, IR780: 4 mg/kg, TVA: 12 mg/kg), IR780@LA LNPs + Laser + anti-PD-L1 (G4, IR780: 4 mg/kg, Anti-PDL1: 5 mg/kg×3 times), and IR780@TVA LNPs + Laser + anti-PD-L1 (G5, IR780: 4 mg/kg, TVA: 12 mg/kg, Anti-PDL1: 5 mg/kg×3 times). Laser irradiation was conducted 12 hours post-treatment, and irradiation was performed at 808 nm with a power density of 1.0 W cm^-2^ for 10 min. Anti-PD-L1 was administered via intraperitoneal injection 24 hours after the laser irradiation. Subsequently, tumor volume, mouse weight, and survival rates were monitored. Tumor tissues and tumor-draining lymph nodes (TDLNs) were collected and analyzed by flow cytometry. Cells were stained with the following antibodies according to the manufacturer’s instructions: CD3-FITC, CD8-PerCP, GZMB-PE, TOX-PE, IFNγ-APC, CD11c-APC, CD80-FITC, and CD86-PE. The blood samples were harvested to measure serum levels of alanine aminotransferase (ALT), aspartate aminotransferase (AST), alkaline phosphatase (ALP), creatinine (CREA), and urea (UREA). Major organs (heart, liver, spleen, lungs, and kidneys) and tumor tissues were excised, fixed, paraffin-embedded, sectioned, and stained with hematoxylin and eosin (H&E) for histopathological evaluation. For the mice that were cured, a rechallenge assay was performed 50 days later on the opposite side of the back (healthy mice were used as control). The spleens were then collected to analyze the proportion of effector memory T cells (T_em_, CD3^+^/CD8^+^/CD44^+^/CD62^-^) and central memory T cells (T_cm_, CD3^+^/CD8^+^/CD44^+^/CD62^+^) in the mice.

3) The RM-1 prostate cancer model was established for evaluating the therapeutic efficacy of IR780@TVA LNPs on “cold” tumors. 5 × 10^5^ of RM-1 cells were injected into the right flank of male C57BL/6 mice. Once the tumor volume reached approximately 100 mm³, the mice were randomly divided into three groups and treated with PBS (G1), IR780@LA LNPs + Laser (G2), and IR780@TVA LNPs + Laser (G3). Laser irradiation was conducted 12 hours post-treatment, then tumor tissues and lymph nodes were collected for flow cytometry analysis. In addition, the collected tumor tissues were further used for transcriptome sequencing analysis. Briefly, tumor tissues harvested 24 hours post-laser irradiation, tumor tissues were collected and the total RNA was extracted using Trizol (Invitrogen, Carlsbad, CA, USA) according to the manufacturer's instructions. Subsequently, tumor RNA was analyzed by Sangon Biotech (Shanghai, China).

4) *In vivo* therapeutic efficacy evaluation of IR780@TVA LNPs combined with PD-L1 in the prostate cancer model. The prostate cancer model was established using the above-mentioned method. When the tumor volume reached approximately 100 mm³, the mice were randomly divided into four groups. The mice were treated with PBS (G1), anti-PD-L1 (G2), IR780@LA LNPs + Laser + anti-PD-L1 (G3), and IR780@TVA LNPs + Laser + anti-PD-L1 (G4). Laser irradiation was performed 12 hours post-treatment, followed by an intraperitoneal injection of anti-PD-L1 24 hours after the laser irradiation. Tumor volume and mouse weight were measured every two days. And tumor tissues were collected for immunofluorescence staining analysis.

5) To evaluate the therapeutic efficacy of IR780@TVA LNPs in a challenging, clinically relevant setting, a large-volume tumor model was established. Male C57BL/6 mice were subcutaneously inoculated with 5 × 10⁵ RM-1 cells on the right flank. Tumor growth was monitored until the volume reached approximately 300 mm³. At this stage, mice were randomly allocated into five treatment groups: PBS (G1), radiotherapy alone (G2), IR780@TVA LNPs + Laser (G3), radiotherapy + anti-PD-L1 (G4), and IR780@TVA LNPs + Laser + anti-PD-L1 (G5). Treatments were administered as per the respective protocols. Specifically, laser irradiation (for G3 and G5) or a single dose of 10 Gy radiotherapy (for G2 and G4) was delivered 12 hours after the corresponding systemic injections. Following treatment, tumor tissues and draining lymph nodes were harvested for subsequent immune profiling by flow cytometry.

6) To further assess the potential of our strategy in controlling systemic metastasis in an advanced setting, a large‑volume tumor model was employed. Male C57BL/6 mice were inoculated subcutaneously on the right flank with 5 × 10⁵ RM‑1 cells. Once the tumor volume exceeded approximately 300 mm³, the tumor‑bearing mice were randomly assigned to five treatment groups: (G1) PBS, (G2) radiotherapy alone, (G3) IR780@TVA LNPs + Laser, (G4) radiotherapy + anti‑PD‑L1, and (G5) IR780@TVA LNPs + Laser + anti‑PD‑L1. One day after the respective local treatments, mice received an intravenous injection of 5 × 10⁵ luciferase‑expressing RM‑1 (Luc‑RM1) cells to simulate hematogenous dissemination. Metastatic progression was monitored longitudinally by bioluminescent imaging. On day 15, mice were euthanized and bilateral lung tissues were collected. The lungs were fixed in 4% paraformaldehyde, embedded in paraffin, and sectioned for hematoxylin and eosin (H&E) staining. Microscopic examination was performed to evaluate the number, size, and tissue‑invasive pattern of metastatic foci. Imaging data and histopathological findings were integrated to quantitatively assess the anti‑metastatic efficacy of each treatment regimen.

**Statistical analysis**

GraphPad Prism software was used for statistical analysis. All data was presented as mean ± SD. Statistical analysis was conducted using a two-tailed Student’s t-test to compare two groups, and one-way analysis of variance (ANOVA) with Tukey’s post hoc test for comparisons among multiple groups, and two‑way ANOVA followed by simple effects analysis with Bonferroni correction for multiple comparisons. For all figures, ns: no significant difference, *p < 0.05, **p < 0.01, ***p < 0.001, ****p < 0.0001.


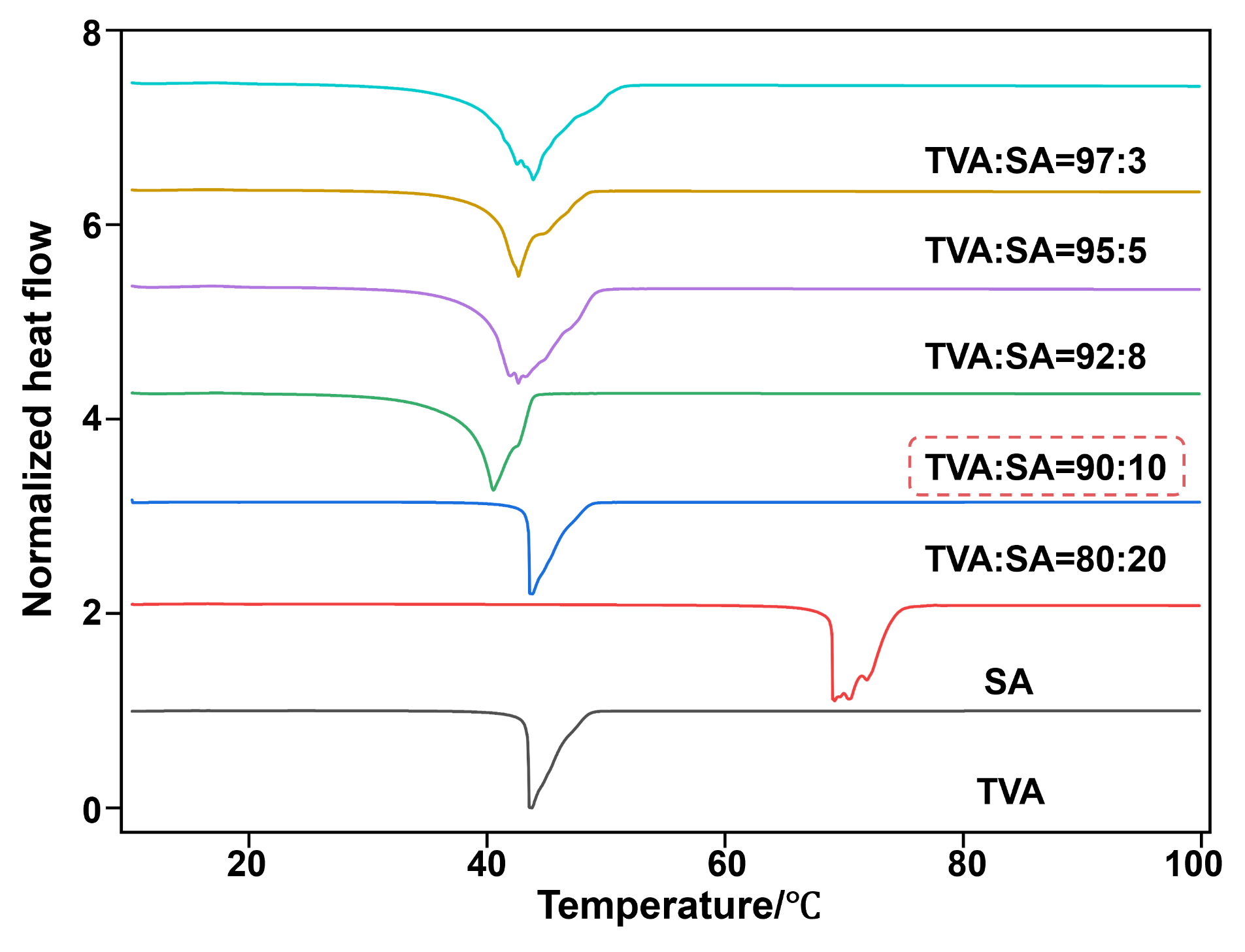


**Figure S1.** Melting Points of *trans*-vaccenic acid (TVA)-stearic acid (SA) eutectic mixture at different weight ratios.

**
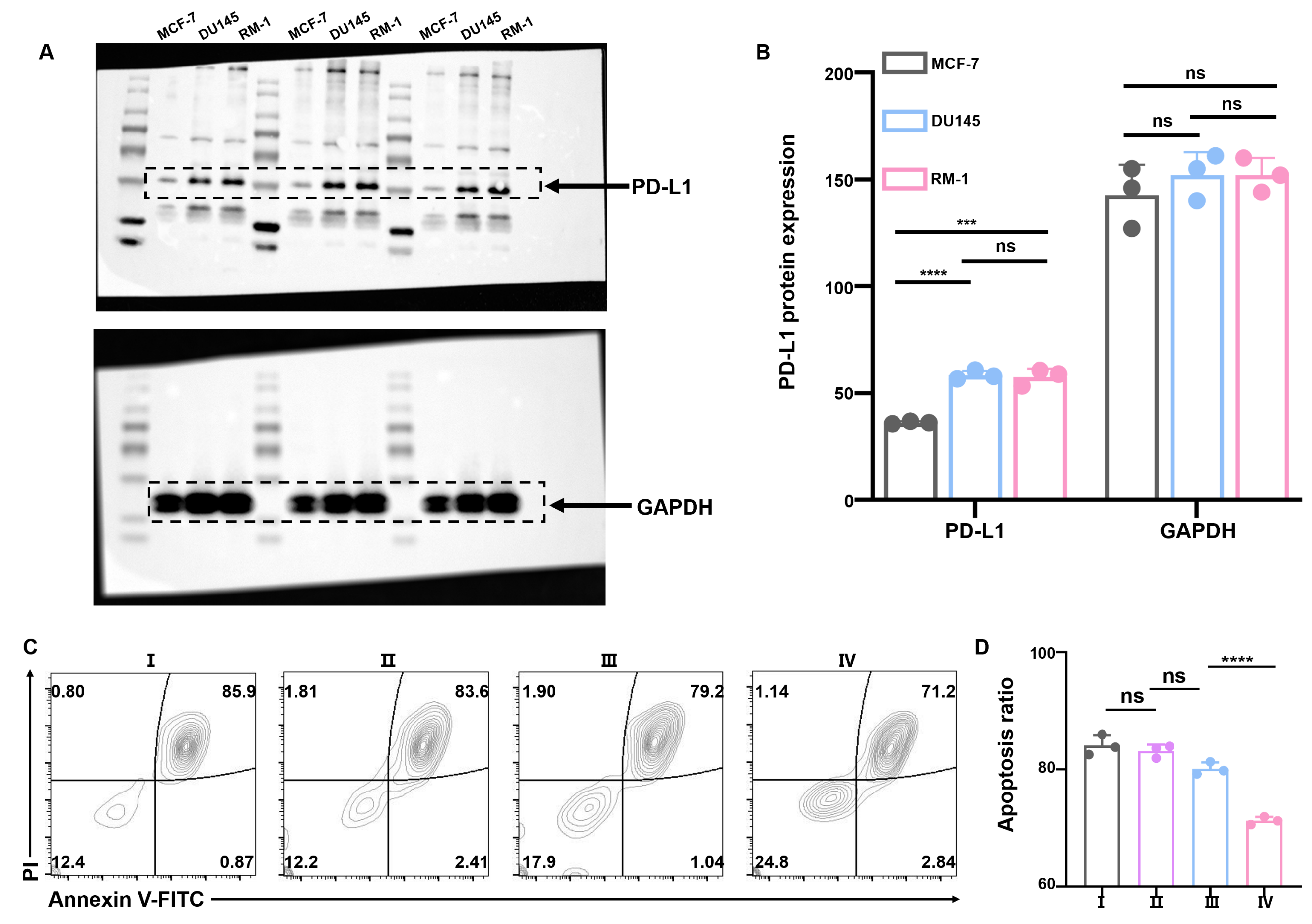
**

**Figure S2. A)** Western blotting results of PD-L1 and GAPDH expression in MCF-7, DU145 and RM-1 cells. **B)** Quantification of endogenous PD-L1 protein expression in MCF-7, DU145, and RM-1 tumor cell lines (Data represent: mean ± SD, n=3). **C, D)** Flow cytometry results of CD8^+^ T cell apoptosis in the co-culture model of CD8^+^ T/RM-1 cells and the corresponding quantitative results [PBS (I), IR780@LA LNPs without laser pretreatment (II), IR780@TVA LNPs without laser pretreatment (III), IR780@TVA LNPs pretreated with laser (IV).] (Data represent: mean ± SD, n=3) ns: no significant difference, *p < 0.05, **p < 0.01, ***p < 0.001, ****p < 0.0001 as determined by one-way ANOVA followed by Tukey's multiple comparison (B, D).

**
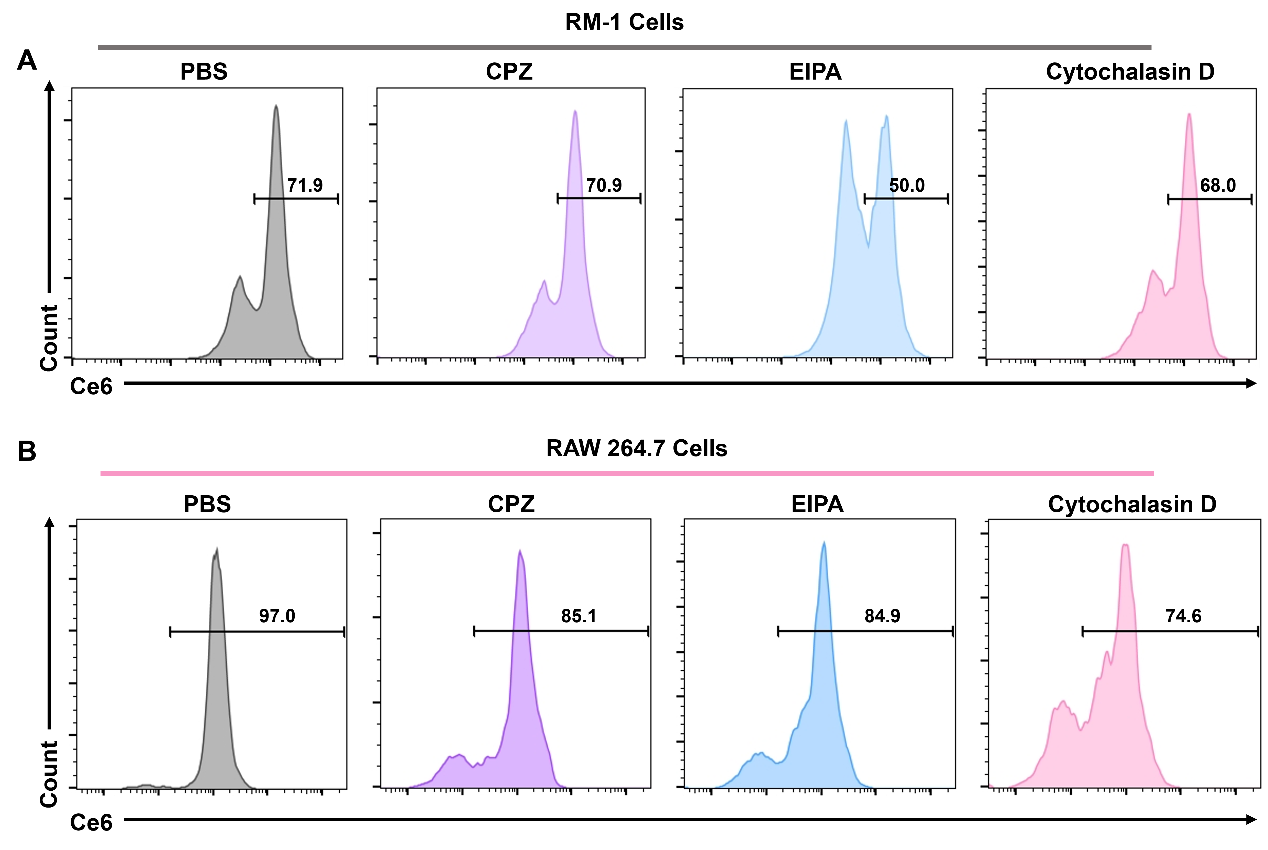
**

**
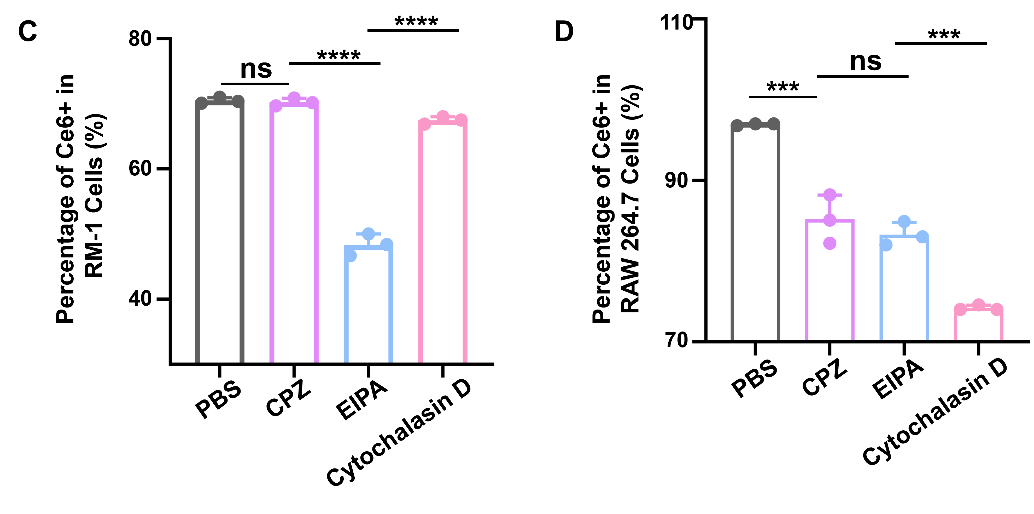
**

**Figure S3. A, B)** Flow cytometric analysis of the uptake of Ce6-labeled IR780@TVA LNPs by tumor cell and macrophages following 24 h treatment with specific pharmacological inhibitors targeting distinct endocytic pathways. **C, D)** Quantitative data of **Figure S3A, B** (Data represent: mean ± SD, n=3) ns: no significant difference, *p < 0.05, **p < 0.01, ***p < 0.001, ****p < 0.0001 as determined by one-way ANOVA followed by Tukey's multiple comparison (C, D).


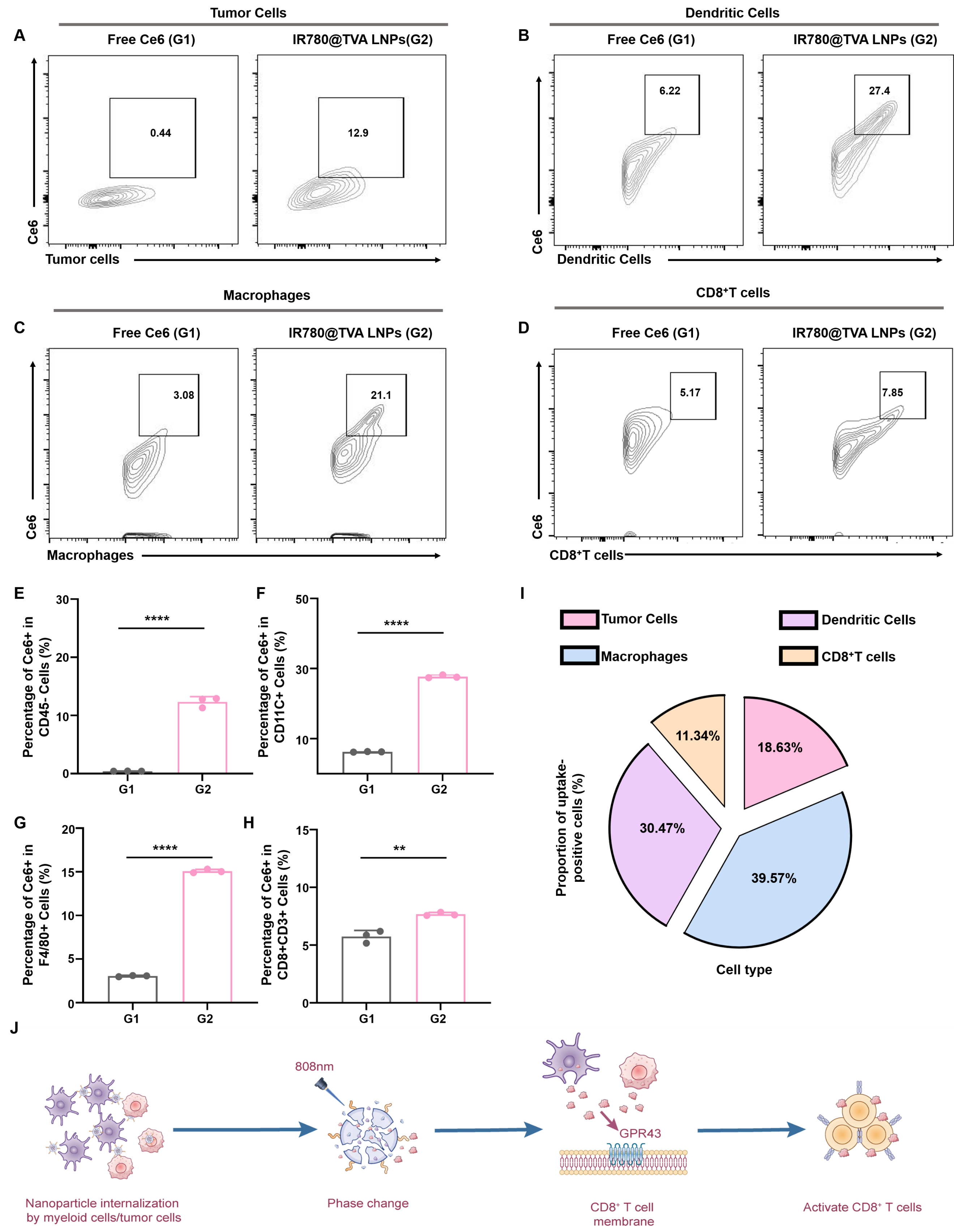


**Figure S4. Cellular Uptake of Nanoparticles in Tumor Microenvironment. A-D)** Representative flow cytometry plots illustrating the uptake of Ce6-IR780@TVA LNPs by tumor cells, dendritic cells (DCs), macrophages and T cells at 6 h post-injection. **(E-H)** Quantitative analysis showing the percentage of Ce6-positive cells for each corresponding cell type at 6 h post-injection (Data represent: mean ± SD, n=3) ns: no significant difference, *p < 0.05, **p < 0.01, ***p < 0.001, ****p < 0.0001 as determined by one-way ANOVA followed by Tukey's multiple comparison (E, F, G, H). **I)** Uptake distribution among different cell types. Pink indicates tumor cells, blue indicates dendritic cells (DCs), purple indicates macrophages, and yellow indicates CD8^+^T cells. **J)** Schematic representation of the “bystander effect” cascade mediated by IR780@TVA LNPs.


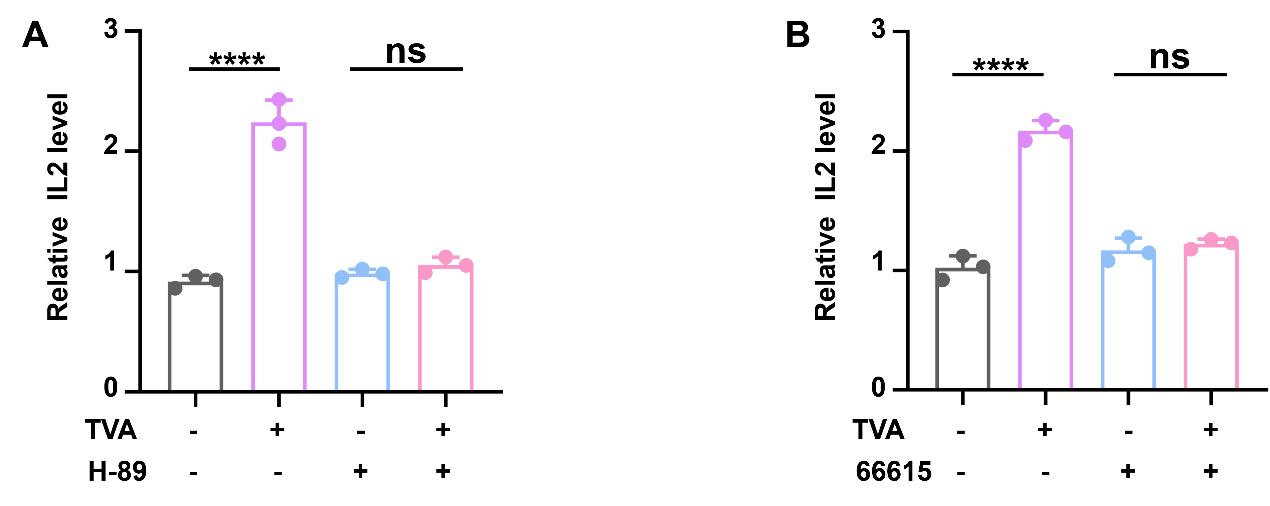


**Figure S5. A)** Effect of treatment with PKA inhibitor H89 on TVA-dependent CD8+ T cell activation assessed by IL-2. **B)** Effects of treatment with CREB inhibitor 666-15 on TVA-dependent CD8+ T cell activation assessed by IL-2. (Data represent: mean ± SD, n=3) ns: no significant difference, *p < 0.05, **p < 0.01, ***p < 0.001, ****p < 0.0001 as determined by one-way ANOVA followed by Tukey's multiple comparison (A, B).

**
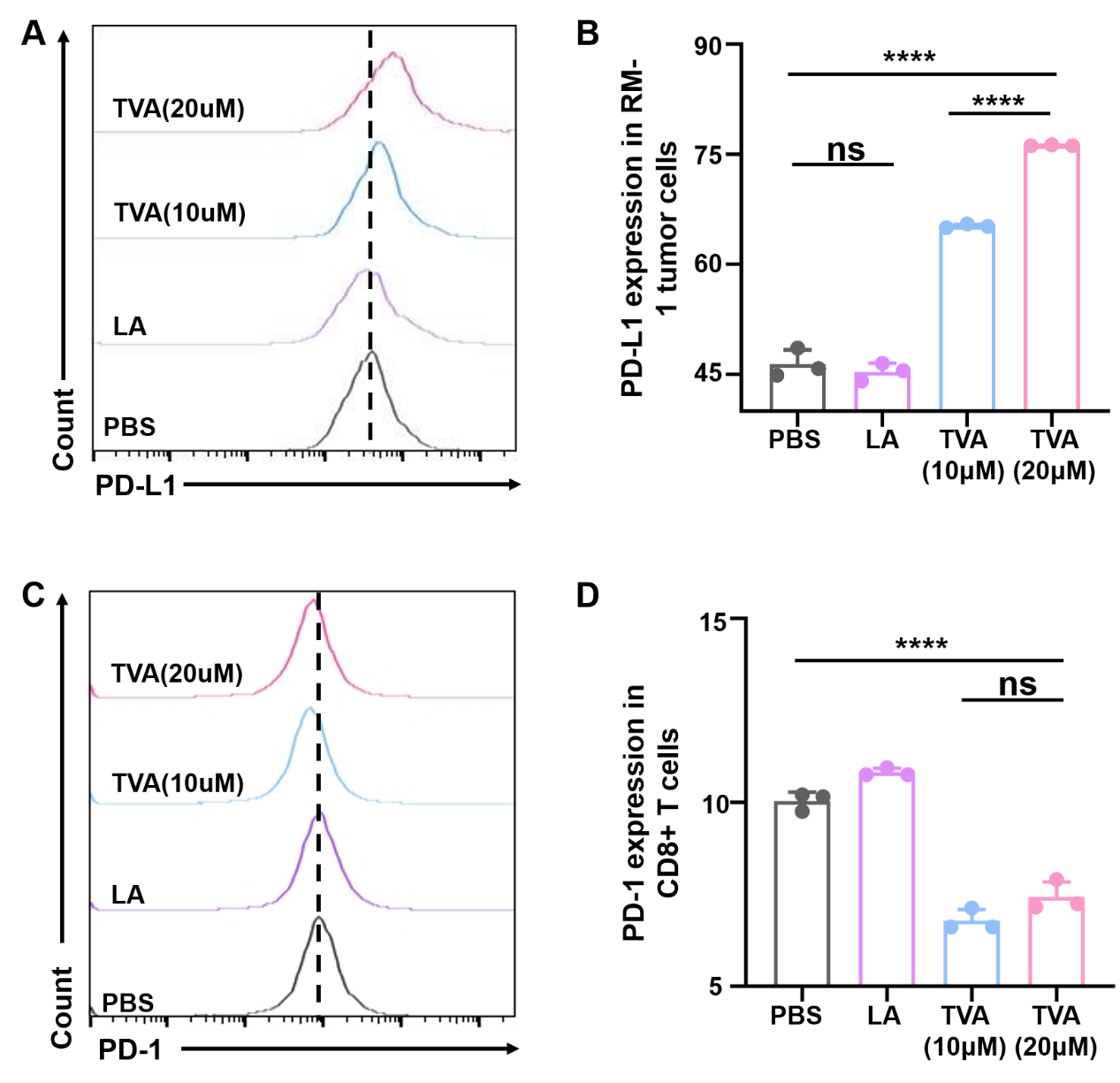
 Figure S6. A)** Flow cytometric analysis of PD-L1 expression on RM-1 tumor cells after treatment with PBS, LA (20 μM), or TVA (10 μM and 20 μM). **B)** Quantification of PD-L1⁺ tumor cells under different treatment conditions. **C)** Flow cytometric analysis of PD-1 expression on CD8^+^ T cells after treatment with PBS, LA (20 μM), or TVA (10 μM and 20 μM). **D)** Quantification of PD-1⁺ CD8^+^ T cells under different treatment conditions. (Data represent: mean ± SD, n=3) ns: no significant difference, *p < 0.05, **p < 0.01, ***p < 0.001, ****p < 0.0001 as determined by one-way ANOVA followed by Tukey's multiple comparison (B, D).

**
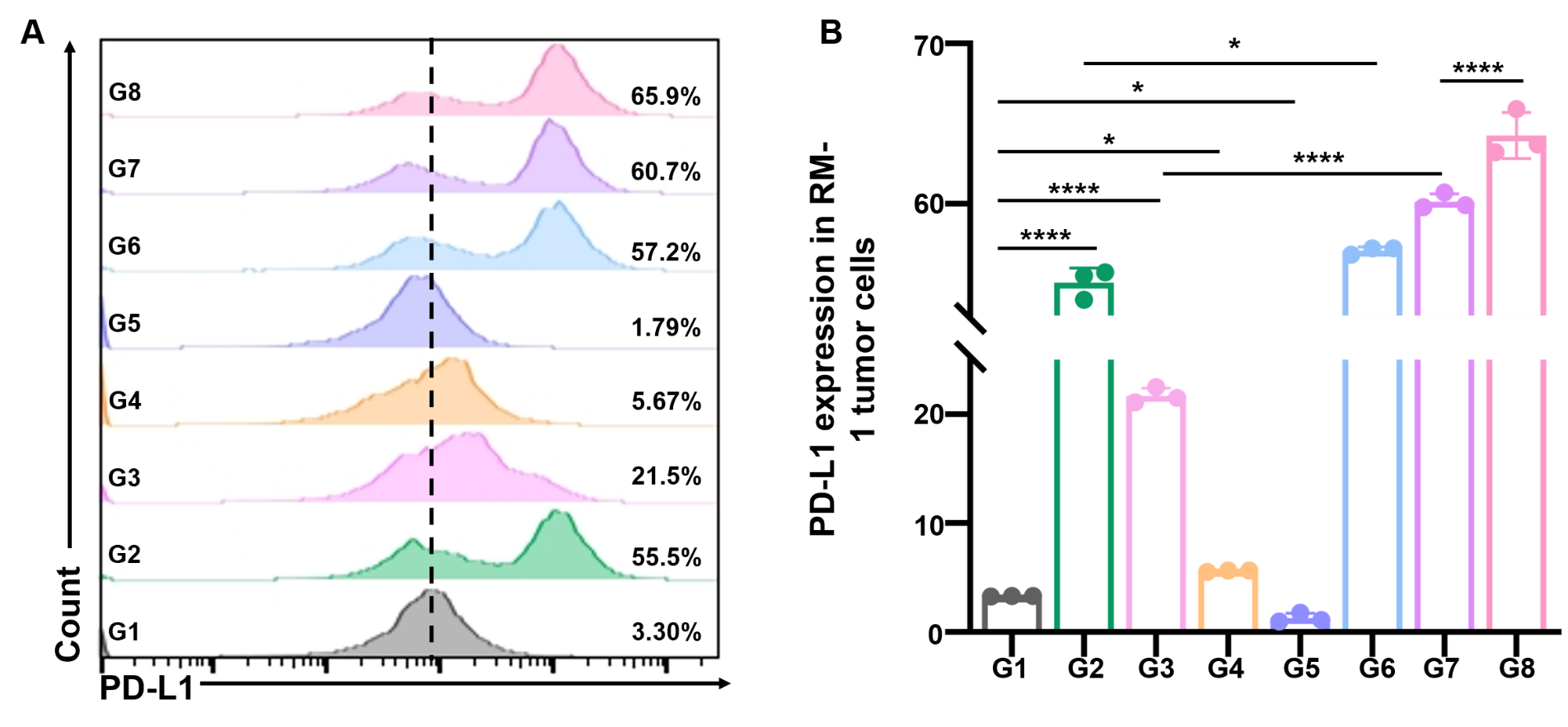
Figure S7. A)** Percentage of PD-L1-positive cells in RM-1 cells. **B)** Relative quantification of flow cytometry analysis for the PD-L1^+^ cells in RM-1 cells (Data represent: mean ± SD, n=3) ns: no significant difference, *p < 0.05, **p < 0.01, ***p < 0.001, ****p < 0.0001 as determined by one-way ANOVA followed by Tukey's multiple comparison (B). PBS (G1), IR780@LA LNPs + Laser (G2, PTT stress only), recombinant IFN‑γ (20 ng/mL, G3), free TVA (G4), free TVA + PKA inhibitor H‑89 (G5), IR780@TVA LNPs + Laser (G6), IFN‑γ + free TVA (G7), and IR780@TVA LNPs + Laser + IFN‑γ (G8).


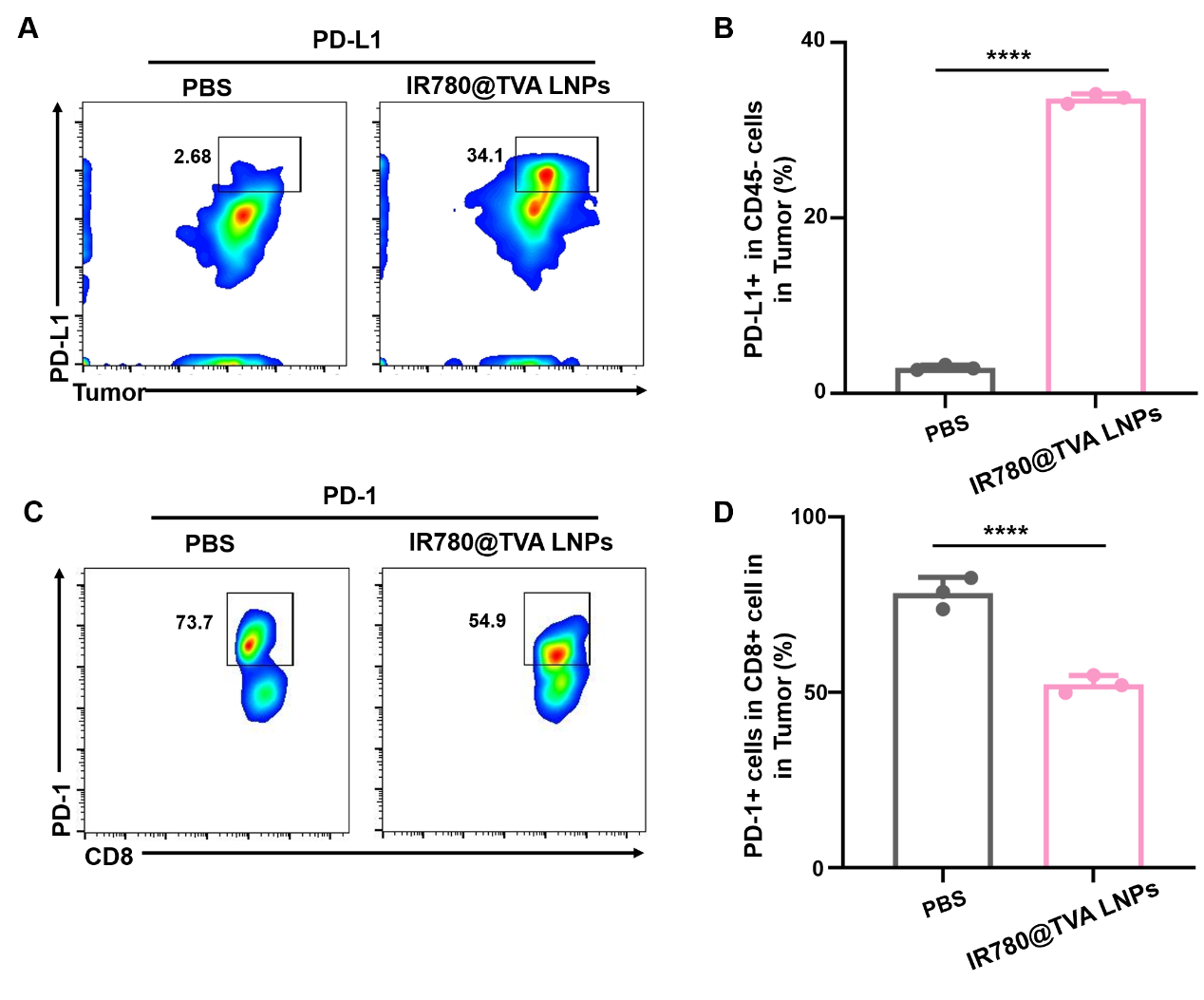
**Figure S8.** **A)** Percentage of PD-L1-positive CD45^-^ (PD1^+^/CD8^-^) T cells in tumors of mice on day 10. **B)** Relative quantification of flow cytometry analysis for the PD-L1^+^ cells in CD45^-^ cells in tumors of mice on day 10 (Data represent: mean ± SD, n=3). **C)** Percentage of PD-1-positive CD8^+^ (PD1^+^/CD8^+^) T cells in tumors of mice on day 10. **D)** Relative quantification of flow cytometry analysis for the PD-1^+^ cells in CD8^+^ cells in tumors of mice on day 10 (Data represent: mean ± SD, n=3) ns: no significant difference, *p < 0.05, **p < 0.01, ***p < 0.001, ****p < 0.0001 as determined by one-way ANOVA followed by Tukey's multiple comparison (B, D).


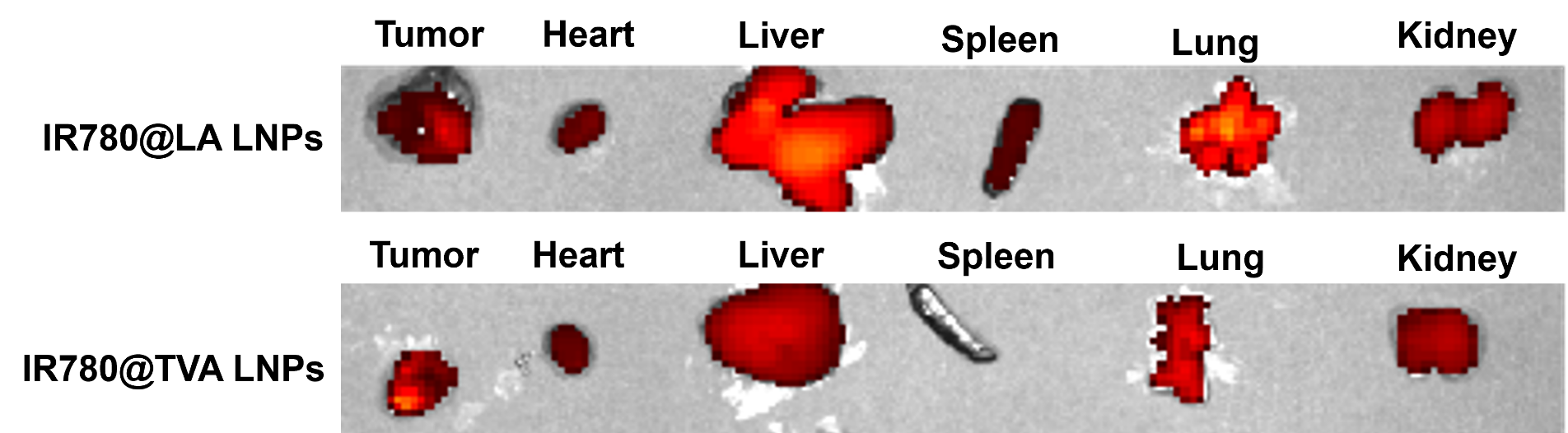


**Figure S9.** Representative *ex vivo* imaging demonstrates tumor-targeting ability of IR780@TVA LNPs and IR780@LA LNPs at 24 h post-treatments.


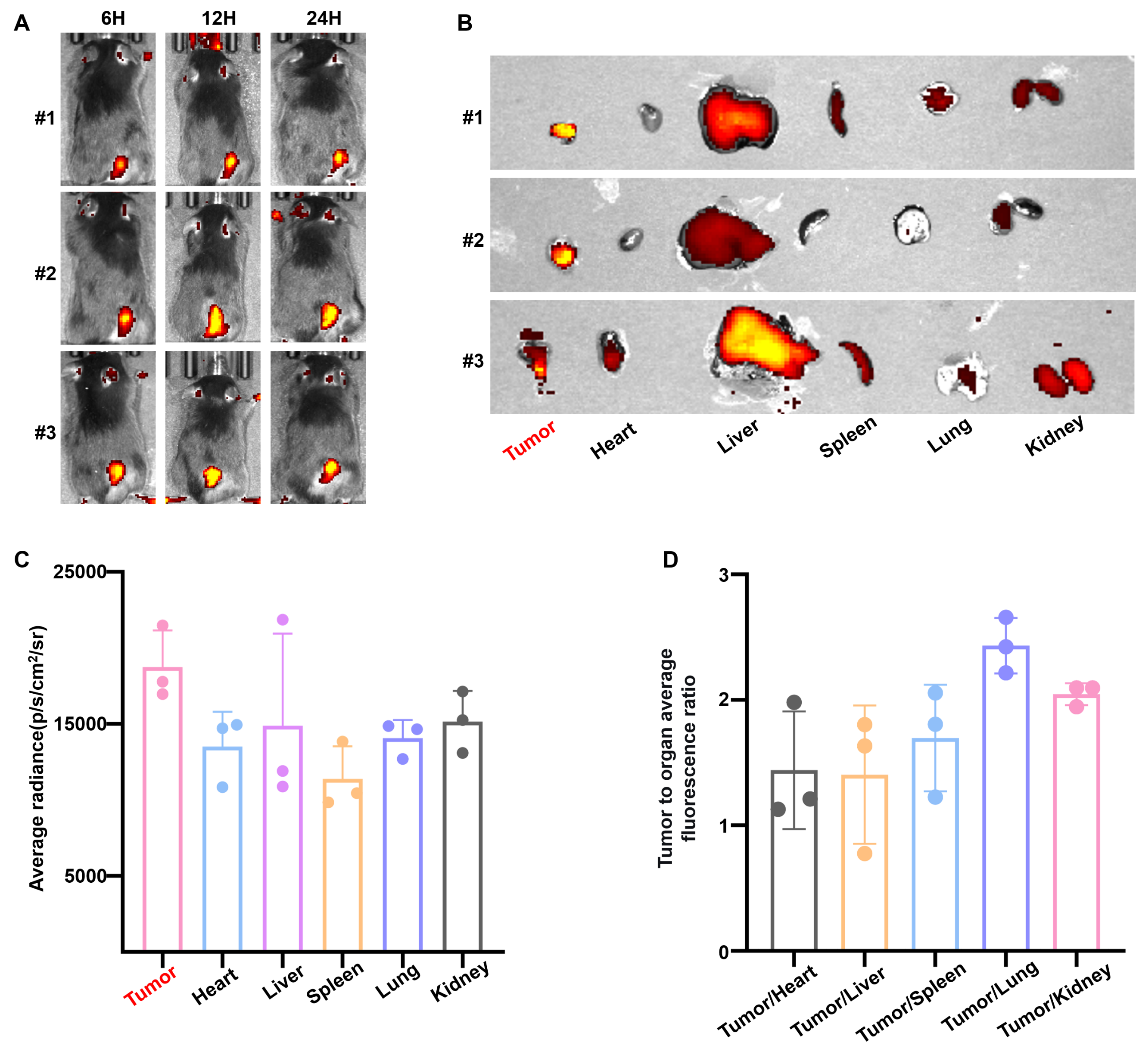


**Figure S10. A)** *In vivo* biodistribution analyzed by fluorescence imaging of tumor-bearing mice at 6, 12, and 24 hours after intravenous injection of IR780@TVA LNPs. **B)** Ex vivo imaging demonstrates tumor-targeting ability of IR780@TVA LNPs at 24 h post-treatments. **C)** Average radiant efficiency diagram of fluorescence in tumor and various tissues (heart, liver, spleen, lung, kidney) (n = 3). **D)** Average fluorescence signal ratios of tumor over normal organs (n=3).
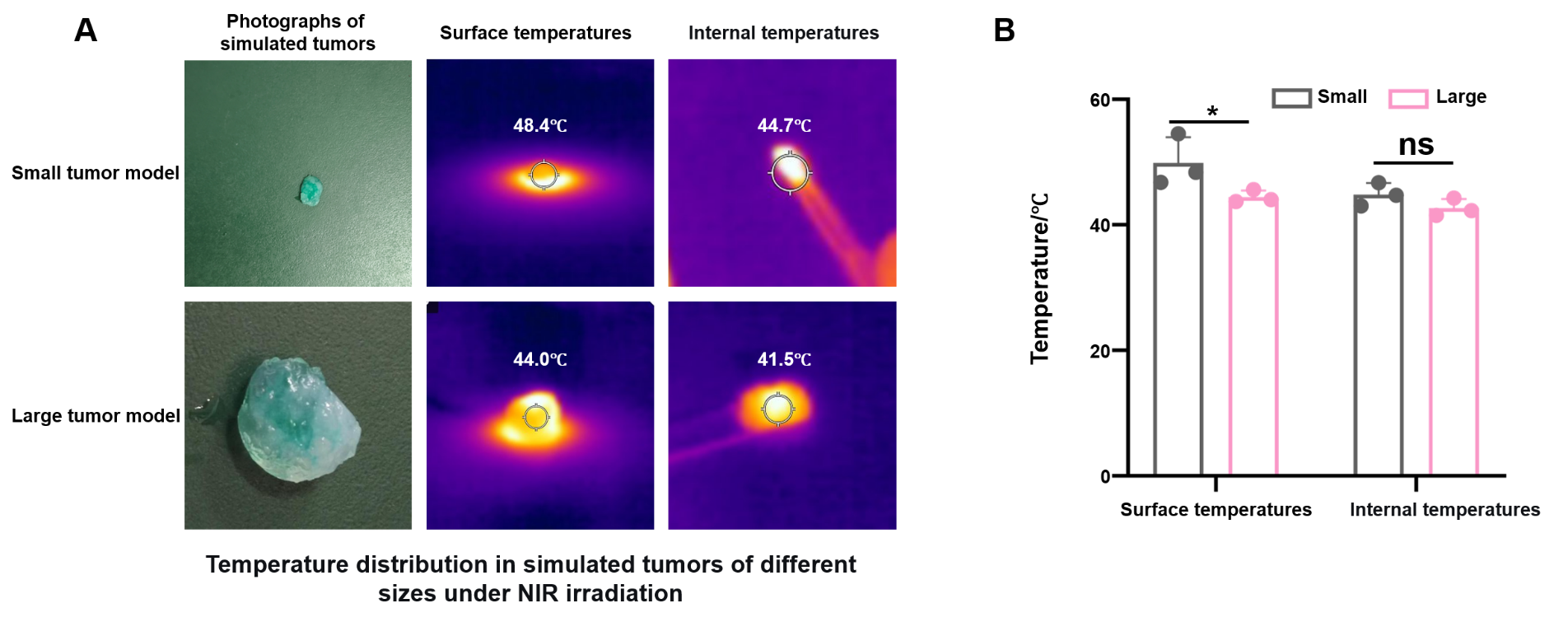


**Figure S11. A)** Temperature distribution in calcium alginate hydrogel-simulated tumors of different sizes under NIR irradiation. **B)** Quantification of surface and internal (the cross section) temperatures (Data represent: mean ± SD, n=3) ns: no significant difference, *p < 0.05, **p < 0.01, ***p < 0.001, ****p < 0.0001 as determined by two‑way ANOVA followed by simple effects analysis with Bonferroni correction for multiple comparison (B).


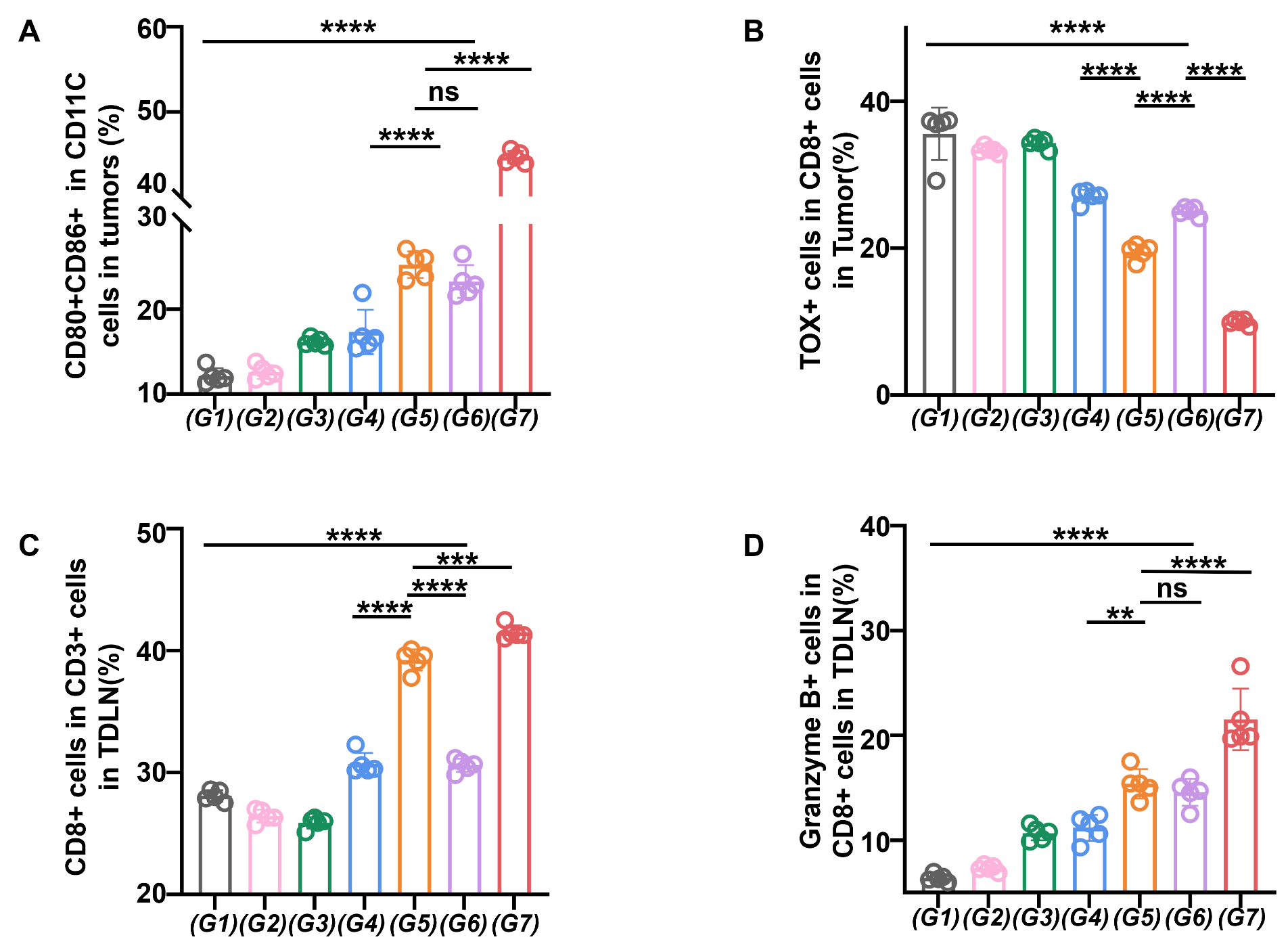


**Figure S12. Flow cytometry results of immune cells in tumors in the B16F10 (B16F10-LUC) tumor-bearing mice model.** **A)** Relative quantification of flow cytometry analysis for the CD80^+^CD86^+^ cells in CD11C^+^ cells in tumors of mice on day 10 (Data represent: mean ± SD, n=5). **B)** Relative quantification of flow cytometry analysis for the TOX^+^ cells in CD8^+^ T cells in tumors of mice on day10 (Data represent: mean ± SD, n=5). **C)** Relative quantification of flow cytometry analysis for the CD8^+^ cells in CD3^+^ T cells in TDLNs of mice on day 10 (Data represent: mean ± SD, n=5). **D)** Relative quantification of flow cytometry analysis for the GZMB^+^ in CD8^+^ T cells in TDLNs of mice on day 10 (Data represent: mean ± SD, n=5) ns: no significant difference, *p < 0.05, **p < 0.01, ***p < 0.001, ****p < 0.0001 as determined by one-way ANOVA followed by Tukey's multiple comparison (A, B, C, D).

**
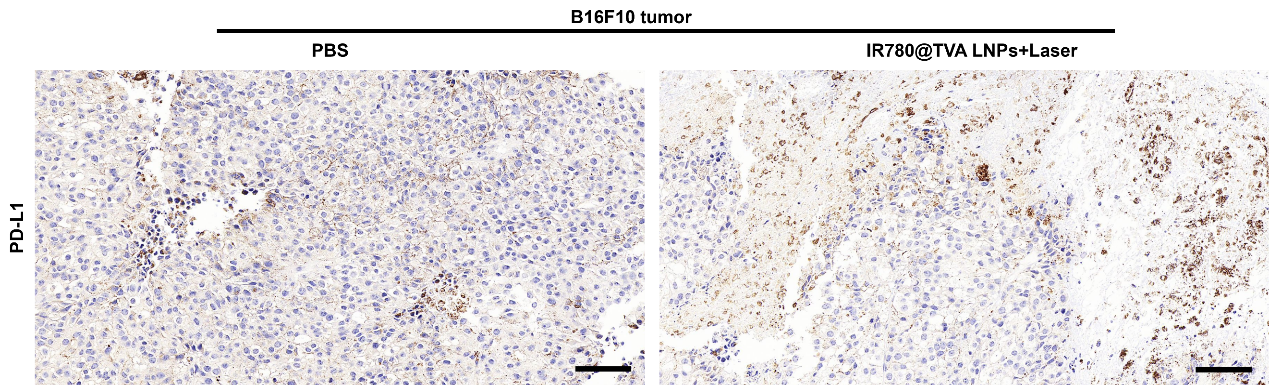
**

**Figure S13.** **IHC staining of PD-L1 expression in tumours of PBS or IR780@TVA LNPs+Laser treated mice (Scale bars, 100 μm).**

**
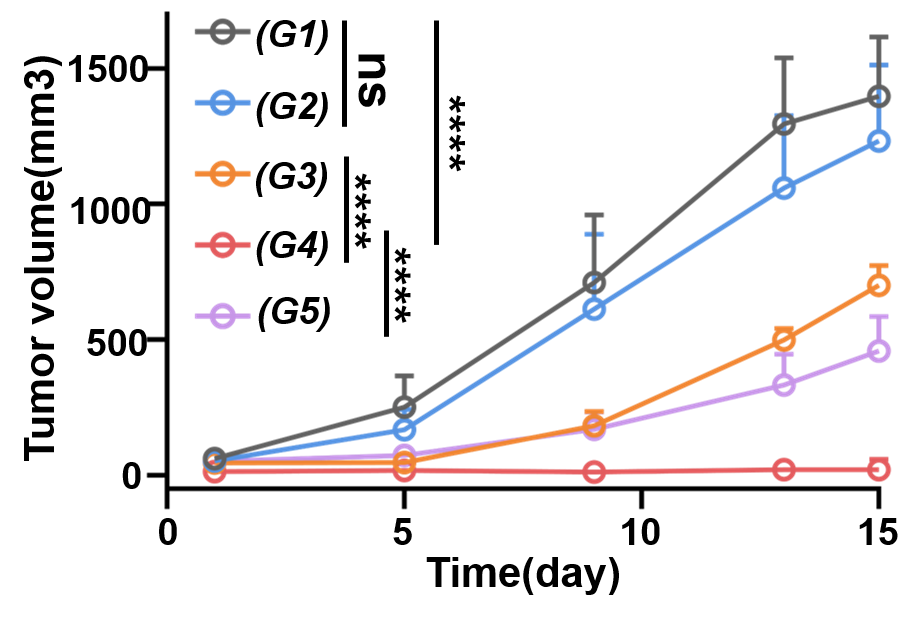
**

**Figure S14.** Average tumor growth curves in B16F10 melanoma model after different treatments (Data represent: mean ± SD, n=5). ns: no significant difference, *p < 0.05, **p < 0.01, ***p < 0.001, ****p < 0.0001 as determined by one‑way ANOVA followed by Tukey's multiple comparison

**
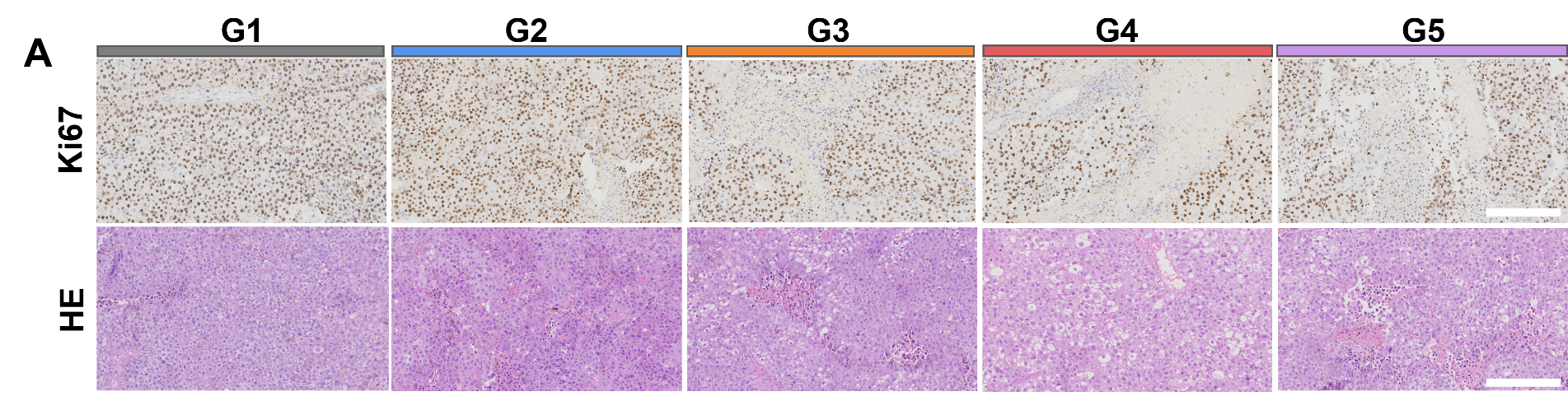


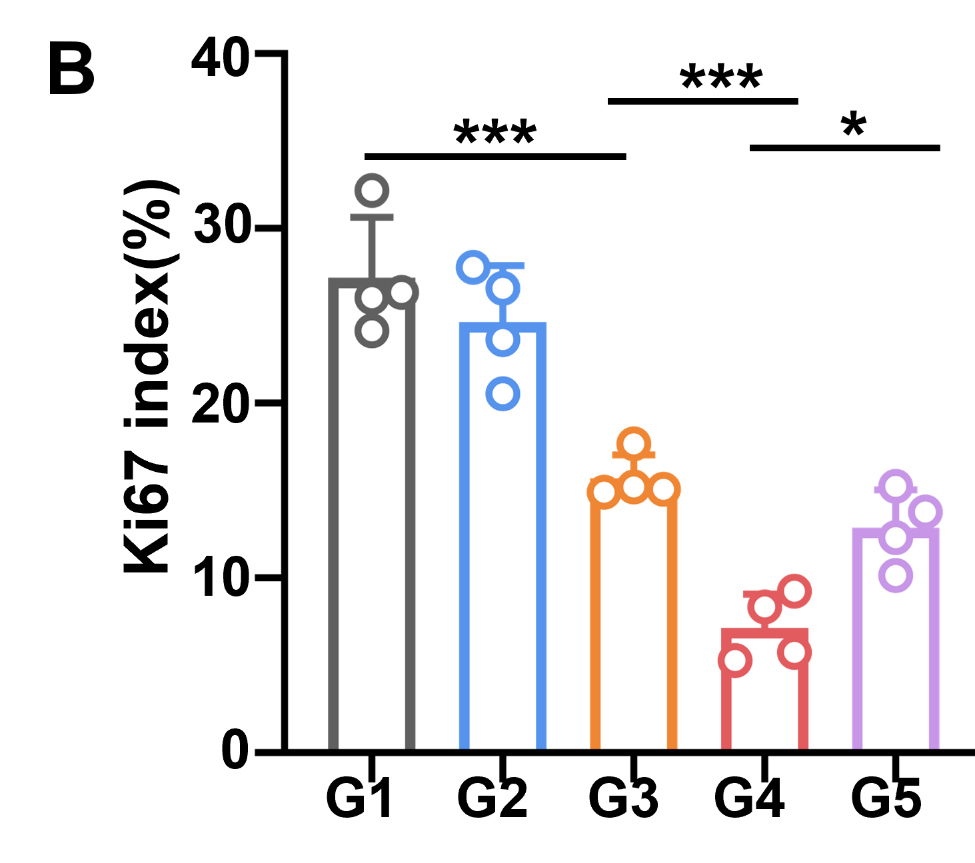
**

**F****igure S15.** **A)** Ki67 staining and H&E staining of B16F10 tumor tissues from the mice receiving different treatments (scale bar: 200 μm). **B)** Semi-quantitative analysis of Ki67 expression in tumor tissue (Data represent: mean ± SD, n=4) ns: no significant difference, *p < 0.05, **p < 0.01, ***p < 0.001, ****p < 0.0001 as determined by one-way ANOVA followed by Tukey's multiple comparison (B)


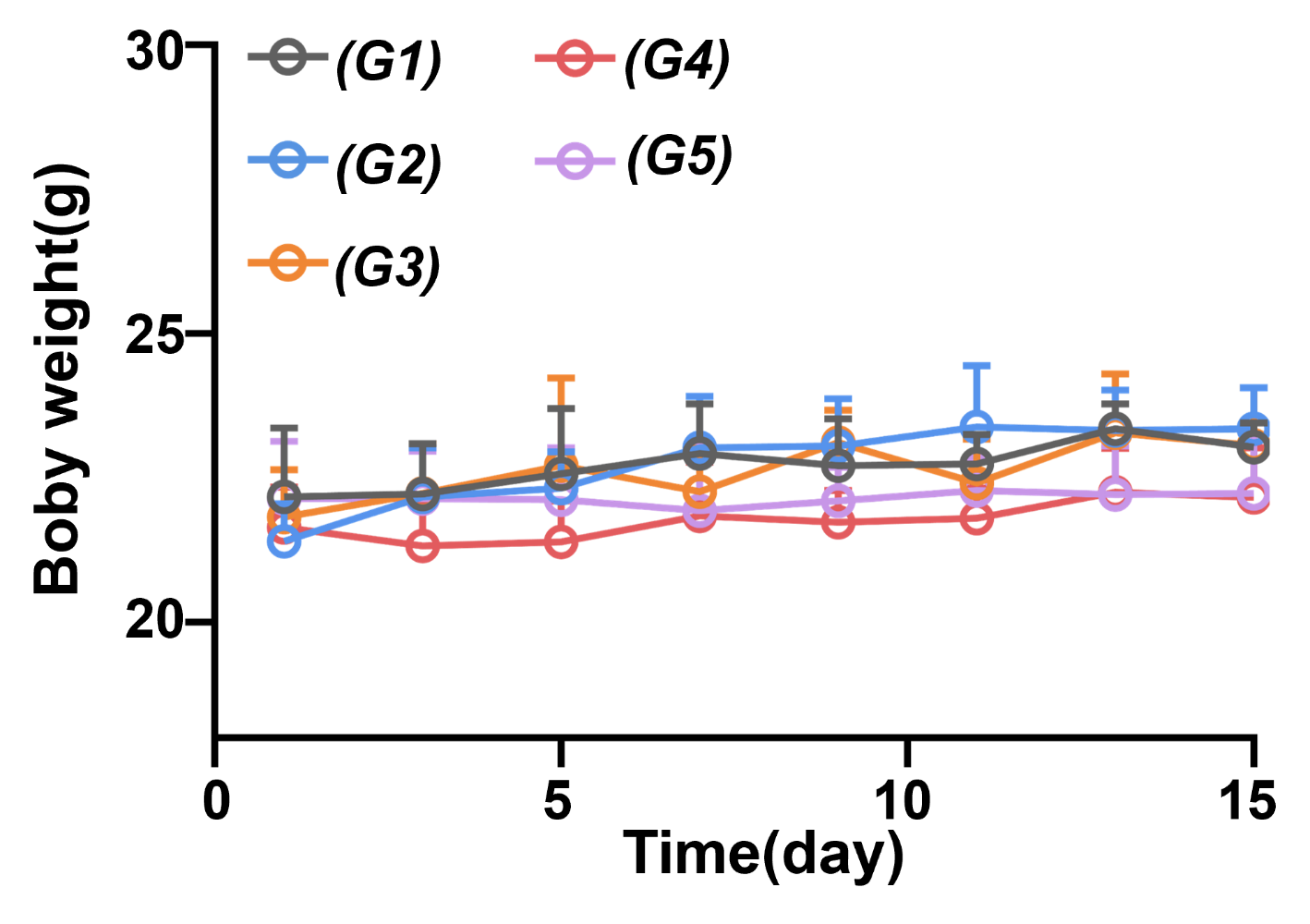


**Figure S16.** Changes in body weight of B16F10-LUC tumor-bearing mice following treatment with different groups (Data represent: mean ± SD, n=5).

**
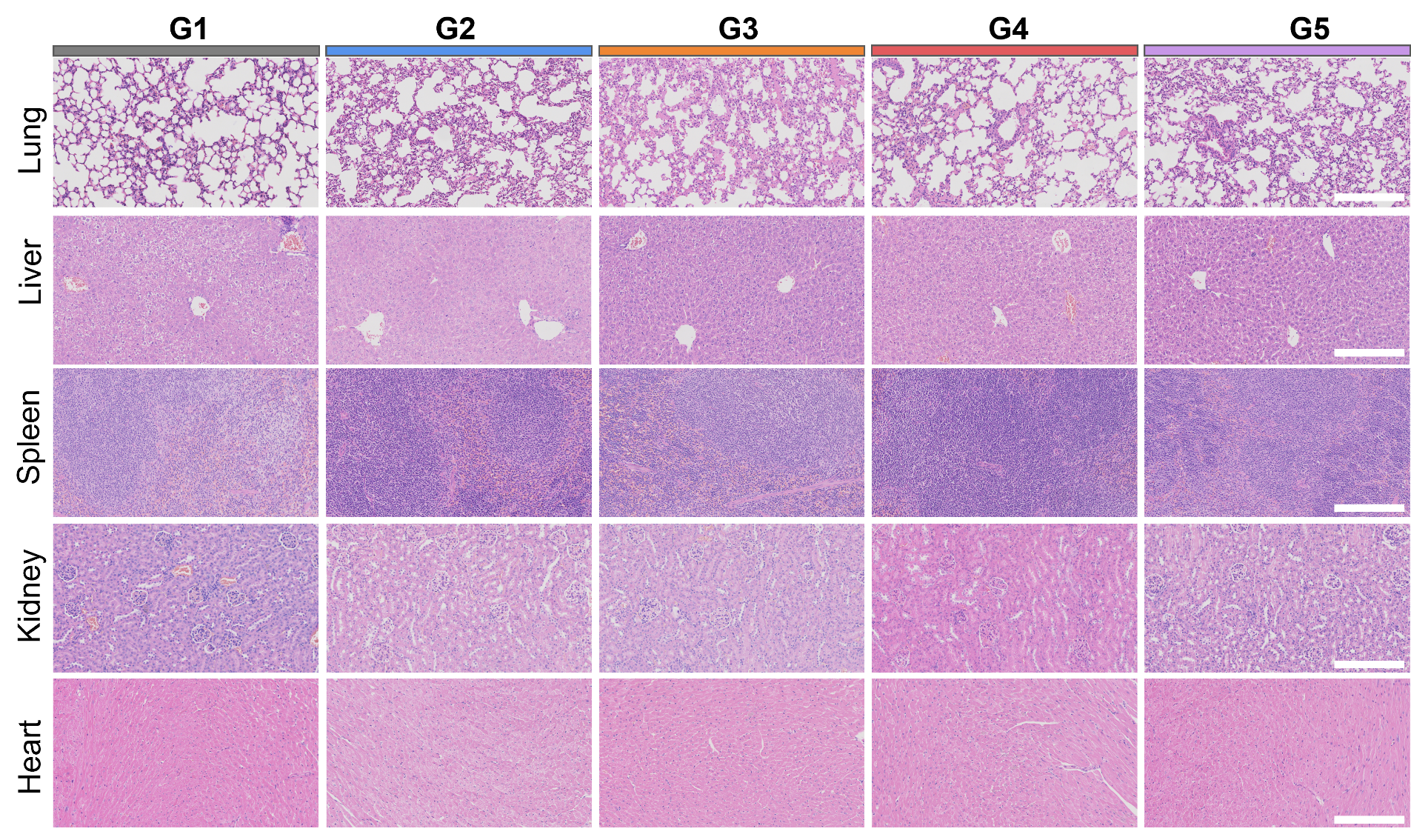
**

**Figure S17.** Histopathological images of the main organs, containing lung, liver, spleen, kidney and heart obtained from the B16F10-LUC tumor-bearing mice. Scale bars: 200 μm.

**
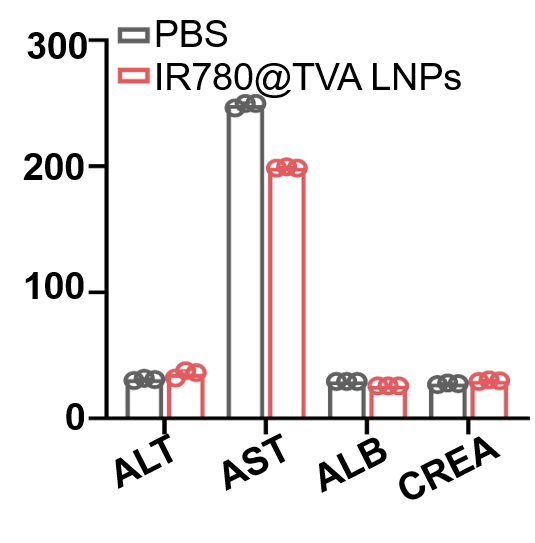
**

**Figure S18.** Blood routine test of mice in PBS group and IR780@TVA LNPs group after 10 days of treatment. Unit of measurement: ALT (U/L), AST (U/L), ALB (g/L), CREA (μM). Data are shown as mean ± SD (n = 3).

**
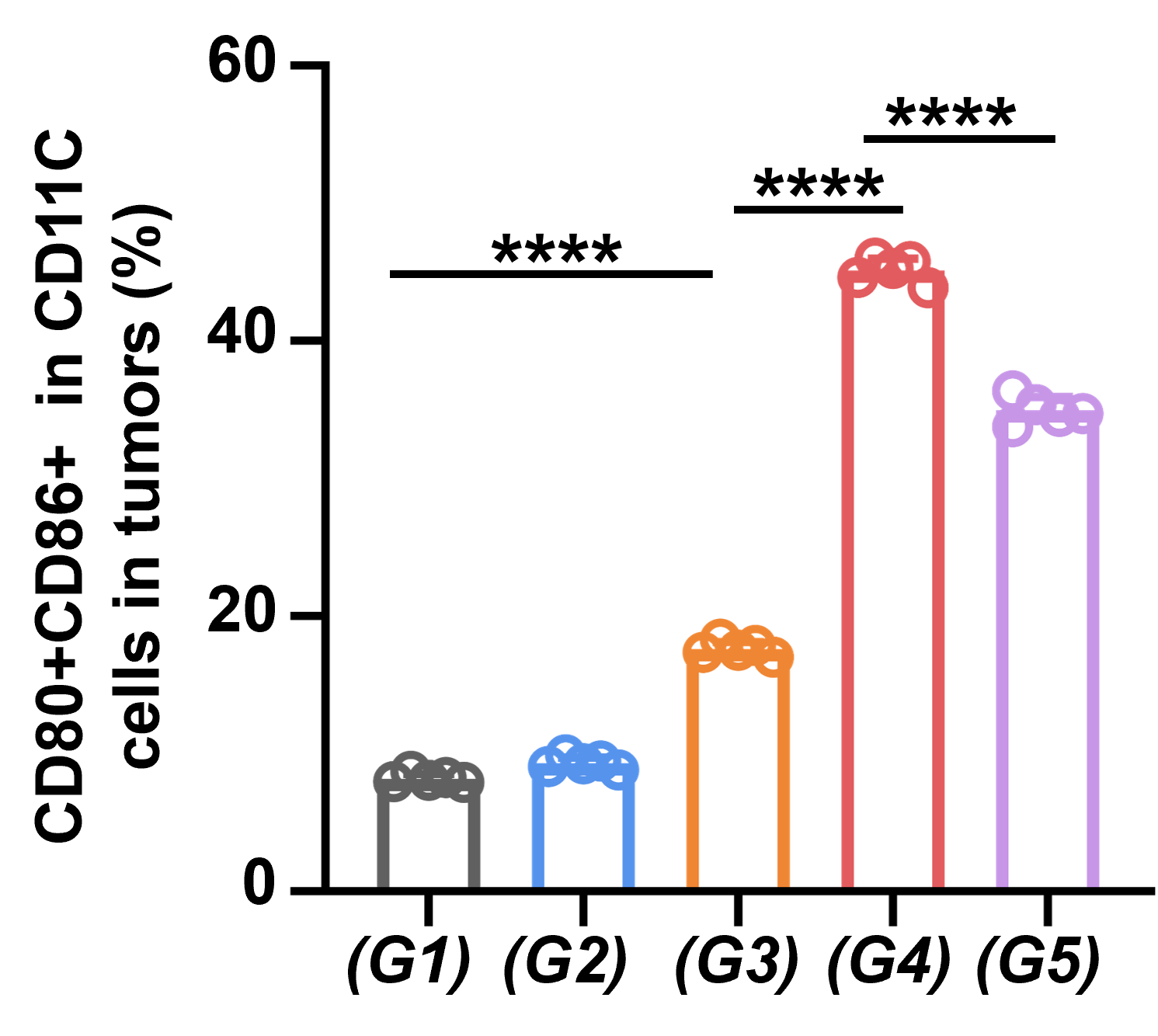
**

**Figure S19.** Relative quantification of flow cytometry analysis for the CD80^+^CD86^+^ cells in CD11C^+^ cells in tumors of mice on day 10 (Data represent: mean ± SD, n=5) ns: no significant difference, *p < 0.05, **p < 0.01, ***p < 0.001, ****p < 0.0001 as determined by one-way ANOVA followed by Tukey's multiple comparison.

**
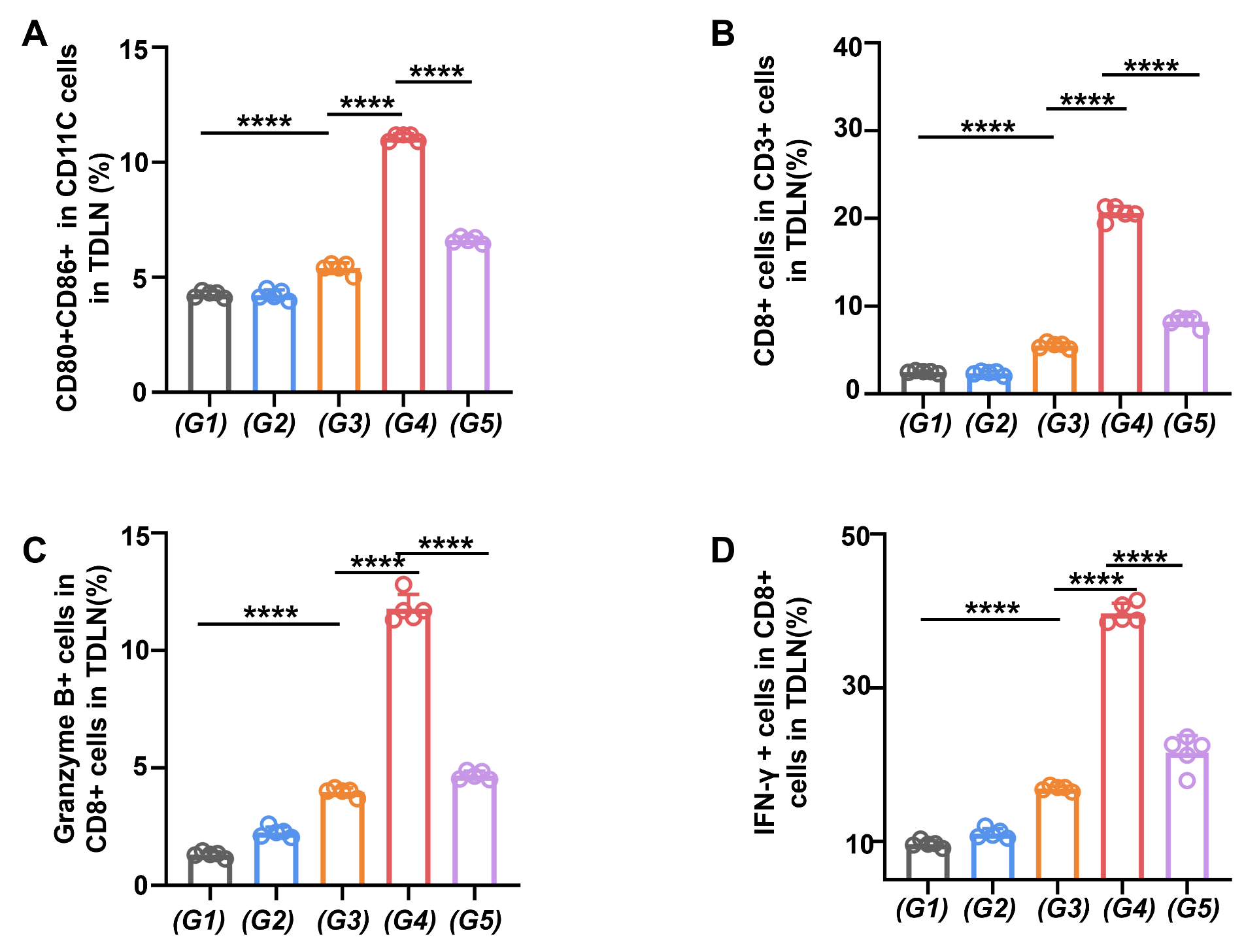
**

**Figure S20. Flow cytometric quantification of mature DCs, GZMB^+^/CD8^+^ T cells, IFN-γ^+^/CD8^+^ T cells in tumor-draining lymph nodes (TDLNs) of mice after different treatments. A)** Relative quantification of flow cytometry analysis for the CD80^+^CD86^+^ cells in CD11C^+^ cells in TDLNs of mice on day 10 (Data represent: mean ± SD, n=5). **B)** Relative quantification of flow cytometry analysis for the CD8^+^ cells in CD3^+^ T cells in TDLNs of mice on day 10 (Data represent: mean ± SD, n=5). **C)** Relative quantification of flow cytometry analysis for the GZMB^+^ in CD8^+^ T cells in TDLNs of mice on day 10 (Data represent: mean ± SD, n=5). **D)** Relative quantification of flow cytometry analysis for the IFN-γ^+^ in CD8^+^ T cells in TDLNs of mice on day 10 (Data represent: mean ± SD, n=5) ns: no significant difference, *p < 0.05, **p < 0.01, ***p < 0.001, ****p < 0.0001 as determined by one-way ANOVA followed by Tukey's multiple comparison (A, B, C, D)

**
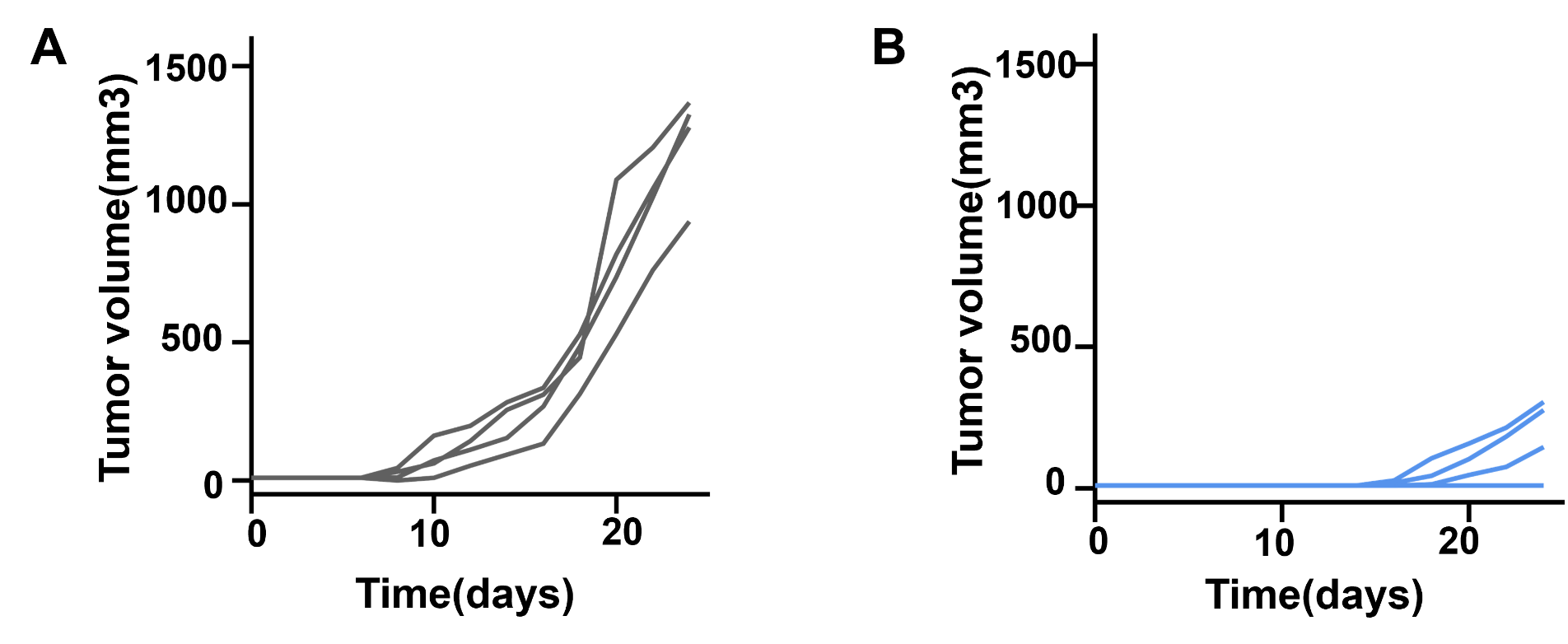
**

**Figure S21.** Rechallenged tumors growth curves for individual mice in the B16F10-LUC rechallenge model (A) Naive mice, (B) Cured Mice (n=4).

**
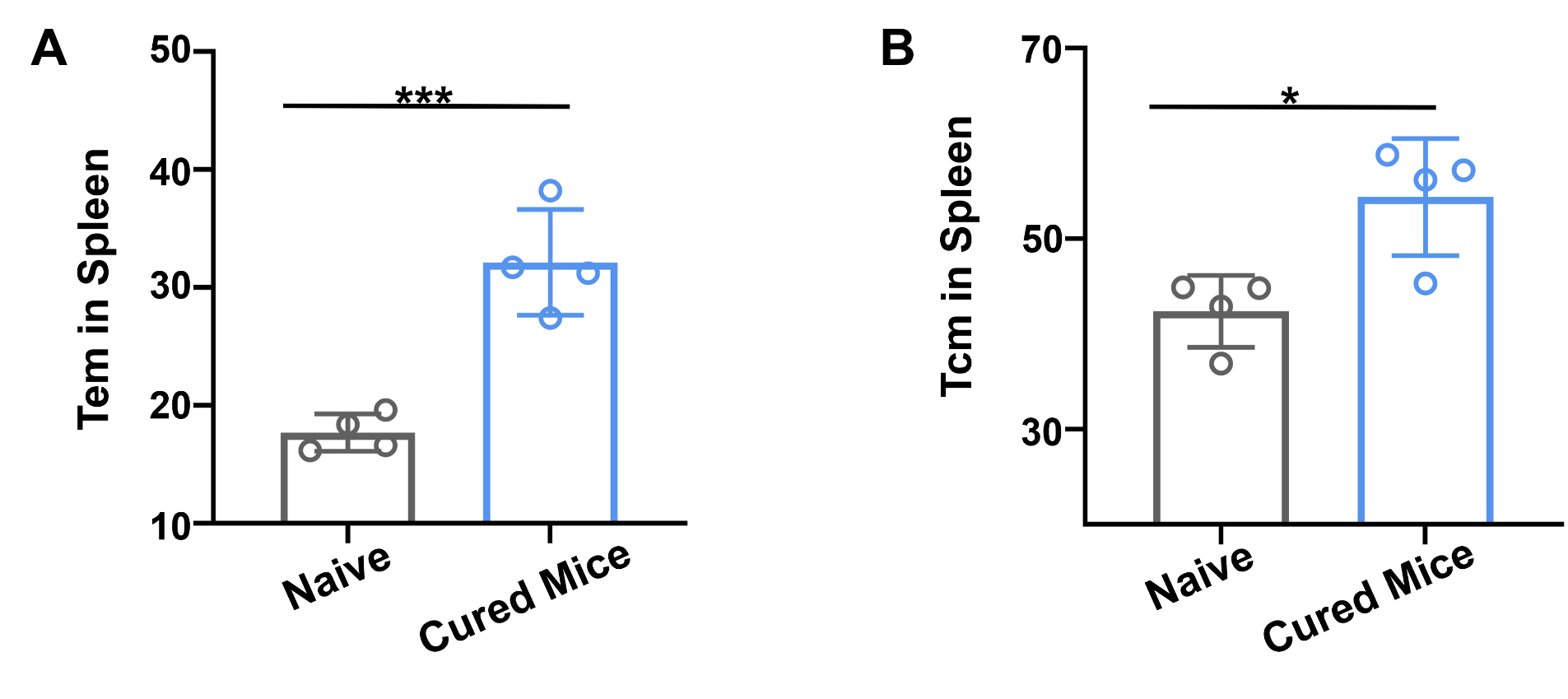
**

**Figure S22.** Representative flow cytometry analysis and quantification results of (A) T_em_ cells (CD3^+^CD8^+^/CD44^+^/CD62L^-^) and (B) T_cm_ cells (CD3^+^CD8^+^/CD44^+^/CD62L^+^) cells within spleens in the B16F10 rechallenge model (Data represent: mean ± SD, n=4) ns: no significant difference, *p < 0.05, **p < 0.01, ***p < 0.001, ****p < 0.0001 as determined by two-tailed unpaired Student's t-test (A, B).

**
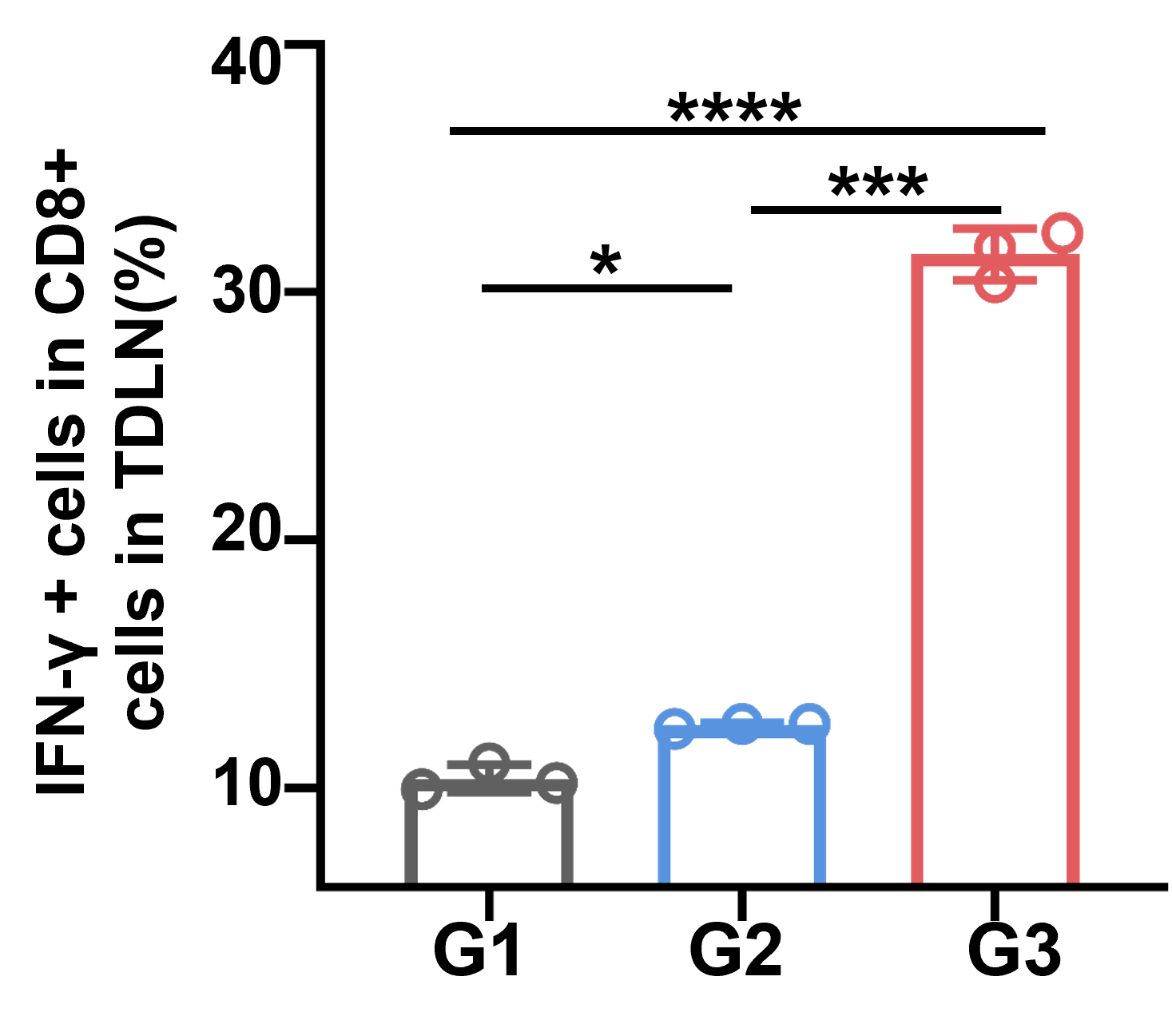
**

**Figure S23.** Relative quantification of flow cytometry analysis for the IFN-γ^+^ in CD8^+^ T cells in TDLNs of mice on day 10 (Data represent: mean ± SD, n=3) ns: no significant difference, *p < 0.05, **p < 0.01, ***p < 0.001, ****p < 0.0001 as determined by one-way ANOVA followed by Tukey's multiple comparison.

**
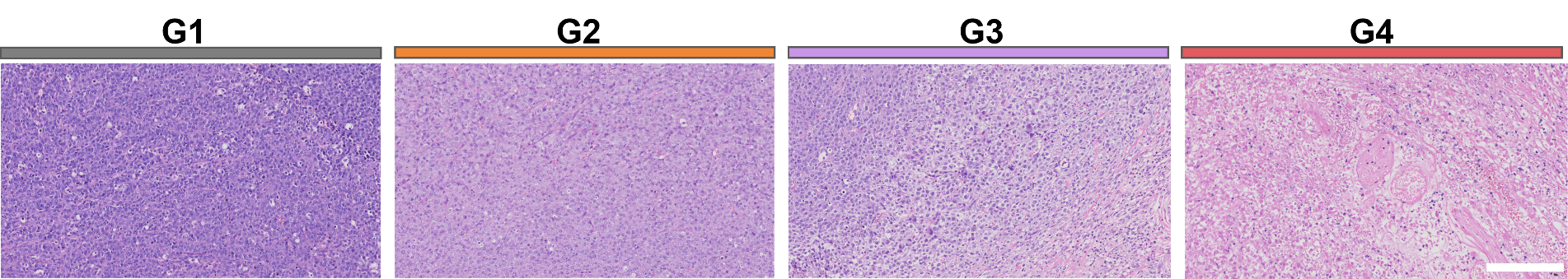
****Figure S24.** H&E staining of RM-1 tumor tissues from the mice receiving different treatments. Scale bars: 200 μm.


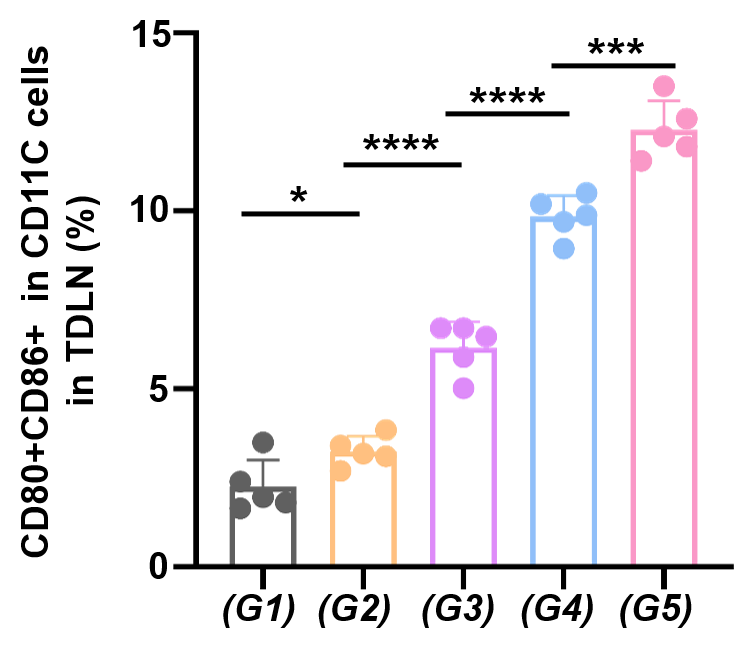


**Figure S25.** Relative quantification of flow cytometry analysis for the CD80^+^ CD86+ in CD11C^+^ cells in tumor of mice on day 10 (Data represent: mean ± SD, n=5) ns: no significant difference, *p < 0.05, **p < 0.01, ***p < 0.001, ****p < 0.0001 as determined by one-way ANOVA followed by Tukey's multiple comparison.

**
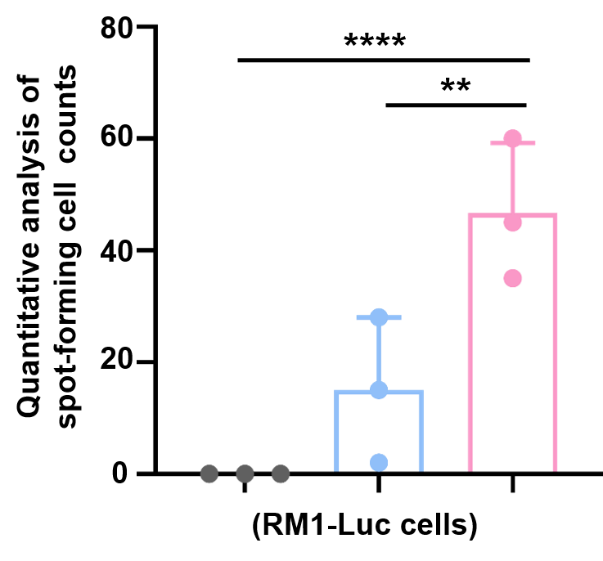
**

**Figure S26.** ELISpot quantification of IFN‑γ‑secreting activated T cells (Data represent: mean ± SD, n=3) ns: no significant difference, *p < 0.05, **p < 0.01, ***p < 0.001, ****p < 0.0001 as determined by one-way ANOVA followed by Tukey's multiple comparison.

**
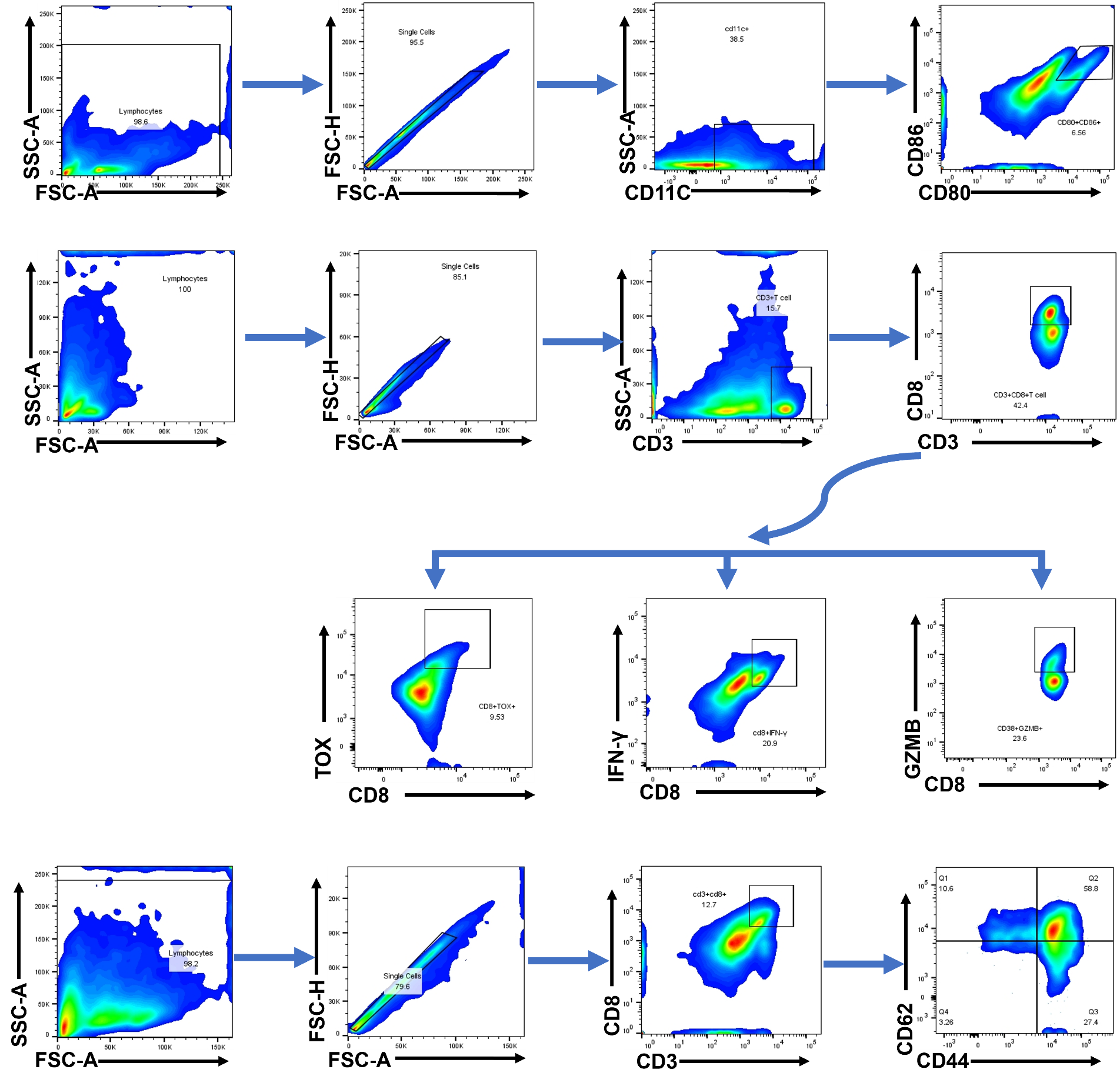
**

**Figure S27.** The gating strategy for flow cytometry analysis of mature DCs, T_em_, T_cm_, CD3^+^, CD8^+^, Granzyme B^+^/CD8^+^ T cells, TOX^+^/CD8^+^ T cells and IFN-γ^+^/CD8^+^ T cells in the tumor and lymph node tissues from the mice receiving different treatments.

**References**

[1] G. B. Ferraro, A. Ali, A. Luengo, D. P. Kodack, A. Deik, K. L. Abbott, D. Bezwada, L. Blanc, B. Prideaux, X. Jin, J.M. Posada, J. Chen, C. R. Chin, Z. Z. Amoozgar, R. Ferreira, I.X. Chen, K. Naxerova, C. Ng, A.M. Westermark, M. Duquette, S. Roberge, N.I. Lindeman, C.A. Lyssiotis, J. Nielsen, D.E. Housman, D.G. Duda, E. Brachtel, T.R. Golub, L.C. Cantley, J.M. Asara, S.M. Davidson, D. Fukumura, V.A. Dartois, C.B. Clish, R.K. Jain, M.G. Vander Heiden, Fatty Acid Synthesis is Required for Breast Cancer Brain Metastasis. *Nat Cancer*. **2021**, 2, 414.
